# Predictors and prevalence of support for white nationalism in the United States

**DOI:** 10.1038/s41586-026-11018-0

**Published:** 2026-09-16

**Authors:** Patrick J. Egan, James Bisbee, Joshua A. Tucker

**Affiliations:** 1 New York University, New York, NY USA; 2 New York University Center for Social Media, AI and Politics, New York, NY USA; 3 Vanderbilt University, Nashville, TN USA

**Keywords:** Politics, Sociology, Human behaviour

## Abstract

White nationalism has become increasingly visible in American public life^1–10^. Yet we lack knowledge of the predictors and prevalence of explicit support for this ideology, as survey research has treated it as a latent construct captured through indirect indicators^11–15^. We introduce and validate a measure that informs respondents of the core tenets of the white nationalist movement and then asks whether they support the movement. Expressed support is distinct from organizational affiliation with the movement, which we do not measure. Here we show with three national surveys of non-Hispanic white adults (conducted from 2021 to 2025) that support for white nationalism is (1) higher among white people encountering personal hardship and local social distress, consistent with strain theories^3,16^; (2) unrelated to local racial composition or recent demographic change, complicating status threat accounts^4,17,18^; and (3) elevated among white people with close friendships primarily online, consistent with theories of extremism driven by online mobilization^7,10,19^. Support for white nationalism is more common among white people with lower incomes and education, Republicans and conservatives, and those who are politically disaffected. In our national probability sample, prevalence of support was estimated at 4.9% of white adults, including 13.5% of white men aged 18–29. Support for white nationalism seems to constitute a discrete dimension of white racial attitudes, and many white people who endorse its core ideas are unaware of the label.

## Abstract (web summary)

Explicit support for white nationalism is uncommon overall, but higher among young white men, disadvantaged and politically disaffected white people, conservatives, Republicans, and those with primarily online friendships.

## Main

White nationalism has become more visible in American public life in recent years. Those who call themselves white nationalists believe that shifting demographics pose an existential threat to the white race, which they view as culturally superior and deserving of power over all other groups in the United States. A loosely organized white nationalist movement operates largely out of view through paramilitary groups, small gatherings, white power music performances and online networks, inspiring the lone actors who have committed many of the nation’s most murderous acts of racial terror. Movement participants periodically emerge into the public eye with rallies and other direct actions, some of which are linked to political violence^1–10^.

Yet we know little about the predictors and prevalence of white nationalist views in the wider US white population. It is possible that many white people are unaware of this term, but are nevertheless sympathetic to white nationalist ideology. The scholarly literature employing surveys to assess support for white nationalism has thus far construed support for the movement as a latent construct to be measured indirectly (without using the term white nationalism in survey questions). It has also considered white nationalism as a predictor of other attitudes—including opinions about political violence, criminal justice, immigration and the COVID pandemic—rather than as a phenomenon to be explained in its own right^11–15^. We note here that white nationalism is distinct from Christian nationalism—an ideology that seeks a fusion of Christianity and American civic life^20^, and whose tenets are endorsed by nearly equal shares of white and non-white people^21^.

We introduce a new measure of support for white nationalism that defines this term for respondents and then asks whether they support the white nationalist movement. Our strategy is to assess whether respondents endorse a statement summarizing core beliefs associated with white nationalist ideology when presented with that statement in a survey context. Endorsement of the statement is distinct from organizational affiliation with the white nationalist movement, which these data do not measure. Our findings contribute to a growing body of work employing national sample surveys to examine the politicized nature of white identity in the US^22–25^, including white nationalism^9,11–15^.

Our new measure represents progress on past survey research on white nationalism. We ask respondents directly whether they support white nationalism, which permits comparisons of prevalence estimates across population subgroups and—if used in future research—assessments of any over-time change in incidence of support among Americans. At the same time, our approach reduces the measurement error we would expect to arise from lack of awareness by informing survey takers of the key beliefs of the white nationalist movement before asking if they support it.

We present results from three national surveys of non-Hispanic white US adults (studies 1, 2 and 3) that establish the measure’s validity and address concerns on measurement error and response bias. We provide estimates of the correlates and prevalence of support for white nationalism in the American white population. We then assess the evidence for three sets of theoretically grounded explanations for why white people might endorse white nationalist beliefs: strain, status threat and online mobilization theories. We draw these theories in part from scholarship tracing the development of extremism across the political spectrum^26,27^, as we hypothesize that similar dynamics may explain the conditions under which white nationalist ideology gains appeal. Our cross-sectional data constrain our ability to make causal claims regarding these theories; these results should thus be considered corroborative evidence that suggests avenues for future research.

The first set of explanations is strain theories, which centre on the individual and posit that various types of strain—including shocks such as personal health crises, crime victimhood or the dissolution of a relationship or job—create vulnerabilities that make individuals more receptive to radicalizing messages^3,16^. Many Americans, including white people, are experiencing individual and group-level economic hardships of the kind identified by strain theories as once widespread social and economic mobility has declined^28^, and job losses in manufacturing and related industries are linked to increased social distress^29^.

The second set of theories adopts a wider lens, focusing on groups. These status threat explanations hold that real or perceived diminishment of a group’s relative power can fuel grievance narratives that cast blame on out-group members^4,17,18^. A steady decline in the share of white people in the US population—combined with changes to America’s racial and ethnic order^30,31^—has contributed to a diminishment of the once-dominant group status and near-absolute political power of white people.

A final set of hypotheses focuses on the role of the internet, and in particular how extremist groups exploit strain and status threat via online mobilization to disseminate their messages and recruit adherents^7,10,19^. Technological shifts have created opportunities that online mobilization theories hold are favourable for the dissemination of white nationalist ideology. The internet and social media lack the filters and editorial gatekeeping of legacy media, making it easier to access white nationalist content and be incidentally exposed to it^10,19,32,33^. Furthermore, online networks can be appealing to those who are estranged from offline communities and come to rely on the internet for their most important social relationships. Online life can thus be both cause and consequence of these users’ alienation.

One implication of all three of these theories is that white nationalist ideology may be distinctively appealing to young white men. Although some previous work has found that the youngest white cohorts register low support for ‘old-fashioned racism’ in national surveys relative to older white people^34^, young people currently face the highest potential exposure of any age group to online mobilization efforts, given that they are the most likely to say they use the internet almost constantly, and get their news from social media rather than traditional news sources^35^. Young generations of white men, in particular, may find white nationalism’s grievance narrative especially appealing given that they are worse off today than previous white male cohorts on many measures of engagement, achievement and wellbeing—including college completion, labour force participation and suicidality^36^.

## Overview of the studies

To probe these explanations and document the extent of white nationalist beliefs, we fielded three original national surveys of non-Hispanic white US adults in 2021 (study 1), 2024 (study 2) and 2024–2025 (study 3) (refer to the Methods for further details; Extended Data Tables 1, 2 and 3 report summary statistics for the three studies; Supplementary Information A and B report the full questionnaire text for all surveys and further relevant statistics, respectively).

Study 1 was an extensive survey fielded to *N* = 3,227 non-Hispanic white people in November 2021 as part of the Cooperative Election Study (CES), a widely used source of data in peer-reviewed political science research on US public opinion. The CES’s in-depth Common Content questionnaire and respondent geocodes permit documentation of the demographic and political predictors of support for white nationalism and exploration of our theories. The CES was conducted on the web by the YouGov survey firm; respondents were selected from an opt-in panel using a sample matching and weighting methodology that yields estimates aiming to approximate those obtained from nationally representative probability samples of US adults^37^. This approach assumes that, conditional on the demographic variables used for weighting, panel participation is independent of the attitudes measured here. We note that this assumption cannot be directly verified, particularly for stigmatized political opinions and identities.

Study 2 confirmed that key demographic and political results from study 1 held with a high-quality national sample recruited offline via probability-based methods. We conducted this brief nationally representative web-based survey of *N* = 2,114 non-Hispanic white people in May and June 2024 via the National Opinion Research Corporation’s (NORC’s) AmeriSpeak panel^38^. AmeriSpeak randomly selects households from a national sampling frame using area probability and address-based sampling. Sampled households are recruited into the panel by US mail, telephone and field interviewers. We base our overall prevalence estimates on results obtained from this high-quality sample using the sampling weights supplied by NORC.

Study 3 was a supplementary survey fielded to a national quota-based sample of *N* = 2,548 non-Hispanic white people recruited by CloudResearch^39^ in late 2024 and early 2025. We conducted study 3 to document relationships between our measure of support for white nationalism and extant constructs of racial attitudes established in previous scholarly work, as well as to rule out concerns about survey response bias with a series of question-wording experiments.

## Measuring support for white nationalism

Instead of explicitly using the white nationalism term in survey items, past scholarly research has assessed support for white nationalism implicitly using indices of items measuring white peoples’ beliefs in ethnic homogeneity, racial superiority and, in some batteries, immigration restrictions^11–15^. Here we introduce a new approach that yields estimates of the prevalence of explicit support for the white nationalist movement among people who have been informed of its core principles.

Our strategy anticipated that some respondents might be unaware of white nationalism and thus might misinterpret survey questions about this term. We therefore incorporated a question-wording experiment in studies 1 and 2 that allowed us to ascertain the impact of being informed of the meaning of the term on expressed support for the movement while also collecting fully informed responses among our entire sample.

In both studies, half of our respondents were chosen at random to be in the treatment group, whose members read a brief description of the white nationalist movement focused on three intertwined tenets—demographic threat, the maintenance of power and cultural superiority—that are commonly invoked by self-described white nationalists and have been identified by analysts as central to white nationalist ideology^1,2,5–8,10–15^. The statement was limited to beliefs, and did not discuss tactics (such as support for intimidation or violence) or behaviour (such as respondents’ participation in actions or movements).

The description read: “The white nationalist movement holds that whites are under threat in the United States and seeks to ensure the survival of the white race in this country. White nationalists believe that white people should hold more political and economic power than other groups. They believe that whites should maintain their majority in the US and that white culture is superior to all other cultures”.

After being presented with the description, respondents in the treatment condition were then asked, “Are you a supporter of the white nationalist movement?”. The other half of the respondents were assigned to a control group. They were first asked the question “Are you a supporter of the white nationalist movement?” and their responses were recorded. They were then presented with the short description offered to the treatment group and asked the question again. In all cases, participants were given the binary response set of yes or no; we counted non-responses as no to keep our estimates of the proportion of white people endorsing white nationalism as conservative as possible.

**Fig. 1:**
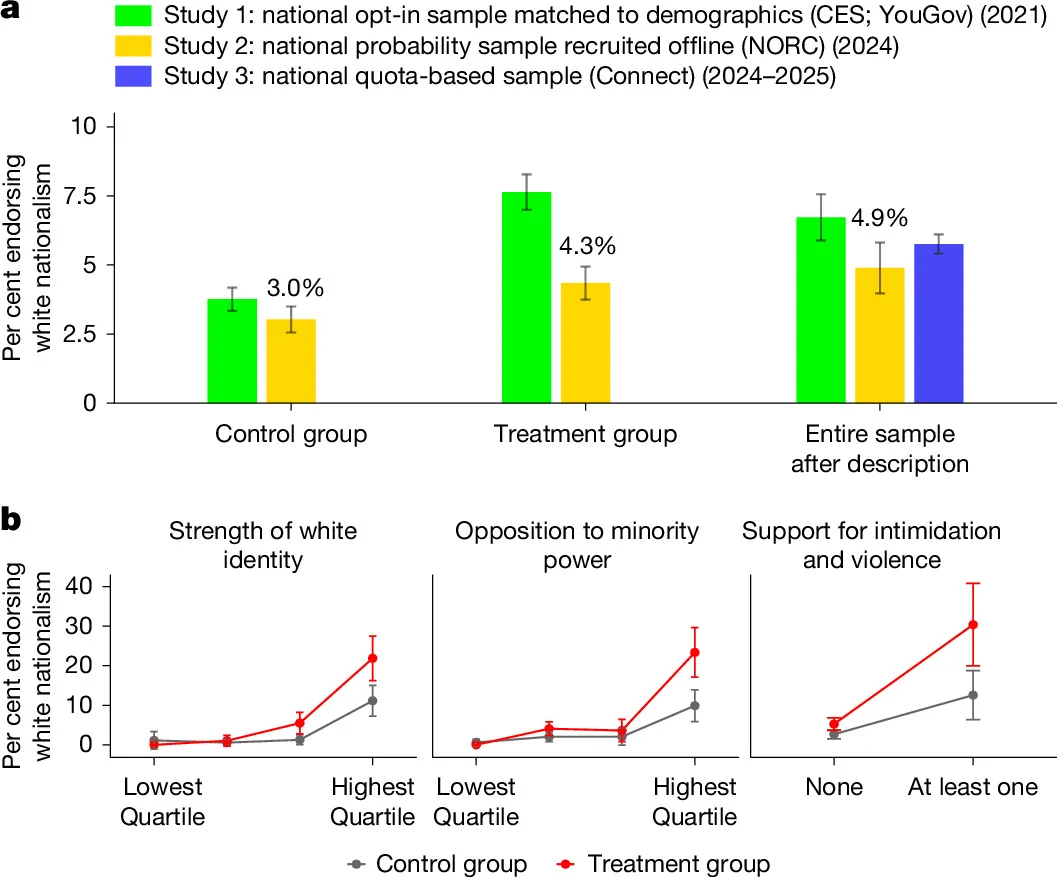
Endorsement of white nationalism among US non-Hispanic white adults and validation of our measure. **a**, Endorsement of white nationalism by white people in the experimental control group (no description of white nationalism provided), treatment group (description provided) and among all respondents after the description was provided. The experiment was not conducted in study 3. Control-versus-treatment differences were tested with two-sided weighted two-proportion *z*-tests (study 1, *P *< 0.001; study 2, *P* = 0.25). Bars are weighted means; error bars are ±1.39 × the standard error of the mean (s.e.m.), an interval whose non-overlap approximates a two-sided *α* = 0.05 difference test between independent means^54^. Yellow bars denote study 2 (national probability sample, NORC, 2024; *n*_control_ = 1,067, *n*_treatment_ = 1,039, *n*_entire_ = 2,114); green bars denote study 1 (national opt-in sample matched to demographics, CES/YouGov, 2021; *n*_control_ = 1,617, *n*_treatment_ = 1,610, *n*_entire_ = 3,227); and blue bars denote study 3 (national quota sample, Connect, 2024–2025; *n*_entire_ = 771). **b**, Heterogeneous treatment effects in study 1 by strength of white identity (left, *n* = 3,011); opposition to minority group power (centre, *n* = 3,226); and support for intimidation, harassment and violence against non-white people (right, *n* = 3,205). Points are weighted means; lines connect treatment arms within moderator level; error bars are ±1.96 × s.e.m. Grey points denote the control group; red points denote the treatment group. Within-level treatment-versus-control contrasts were tested with two-sided weighted two-proportion *z*-tests; exact *P*-values for all contrasts are reported in the Source data. No adjustment for multiple comparisons was applied. Refer to Supplementary Information C for details. Source data

Our experimental results suggest that some white Americans are unfamiliar with the term white nationalism, but when informed of its meaning they endorse it (Fig.  1a). In study 1, among respondents in the control group of our survey experiment (who were initially not provided any description of white nationalism), 3.8% said they supported the white nationalist movement. This number rose to 7.6% among those in the treatment group (who were shown the description before being asked for a response)—a rate significantly different from that in the control group (*P* < 0.001). In study 2, which was conducted on the probability-based sample, the description treatment did not produce a statistically significant difference in endorsement (3.0% control versus 4.3% treatment; a difference of 1.3 percentage points (pp), with a 95% confidence interval [−0.89, 3.52], *P* = 0.25). In interpreting this result, we note that an equivalence test on this effect failed, meaning that we cannot definitively rule out a substantively meaningful effect in study 2 (Methods). We further note that the minimal detectable effect at 80% power in study 2 was approximately 2.5 pp, making this experiment adequately powered to detect the 3.8 pp effect observed in study 1. The divergence between studies 1 and 2 should be considered when interpreting the description treatment as informational rather than wording-induced.

## Establishing measurement validity

Study 1 established criterion validity for our measure of support for white nationalism with respondents’ answers to three batteries of questions measuring constructs related to white nationalism adapted from previous research: (1) respondents’ strength of white identity and their support for white collective action; (2) respondents’ evaluations of the level of power held in the US by different racial and religious groups; and (3) respondents’ support for intimidation, harassment and the use of violence to advance white political goals. Refer to the Methods for question wording.

Responses to these batteries show that the gap between treated and control respondents was due to the fact that providing a description of the term white nationalism led white people who were previously uninformed about the meaning of the term to align their now informed responses with their other racial attitudes. As shown in Fig. 1b, this gap was 22 pp larger among white people expressing strongest levels of white racial identity (*P* < 0.001), 29 pp larger among white people registering highest agreement with statements that non-white and non-Christian groups have ‘too much power’ (*P* < 0.001); and 27 pp larger among white people most strongly supporting acts of intimidation, harassment and violence against non-white people (*P* < 0.10). Refer to Supplementary Information C for details on the tests.

The fact that survey participants’ support for white nationalism in the treatment condition was more strongly associated with measures of related outcomes corroborates the criterion validity of participants’ fully informed responses. These findings indicate that informed responses were subject to less measurement error than uninformed responses, and thus help to address the concern that such error may artificially inflate estimates of support for low-prevalence extremist views^40^.

Our measure bears some similarities with other established measures of racial attitudes developed by scholars in political science, sociology and psychology (Methods). But no extant measure explicitly invokes the white nationalist movement’s claims about demographic threat and cultural superiority, coupled with calls to preserve white political power. In study 3 we found the expected positive but relatively low correlations (ranging from *r* = 0.15 to *r* = 0.40; Extended Data Table 4) between our measure and each of these constructs, suggesting that we are measuring a phenomenon adjacent to, but crucially distinct from, these established constructs of racial attitudes (Supplementary Information D).

## Investigating upward bias

We investigated, and ruled out, the concern that our estimates of support might be biased upward by three different factors: the responses of inattentive participants, acquiescence bias and priming effects (refer to the Methods for description of tests and Supplementary Information E for results). First, we examined the relationship between the speed with which respondents completed our surveys (which previous work has shown is correlated with inattentiveness^41^) and their support for white nationalism. We found any upward bias due to inattentive respondents to be minimal in study 1 (reducing prevalence estimates by less than a percentage point) and non-existent in study 3 (all Bayesian information criterion (BIC)-based model comparisons returned ΔBIC > 20; Methods). Second, a question-wording experiment using a reverse-keyed item in study 3 found no evidence that prevalence estimates from our measure are inflated by acquiescence bias (Supplementary Information F). The reverse-keyed item, in fact, elicited endorsement rates of white nationalism that were 9 pp higher than our original item (*P* < 0.001; Extended Data Table 5). Third, we found no evidence that priming effects inflated prevalence estimates in a question-order experiment in study 3 randomly placing our white nationalism item at the beginning or end of our questionnaire (where responses might be primed by our batteries of questions on racial attitudes; Supplementary Information F). Support for the white nationalism movement was no different when the item was placed at the beginning of the survey rather than at the end (*P* = 0.88; Extended Data Table 5). An equivalence test on this estimate passed, meaning that we can definitively rule out a substantively meaningful association between item placement and expressed support for white nationalism (Methods).

## Support for white nationalism in the US

### Demographic and political predictors

**Fig. 2:**
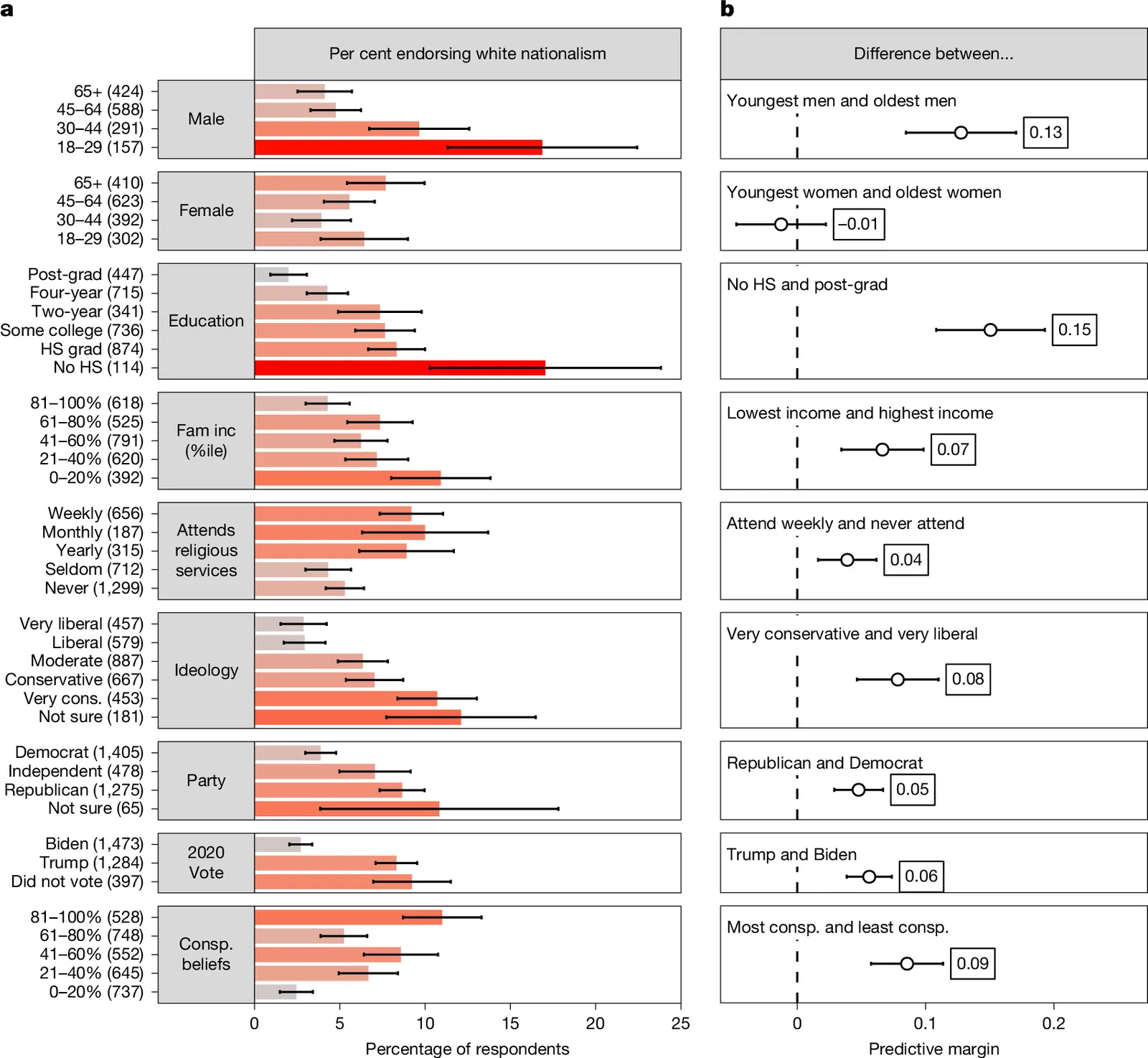
Correlates of endorsement of white nationalism. **a**, Weighted prevalence of endorsement of white nationalism (*x* axis) by demographic and political covariates (*y* axis) in study 1 (national opt-in sample matched to demographics; CES/YouGov, 2021; per-covariate *n* ranges from 1,460 to 3,227; refer to the Source data for the exact *n* per covariate). Bars are weighted means using CES survey weights. Bar fill intensity (light grey to red) indicates prevalence of endorsement. The unweighted within-level *n* is shown in parentheses on the *y* axis. Error bars are 84% confidence intervals (± 1.39 × s.e.m., an interval whose non-overlap approximates a statistically significant pairwise difference in means^54^ at two-sided *P* < 0.05). **b**, Predictive margins from bivariate weighted OLS regressions of endorsement on each covariate in study 1 (the per-regression *n* is reported in the Source data). Each point is the OLS estimate of the difference in probability of endorsing white nationalism between the maximum and minimum level of the predictor; the specific contrast is indicated at the top of each facet. The dashed vertical lines mark null effects (zero difference). Numeric labels to the right of each error bar give the exact point estimate. Error bars are 95% confidence intervals (±1.96 × s.e.m.). Exact two-sided *P*-values—unadjusted for multiple comparisons—are reported in the Source data. HS, high school; Consp., conspiracy; grad, graduate; Fam inc, family income. Refer to Supplementary Information G for details. Source data

**Extended Data Fig. 1:**
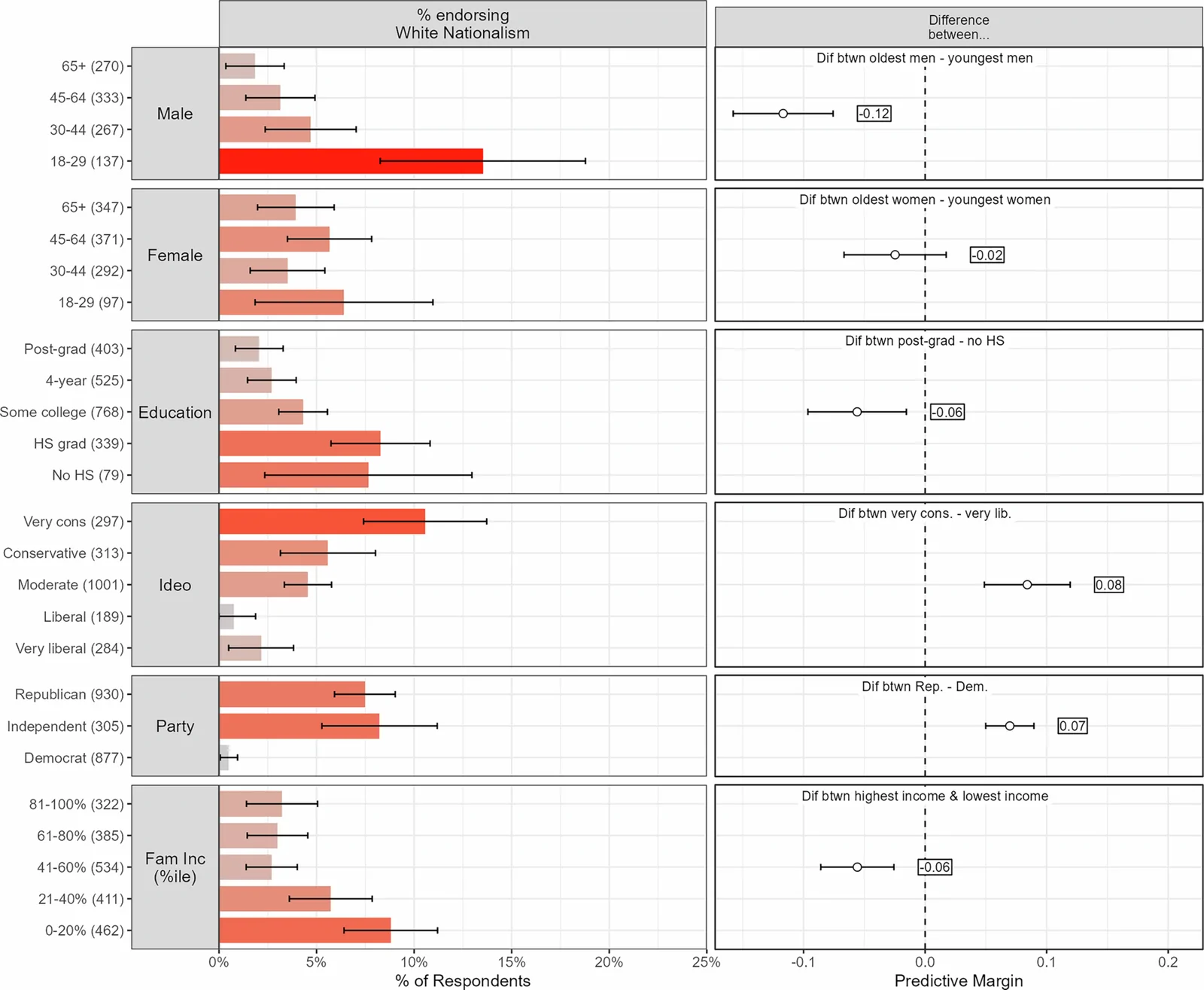
Replication of tests of predictors of support for white nationalism in Study 2. Left: Weighted prevalence of endorsement of white nationalism (x-axis) by demographic and political covariates (y-axis) in Study 2 (national probability sample of non-Hispanic white adults, NORC/AmeriSpeak, 2024). Bars are weighted means using AmeriSpeak survey weights. Bar fill intensity (light grey to red) indicates prevalence of endorsement. Unweighted within-level *n* shown in parentheses on the y-axis. Error bars are 84% confidence intervals ( ± 1.39 × the standard error of the mean (s.e.m.), an interval whose non-overlap approximates a statistically significant pairwise difference in means^54^ at two-sided *P* < 0.05). Right: Predictive margins from bivariate weighted ordinary least squares regressions of endorsement on each covariate in Study 2 (per-regression *n* ranges from 1,007 to 2,114; see Supplementary Information Table S18 for exact *n* per regression). Each point is the OLS estimate of the difference in probability of endorsing white nationalism between the maximum and minimum level of the predictor; the specific contrast is indicated at the top of each facet. The dashed vertical line marks null effects (zero difference). Numeric labels to the right of each error bar give the exact point estimate. Error bars are 95% confidence intervals (± 1.96 × s.e.m.). Exact two-sided *P*-values, unadjusted for multiple comparisons, are reported in Supplementary Information Table S18. See Supplementary Information section G for details. Source data

Our analyses of white nationalist views reported throughout the remainder of this article are based upon all fully informed responses (that is, responses from those in the treatment group pooled with those in the control group after they were provided the description of white nationalism). Figure 2 displays the relationships between white nationalist support and key demographic and political characteristics found in study 1 and estimates of their statistical significance. Where data were available we replicated these findings with study 2’s high-quality national probability sample (Extended Data Fig. 1). Supplementary Information G has details about all estimates and statistical tests reported here.

Study 1 revealed a significant (*P* < 0.001) negative relationship between age and white nationalist support among men. Young men (aged 18–29) registered support at a rate 13 pp higher than those aged 65 and over. No such relationship between age and white nationalism was found among women (*P* = 0.37). These findings were replicated in study 2, where again a negative, statistically significant relationship between age and support for white nationalism was found among men (a 12-pp gap; *P* < 0.01) and not women (*P* = 0.87). Bayesian-information-criterion-based model comparisons returned very strong evidence in study 1 (ΔBIC ≈ 29) and strong evidence in study 2 (ΔBIC ≈ 6) in favour of the null that age is not a predictor of support among women (Methods).

Support for white nationalism in study 1 was significantly negatively associated with educational attainment (a 15-pp gap was found between those at the minimum and maximum levels of this predictor; *P* < 0.001) and income (a 7-pp gap; *P* < 0.02), findings that we replicated in study 2 (6 pp at *P* < 0.01 and 6 pp at *P* < 0.001, respectively). Study 1 also measured white peoples’ attendance of religious services; attendance was significantly positively associated with endorsement of white nationalism, a 4-pp difference between respondents at the minimum and maximum levels (*P* < 0.01).

Endorsement of white nationalism in study 1 was 8 pp higher among conservatives than liberals and 5 pp higher among Republicans than Democrats. We replicated these findings in study 2 (at 8 pp and 7 pp, respectively; *P* < 0.001 for all tests in both studies). Study 1 found voters for Donald Trump in the 2020 election were 6 pp more likely to support white nationalism than voters for Joe Biden (*P* < 0.001). Study 1 also found higher endorsement among politically disaffected white people, as shown by the elevated rates of support among non-voters (3 pp higher than voters, *P* < 0.06) and white people not sure about their ideology (6 pp higher than all those placing themselves on the ideological scale; *P* < 0.12). Moreover, study 1 found white nationalist views significantly more prevalent among white people who engage in conspiracy thinking about politics and government (a 9-pp gap between those at the maximum and minimum levels of this scale; *P* < 0.001).

These bivariate analyses are descriptive. When white nationalist support is modelled with multiple regressions incorporating the demographic and political covariates presented in Fig. 2, all of these associations except income remain consistent holding all other covariates constant (Extended Data Table 6; details in Supplementary Information H). However, even our multivariate results should not be interpreted as causal claims, because there are threats to inference associated with observational data such as unobserved confounders and reverse causation.

A possible concern with our findings is that the relationships reported here may be artefacts of acquiescence bias, which may confound the relationship between support for white nationalism and its predictors. We explored these concerns with the question-wording experiment in study 3 described above (Extended Data Table 7; details in Supplementary Information G), which confirmed that statistically significant relationships (*P* < 0.05) between white nationalist support and the covariates reported in Fig. 2 persisted after accounting for acquiescence bias, with the exception of age among men (*P* = 0.16) and education (*P* = 0.15), which fell short of conventional significance thresholds. Data on attendance of religious services were not collected in study 3.

### Prevalence

Our national prevalence estimate is obtained from the high-quality national probability sample in study 2, where the share of non-Hispanic white US adults endorsing white nationalism was 4.9%, 95% CI (3.6, 6.2) (Fig. 1a). Prevalence rose to 13.5% among white men aged 18–29, 95% CI (6.0, 21.0). In interpreting these prevalence estimates, particular attention must be paid to the distinction between endorsing white nationalist views and identification or affiliation with the white nationalist movement itself. Our findings indicate that endorsement of a statement about white nationalist views is broader than self-identification with the term white nationalism, and that some people who endorse the statement may be unfamiliar with the movement. The relationship between statement endorsement and behavioural, organizational or electoral support for the movement remains an open empirical question.

We cannot rule out the possibility that these prevalence estimates were biased downward by non-response bias and social desirability bias. The cumulative response rate reported by NORC for study 2 was 2.7%, a rate similar to other high-quality probability-based sample surveys^42^. To the extent non-response biased our prevalence estimates, we believe it is more plausible that it pushed our estimates downward rather than upward. Rates of general survey response at the time when our surveys were fielded were lower among Republicans^43^, and the less politically aware and engaged^44^, characteristics that we have shown are strong predictors of holding white nationalist views. Further, although the relative anonymity provided by online surveys generally reduces social desirability bias regarding sensitive questions, such bias is not eliminated altogether in these settings^45^.

## Corroborative evidence for theories

**Fig. 3:**
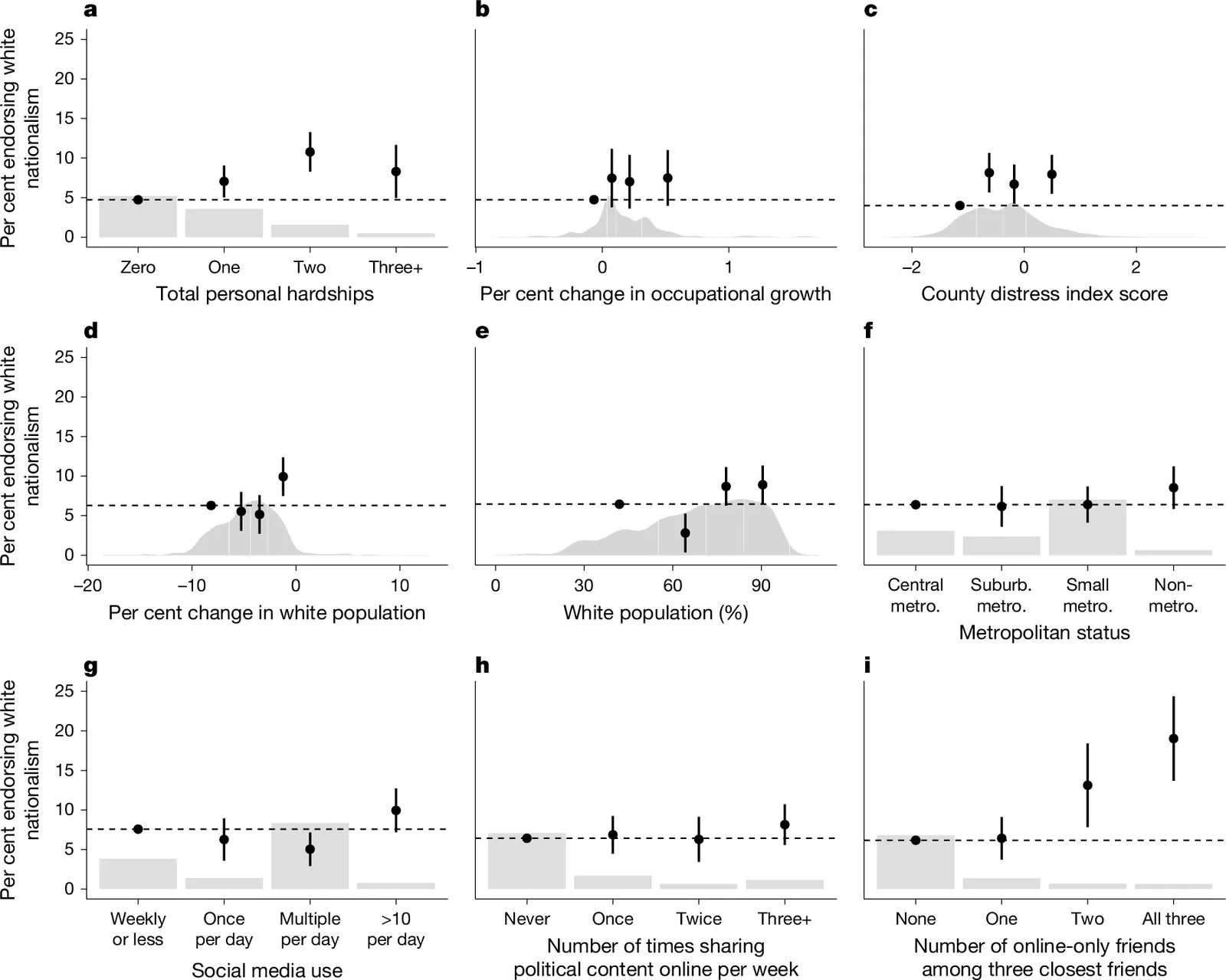
Bivariate associations between endorsement of white nationalism and theories of its origins. **a**–**i**, The weighted predicted prevalence of endorsement of white nationalism in study 1 (national opt-in sample matched to demographics (CES/YouGov, 2021); per-panel *n* is 3,227 (**a**,**h**) 1,430 (**b**), 3,220 (**c–g**) and 2,985 (**i**)) across covariates motivated by three sets of theories: strain (top row, **a**–**c**), status threat (middle row, **d**–**f**) and online mobilization (bottom row, **g**–**i**). Each panel reports estimates from a separate bivariate weighted OLS regression of endorsement on the covariate, using CES survey weights. For continuous covariates (**b**–**e**), the predictor is cut into within-sample quartiles and the point for each non-reference quartile is plotted at the within-quartile mean of the predictor. For ordinal or categorical covariates (**a**,**f**–**i**), points correspond with the named levels on the *x* axes. Points are weighted predicted means; the dashed horizontal lines mark the predicted mean at the reference (lowest, leftmost) level of the covariate, which is shown without error bars. The error bars on the remaining points are 95% confidence intervals (±1.96 × s.e.m.). The grey shading indicates the distribution of the predictor (kernel density for continuous covariates; the bar count of respondents per level for categorical covariates). The exact two-sided *P*-values from the OLS contrasts and the overall regression *F*-test—unadjusted for multiple comparisons—are reported in the Source data. Refer to Supplementary Information I for details. Source data

Study 1’s extensive battery of survey responses along with participants’ county-level geocodes allow us to assess corroborative evidence for theories that strain, status threat and online mobilization spur support for white nationalism (Fig. 3; Supplementary Information I has details of all statistical tests).

### Strain

We measure strain at both the micro and macro level. To gauge individual-level strain, our respondents answered questions indicating whether they had suffered any of nine hardships in the previous twelve months, including the death of a loved one, job loss, crime victimization, divorce or the end of a relationship. The number of recent hardships reported by respondents was positively associated with their endorsement of white nationalism, with support 4 pp higher among those reporting three or more hardships in the past year compared with those reporting none (Fig. 3a; *P* < 0.01).

Occupational insecurity is another source of strain. We asked respondents to provide an open-ended description of the nature of their work. Research assistants matched these job descriptions to standard occupational sector codes^46^. We then used historical employment change over the previous ten years by occupational code to characterize each respondent’s degree of labour market risk (Supplementary Information J has details). We found no significant relationship between employment decline in respondents’ occupational sectors and endorsement of white nationalist views (Fig. 3b). An equivalence test on this estimate failed, meaning that we cannot definitively rule out a substantively meaningful association between occupational risk and support for white nationalism (Methods).

To estimate strain at the community level, we used official county-based estimates of poverty rates, unemployment rates, population loss and mortality rates attributed to drugs and alcohol to generate a social distress index with scores assigned to each county in the US (Supplementary Information K). This index is a measure of the local incidence of the kinds of strains attributed by researchers to stressors such as deindustrialization, trade shocks and the opioid epidemic^29^. The level of social distress in respondents’ counties was a significant predictor of respondents’ endorsement of white nationalism, with support 4 pp higher among white people in the most distressed counties compared with those in the least (Fig. 3c; *P* < 0.03). Contextual social distress was itself a significant predictor of white peoples’ number of individual hardships (*P* < 0.02), and both personal hardship and contextual social distress remained significant predictors of support for white nationalism while controlling for the other in a multiple regression analysis (*P* < 0.04; Supplementary Information I).

### Status threat

Using US Census data, we developed measures of levels and recent change in the share of the non-Hispanic white population at the county level (Supplementary Information K). Our expectation from status threat theories was that white people living in places with substantial non-white populations—and in places with recent declines in the white population—would be more likely to experience status threat and thus endorse white nationalist ideology. This expectation was not supported. Although the point estimates indicated greater support for white nationalism in counties with smaller declines in the non-Hispanic white population (Fig. 3d; *P* < 0.08) and lower racial diversity (Fig. 3e; *P* < 0.08), equivalence tests indicated that both associations were smaller than our pre-specified threshold for substantive significance (Methods). We can therefore rule out substantively meaningful associations, in either direction, between county-level racial composition or change and support for white nationalism. The direction of the point estimates is compatible with—but does not provide meaningful support for—accounts emphasizing intergroup contact^47^. These results are consistent with recent work finding mixed associations between local demographic context and racial attitudes^48^. Furthermore, no relationship was found between the rural-urban status of white peoples’ county of residence and their support for white nationalism (Fig. 3f). A BIC-based model comparison returned very strong evidence (ΔBIC ≈ 21) in favour of the null that ruralness of residence is not a predictor of support for white nationalism (Methods).

### Online mobilization

We asked respondents about their frequency of general social media use and their political activity on social media. Neither of these behaviours was associated with endorsement of white nationalism (Fig. 3g,h). Bayesian-information-criterion-based model comparisons returned strong evidence (ΔBIC ≈ 8) in favour of the null that general social media use is not a meaningful predictor of support for white nationalism and very strong evidence (ΔBIC ≈ 23) in favour of the null regarding political social media use (Methods). However, we also asked respondents to list their three closest friends and whether they knew each friend ‘only online’, ‘only offline’ or ‘both online and offline’. Here we found that endorsement of white nationalism was 13 pp higher among respondents reporting that all three of their closest friends were known only online compared with those reporting that none of their closest friends were known only online (Fig. 3i; *P* < 0.02).

### Limitations

These analyses should be considered corroborative evidence for the theories. We cannot conduct tests supporting well-identified causal claims due to confounding and other threats to inference. As a robustness check, we added the three statistically significant theoretical predictors in Fig. 3—personal hardships, local social distress and the number of online-only friends—to a multiple regression that also included all of the demographic and political correlates in Fig. 2 (Extended Data Table 6). All three of these predictors remain significantly associated with support for white nationalism (at a lower threshold of *P* < 0.10) in this context. We also conducted sensitivity analyses exploring the vulnerability of results to omitted variable bias. These analyses show that local social distress as a predictor of white nationalist support is a finding sensitive to such bias, which is less of a concern for our findings regarding personal hardships and online-only friendships (Supplementary Information H).

Many unobserved confounders could conceivably overturn our results. For example, our findings that strain significantly predicts white nationalist support may be due to unmeasured factors—including personality traits, characteristics of upbringing, physical and mental health challenges, and lack of economic opportunity and geographic mobility—that are correlated with both strain and support for white nationalism. Our null findings regarding status threat may in part stem from suppressor bias introduced by white residential sorting into preferred ethnoracial environments. As we discuss further below, the significant association discovered between online friendships and support for white nationalism may be explained by antecedent factors—such as isolation and alienation—that can predispose people to conduct much of their social lives online. Despite these limitations, the tests we conduct here are important first steps in identifying the conditions under which support for white nationalism may be likely to arise.

## Discussion

Here we introduce a new measure of explicit support for white nationalism, provide evidence confirming its validity, and report results from surveys in which the measure was fielded to national samples of non-Hispanic white Americans. Support was higher among white people with less education and lower incomes; among more frequent attenders of religious services; and among Republicans, conservatives and the politically disaffected. White nationalist views were positively correlated with some forms of strain—including personal hardships and local social distress—but not with levels or change in local ethnoracial diversity. And although no relationship existed between social media use and endorsement of white nationalism, a social life lived largely online was a significant predictor of support. We estimate that 4.9% of non-Hispanic white US adults support white nationalism after being informed of its core precepts, a rate that rises to 13.5% among young white men. Beyond these key results, a number of findings have implications that merit further discussion below.

### White nationalism’s public appeal

Through in-depth interviews, ethnography and historical accounts, a rich qualitative literature documents and analyses white nationalism^1,2,5–8,10^ as a loosely organized right-wing social movement^49^ that until recent years has existed largely on the margins in the US. To gauge the extent to which the movement’s core beliefs resonate with the general public, our findings build on past survey research^11–15^ by assessing who explicitly supports white nationalist ideology, quantifying the prevalence of this support and evaluating evidence for theories explaining this ideology’s attractiveness. In doing so, we map the landscape of mass opinion encountered by the white nationalist movement in the United States, demarcating how public attitudes both enable and constrain the movement as it gains visibility and seeks to broaden its appeal.

We see many reflections of findings from qualitative research on white nationalism in our quantitative analysis, chief among them our discovery that the movement’s tenets are particularly attractive to young white men and people whose social lives are largely online, who qualitative work has shown are the prime recruitment targets of movement organizers^1,2,5–8,10^. A contribution to this research is our discovery that white nationalism holds greater appeal for white people of lower socioeconomic status, those facing personal hardship and those living in socially distressed areas, which stands in contrast to ethnographic work highlighting the middle-class and professional origins of many participants in the movement. Our findings also reflect qualitative work documenting how white nationalist ideas are finding more receptivity in mainstream Republican and conservative circles, and help illuminate the opportunities and limits that white nationalism faces as a right-wing social movement in US electoral politics^9,11,31,48,49^.

### Young white men

**Fig. 4:**
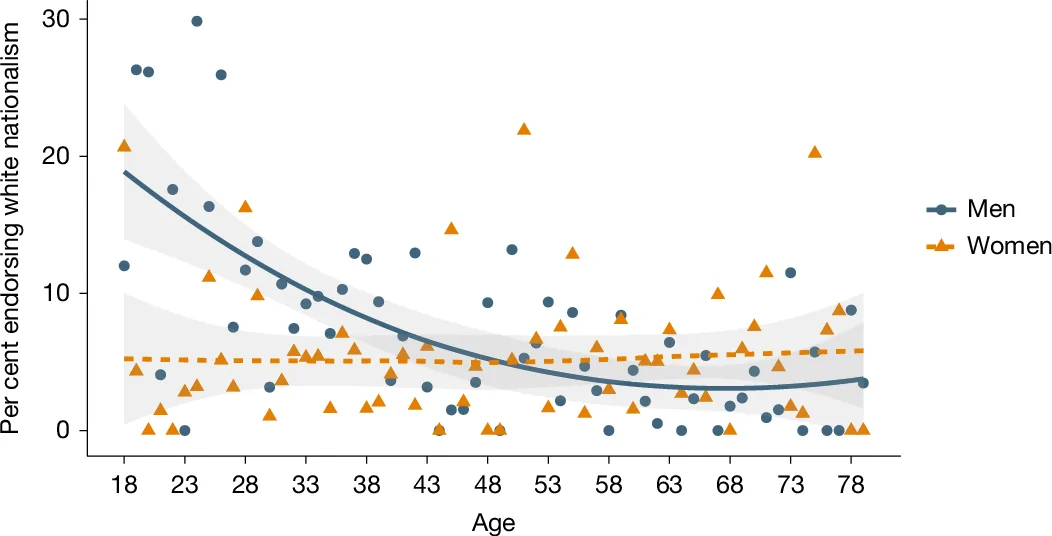
Endorsement of white nationalism among non-Hispanic white adults by age and sex. Pooled weighted prevalence of endorsement of white nationalism by age (*x* axis)—separately for men and women—in a combined sample of non-Hispanic white respondents drawn from studies 1 (CES/YouGov, 2021; *n* = 3,073) and 2 (NORC, 2024; *n* = 2,015); total *n* = 5,088. Each point is the weighted prevalence of endorsement at a single age × sex cell, computed as the sum of CES and NORC survey weights for endorsing respondents divided by the sum of weights for all respondents in that cell. The respondent-level loess smoother (span = 1) is fit separately within each sex, with each cell weighted by its unweighted cell *n*; the shaded ribbon around each curve is the loess 95% confidence band. Ages above 79 are omitted because of sparse cell sizes. An age × sex interaction in a respondent-level weighted ordinary least squares regression (endorsement ~ age × sex, pooled across studies) finds that the two trajectories differ (two-sided *P* = 0.02). Refer to Supplementary Information L for details. Source data

We find a pronounced rate of support for white nationalism among young white men, which we confirm cannot be dismissed as an artefact of acquiescence bias or inattentive response. As shown in Fig. 4, rates are highest among white men in their late teens and early twenties (Supplementary Information L). This stands in contrast to past research showing that racial animus and white identity strength are lower among younger non-Hispanic white people^23,34^. It is possible that this support for white nationalism is a life-cycle effect that fades with age: young men of every generation have long been a target for organized hate groups^50^. But high levels of support for white nationalism among young white men may also be a cohort effect that persists over time. Young men today belong to a distinctive generation in that they have fallen behind previous male cohorts on key indicators while being constantly exposed to content from the internet and social media. These two factors—diminished prospects combined with intense online activity—lay the groundwork for young men’s potential receptivity to online networks of influencers, podcasters and activists who address their challenges. In some corners of these networks, messaging is explicitly misogynist and violent^51,52^, themes that in some cases intersect with white nationalist ideology^53^.

### Racial attitudes among white people

Our examination of the relationship between support for white nationalism and established measures of white identity and racial animus discovered positive but relatively low correlations. This, alongside the fact that young white men are particularly drawn to white nationalist ideology, indicates that white nationalist support may be emerging as a distinct dimension of white peoples’ racial attitudes, as suggested by previous work^11–15^. Support for white nationalism seems to integrate two elements of racial attitudes, in-group identity and out-group resentment, yielding an expressed desire to preserve white hegemony in the US. It may represent a new dimension of racial polarization, emerging despite the current broad consensus on civil rights issues such as interracial marriage and integrated schools and neighbourhoods.

### Social strain

Support for white nationalism was stronger in areas with higher incidence of poverty, unemployment and deaths of despair, which generate environments that can catalyse grievance^3,29^. Concomitantly, support is higher among those experiencing personal hardships. Support for white nationalist ideology does not seem to be driven by local demographic change, which has long been thought to play key roles in shaping white peoples’ attitudes about race. These findings suggest that white nationalism may be met with sustained support if economic and social strains persist, regardless of local demographic contexts.

### Alienation, isolation and online lives

Support for white nationalism is predicted by alienation and social isolation, as reflected in the fact that the movement’s ideas are particularly appealing to white people whose primary friendships are mostly—or entirely—online. At the same time, more frequent use of social media was not correlated with increased support for white nationalist ideas. This suggests that online activity may be especially consequential for people who are socially isolated offline, potentially driving these people to feel pressured to adopt views aligned with their online communities. Given the tactics of online mobilization practiced by extremist political movements^26,27^, recruitment may be facilitated not through digital engagement alone, but in conjunction with offline social dynamics. In this framework, social alienation combined with intensive online activity fosters the adoption of more hard-line views. But given the limitations of our cross-sectional data, we cannot rule out the possibility that the causal process runs in the opposite direction. Strongly held, unconventional political views may make it more difficult for people to form close friendships offline, increasing their reliance on online communities of like-minded others. If either or both of these dynamics is operating here, they may help explain not only the online spread of white nationalism, but other extremist views across the political spectrum as well.

### Future research

The dynamic nature and rising salience of white nationalism in the US suggest that our results be considered a snapshot in time rather than predictive about the direction of future trends, which should be assessed with repeated survey items fielded to nationally representative samples. Inferential leverage could be gained by conducting longitudinal studies, particularly among young people, to examine how and why views change over time. Our short description of white nationalism focuses on purported demographic threats, claims about superiority of white culture, and the maintenance of white political power as the three main tenets of the movement. Further research might explore the relative attraction of each of these ideas (as well as others) in explaining white nationalism’s appeal, and the relationship between support for white nationalist views as measured here and specific tactics and goals of the white nationalist movement in the US.

## Methods

### Study 1 (survey fielded in 2021; YouGov CES)

We fielded this extensive survey to *N* = 3,227 non-Hispanic white people in November 2021 as part of the CES—a widely used source of data in peer-reviewed political science research on US public opinion. The CES’s in-depth Common Content questionnaire and respondent geocodes permit documentation of the demographic and political predictors of support for white nationalism and exploration of our theories. The CES was conducted on the web by the YouGov survey firm; respondents were selected from an opt-in panel using a sample matching and weighting methodology that yields estimates aiming to approximate those obtained from nationally representative probability samples of US adults^37^. This approach assumes that—conditional on the demographic variables used for weighting—panel participation is independent of the attitudes measured here. We note that this assumption cannot be directly verified, particularly for stigmatized political opinions and identities.

The CES is made up of modules with original questionnaires developed by teams of university-based researchers fielded to 1,000 respondents each. All respondents also respond to the CES Common Content questionnaire, which includes items about demographics, partisanship and vote choice, policy preferences, political knowledge and related topics.

We contracted with the CES to purchase questionnaire time for our study on five modules, which yielded a total of 5,000 respondents. Our questionnaire was fielded only to respondents who indicated they were white on the Common Content item about racial identity—a question that also included response choices of ‘Black’, ‘Hispanic’, ‘Asian’, ‘Native American’, ‘Two or more races’, ‘Other’ and ‘Middle Eastern’. We further restrict our analysis to white people who answered ‘no’ to a subsequent CES question asking all those who did not indicate ‘Hispanic’ on the race item whether they identified as Hispanic or Latino. Our resulting sample (*N* = 3,227) aims to be representative of the US non-Hispanic white adult population, estimated by the Census Bureau at 161 million in 2021 (62.1% of all adults). Demographic, political and other relevant characteristics of our sample can be found in Supplementary Information B.

The entire questionnaire for study 1 may be found in Supplementary Information A. Here we provide sources and wording of key survey items discussed in the text.

#### Strength of white identity and support for white collective action

Adapted from items employed in previous work^23,24^. Items presented in random order.

Please indicate how much you agree or disagree with the following statements:

- Being white is important to my identity.
- White people in this country have a lot to be proud of.
- Whites in this country have a lot in common with each other.
- Whites need to do more to remind the world about the challenges that white people face.
- Whites need to start looking out more for one another.
- We need a political party focused on advancing white power.

Items accompanied by a five-point scale ranging from ‘strongly disagree’ to ‘strongly agree’.

#### Group power

Items presented in random order.

Please say whether you think the following groups have too little, too much or the right amount of power in the US today.

- Whites
- Blacks
- Asians
- Hispanics
- Evangelical Christians
- Jews
- Muslims

Items accompanied by a three-point scale ‘too little power’, ‘the right amount of power’ and ‘too much power’.

#### Intimidation, harassment and violence against non-white people

Adapted from items developed in previous work measuring partisan political polarization^55^.

Please indicate your stance on the following questions.

- When, if ever, is it OK for whites to send threatening and intimidating messages to non-white leaders?
- When, if ever, is it OK for an ordinary white person in the public to harass an ordinary non-white person on the internet, in a way that makes that person feel unsafe?
- How often do you feel it is justified for whites to use violence in advancing their political goals these days?

Items accompanied by the four-point response set ‘never’, ‘occasionally’, ‘frequently’ and ‘always’.

#### Endorsement of white nationalist ideology

Respondents were randomly assigned in equal numbers to ‘treatment’ and ‘control’ groups. The treatment group was given the following description of the white nationalist movement: “The white nationalist movement holds that whites are under threat in the United States and seeks to ensure the survival of the white race in this country. White nationalists believe that white people should hold more political and economic power than other groups. They believe that whites should maintain their majority in the US and that white culture is superior to all other cultures”. These respondents were then asked, “Are you a supporter of the white nationalist movement?” and were instructed to indicate ‘yes’ or ‘no’ on their screen.

The control group was first asked this question, their responses recorded, and then asked the question again after reading the short description of white nationalism. Except where noted, throughout this paper we combine the fully informed responses from the control group with responses from the treatment group when reporting support for white nationalism in our study.

### Study 2 (survey fielded in 2024; NORC AmeriSpeak)

We replicated our key results with a brief nationally representative survey of *N* = 2,114 non-Hispanic white people conducted in May and June 2024 via NORC’s AmeriSpeak panel^38^. AmeriSpeak randomly selects households from a national sampling frame using area probability and address-based sampling. Sampled households are recruited into the panel by US mail, telephone and field interviewers. Most respondents participated by web, but non-internet households (3.3% of the sample) participated by telephone.

This survey consisted only of our measure of white nationalist support, with respondents randomly assigned to treatment and control groups via the same wording and experimental procedure as described above. Our analyses make use of the demographic and political profile information NORC collected on AmeriSpeak panelists. The entire questionnaire for study 2 may be found in Supplementary Information A; demographic, political and other relevant characteristics of our sample can be found in Supplementary Information B.

### Study 3 (survey fielded in 2024–2025; CloudResearch)

In late 2024 and early 2025 we fielded a supplementary survey to a national quota-based sample of *N* = 2,548 non-Hispanic white people recruited by CloudResearch^39^. We stipulated quotas regarding respondents’ sex, age and education corresponding to their American Community Survey estimates for the US non-Hispanic white adult population, as well as a quota for partisanship as estimated by the 2024 CES. The entire questionnaire for this survey may be found in Supplementary Information A; demographic, political and other relevant characteristics of our sample can be found in Supplementary Information B.

We fielded this survey using the Qualtrics platform. The study began with a series of demographic and political questions. Respondents were then asked batteries of items (presented in random order) about (1) white identity^23^; (2) holding of conspiracy beliefs^56^; (3) denial of white privilege^57^; (4) racial resentment^58^; (5) belief in the ‘great replacement’ theory^59^; and (6) ‘old-fashioned racism’^60^. As shown in Supplementary Information B, question wording largely followed that used in past research on racial attitudes. We reverse-keyed some battery items in order to address concerns about acquiescence bias.

#### Experimental design

We probed vulnerabilities of our white nationalism measure to survey response bias with survey experiments in which participants were randomly assigned to conditions as reported in Extended Data Table 5. All participants received the short description of the white nationalist movement described in the text. This was followed by variations on our original item measuring support for the movement. Participants assigned to Condition A were asked “Are you a [supporter/opponent] of the white nationalist movement?” with half of these respondents assigned to the ‘supporter’ wording (that is, the question we fielded in studies 1 and 2) and half assigned to the ‘opponent’ wording. Participants assigned to Condition B were asked “Do you [oppose or support/support or oppose] the white nationalist movement?” with half assigned to each wording. To assess priming effects, half of our participants answered their assigned version of the white nationalism item at the beginning of the survey (before responding to any racial attitudes batteries); half answered the white nationalism item at the very end of the survey.

### Correlations with measures of racial attitudes

Our measure of support for white nationalism bears some similarities with other established measures of racial attitudes developed by scholars in political science, sociology and psychology^23,57–60^. We found our measure to be most strongly empirically correlated with two of these established constructs (in study 3; see Extended Data Table 4 and Supplementary Information D). Our measure’s specification that white nationalists believe white people should “hold more political and economic power than other groups” and that “white culture is superior to all other cultures” finds common ground with elements of the white identity battery^23^ discussed above (Pearson’s *r* = 0.40 with our measure). Our measure’s inclusion of the ideas that white nationalists attest that white people are “under threat” and should “maintain their majority in the US” sounds some themes similar to those in “great replacement theory”^59^ about immigration (*r* = 0.31). We found weaker, but still positive, relationships between white nationalism and ‘old-fashioned racism’^60^ (*r* = 0.26); racial resentment^58^ (*r* = 0.25); and denial of white privilege^57^ (*r* = 0.15), suggesting that we are measuring a phenomenon adjacent to, but crucially distinct from, these established constructs.

### Investigating upward bias

Estimates of low propensity attitudes can be artificially inflated by a number of factors. We explored the vulnerability of our prevalence estimates to upward bias by (1) the responses of inattentive participants, (2) acquiescence bias and (3) priming effects.

Inattentive respondents who answer questions in a random fashion can inflate estimates of low-propensity beliefs^40^. The vendors with which we contracted to administer studies 1, 2 and 3 all used a number of techniques to identify and remove inattentive respondents from their samples. To further address this concern, we re-analysed data from study 1 to determine the ceteris paribus relationship between the amount of time respondents took to complete the survey (which previous work has shown is correlated with attentiveness^41,61^) and support for white nationalism after controlling for the predictors described in this article. The analysis yielded estimates robust to inattentiveness that were only slightly lower (Supplementary Information E). The brevity of our survey in study 2 prohibited conducting a response-time analysis with those data. In study 3, no relationship was found between response time and support for white nationalism (Supplementary Information E).

We also investigated whether our yes–no survey item about supporting the white nationalist movement could be subject to acquiescence bias—that is, the tendency to agree with statements in surveys regardless of actual beliefs. Study 3 featured an experiment recommended by political methodologists^62^, in which, after being provided with the description of white nationalist beliefs, half of respondents were randomly assigned our original question (“Are you a supporter of the white nationalist movement?”) and the other half were asked a version keyed in the opposite direction (“Are you an opponent of the white nationalist movement?”). This experiment found no evidence that prevalence estimates from our measure are inflated by acquiescence bias. Further analyses suggest that results from study 3 regarding acquiescence bias are likely to generalize to other samples regardless of their respondents’ level of attentiveness (Supplementary Information F).

A final concern is that prevalence estimates from study 1 may have been biased upward by priming effects^63^, because in this study our measure of white nationalist support was placed at the end of a survey that contained an extensive series of questions about racial attitudes. Study 3 investigated this concern with a question-order experiment in which half of participants were randomly assigned to answer a question about their support for white nationalism before responding to any of the other items in the survey about racial attitudes. The other half was assigned to do this at the conclusion of the survey, which included several batteries of items related to racial attitudes, including white identity, racial resentment and ‘old-fashioned racism’. As shown in Supplementary Information F, this experiment found no evidence that priming effects inflated prevalence estimates.

### Insignificant results and evidence in support of the null

In this work we report several statistical tests that yield insignificant estimates: they cannot reject the null hypothesis that the association between a predictor (or an experimental treatment) and support for white nationalism is zero. As failure to reject the null does not necessarily imply evidence in support of the null itself, we further characterize these findings to ascertain whether such evidence is present. We do this in one of two ways, depending on the nature of the statistical test.

Where a single predictor—an experimental treatment indicator or regression coefficient—of support for white nationalism is found to be statistically insignificant, we conduct an equivalence test to determine whether the coefficient is small enough in magnitude to be substantively negligible^64^. We define a treatment effect or ceteris paribus association of 2.5 pp as our benchmark of substantively meaningful magnitude. This threshold was not preregistered, but was set before conducting the equivalence tests. It was chosen for consistency with the approximately 2.5 pp minimal detectable effect (at 80% power) in the experiment conducted in study 2. By this specification, ceteris paribus associations falling within the range of −2.5 to +2.5 pp are considered negligible. Our equivalence tests use two one-sided tests to first determine whether the true association is greater than the lower bound of −2.5 pp and then to determine whether the association is lower than the upper bound of +2.5 pp. Only when both one-sided tests reject their respective null hypotheses do we conclude that the estimated association is statistically equivalent to zero within our prespecified margin, and the equivalence test has passed. Otherwise, we conclude that the data do not provide sufficient evidence to establish this equivalence, and the equivalence test has failed. We report the outcome of each equivalence test and its corresponding interpretation.

Where a categorical predictor of support for white nationalism is found to be statistically insignificant in a multiple regression model, we conduct a Bayesian information criterion (BIC)-based model comparison to determine whether including this predictor meaningfully improves the fit of the model^65^. We calculate the difference between the BIC statistic of the alternative model that includes the predictor (BIC_A_) and that of the null model (BIC_0_) and report ΔBIC ≡ BIC_A_ – BIC_0_. For each test, we report ΔBIC and the corresponding strength of evidence in favour of the null model, adopting conventional standards in which ΔBIC of greater than about 2, 6 and 10 correspond to (respectively) roughly positive, strong and very strong Bayes-factor evidence in favour of the null model.

### Ethics statement

The study protocols were approved by the NYU Institutional Review Board. Study 1 was approved under exempt review on 20 August 2021 (IRB-FY2021-4955). Study 2 was approved under an expedited review on 8 May 2024; study 3 was approved with a modification to this protocol under an expedited review on 4 November 2024 (IRB-FY2024-8846). Research was performed in accordance with relevant guidelines and regulations. Informed consent was obtained from all participants.

### Reporting summary

Further information on research design is available in the Nature Portfolio Reporting Summary linked to this article.

## Online content

Any methods, additional references, Nature Portfolio reporting summaries, source data, extended data, supplementary information, acknowledgements, peer review information; details of author contributions and competing interests; and statements of data and code availability are available at https://doi.org/10.1038/s41586-026-11018-0.

## Supplementary information

Supplementary Information Supplementary Information Sections A–L, including Supplementary Figs. 1–6, Tables 1–28 and references.

Reporting Summary

## Source data

Source Data Fig. 1

Source Data Fig. 2

Source Data Fig. 3

Source Data Fig. 4

Source Data Extended Data Fig. 1

## Figures and Tables

**Extended Data Table 1:**
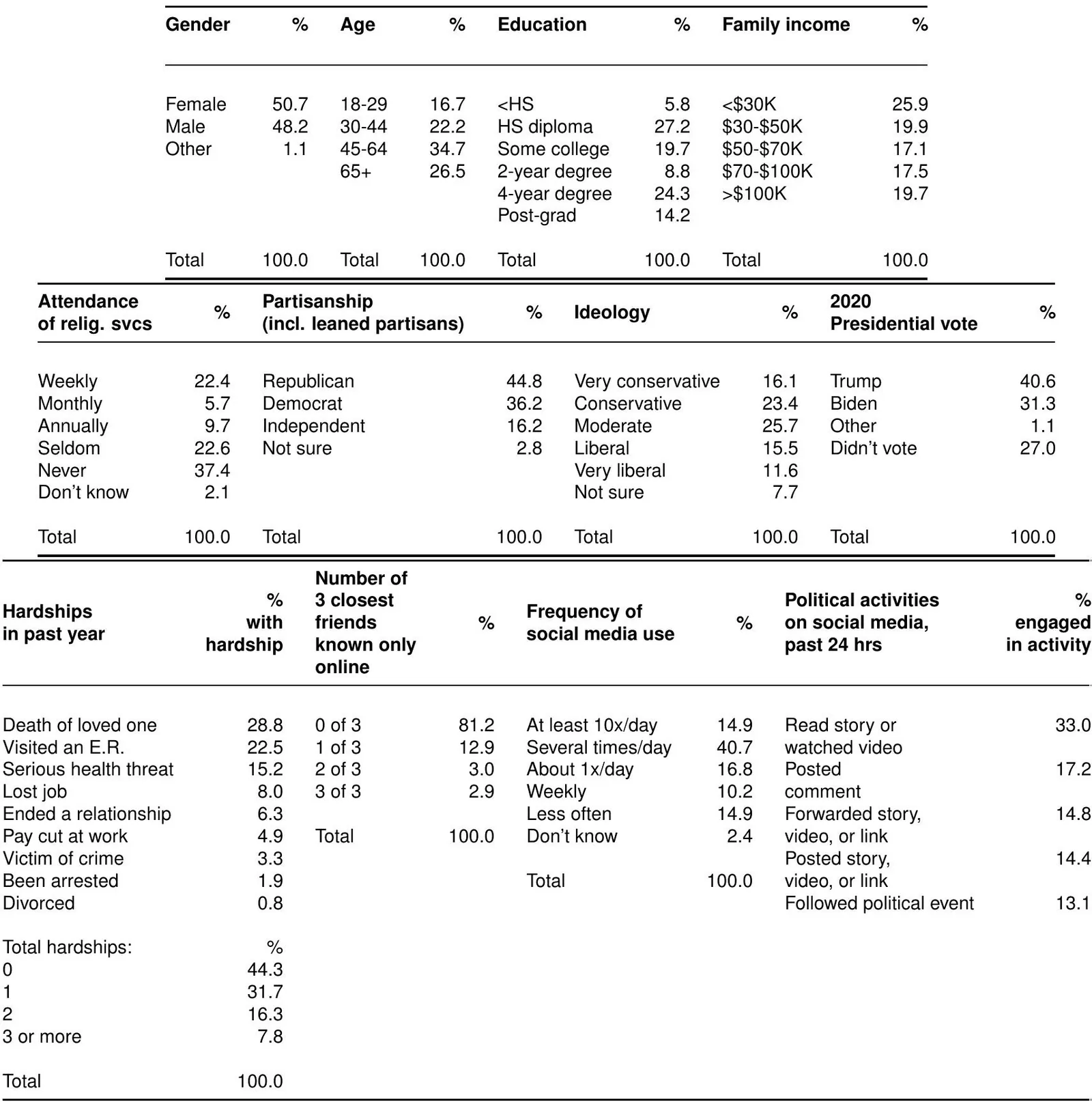
Summary statistics for Study 1

**Extended Data Table 2:**
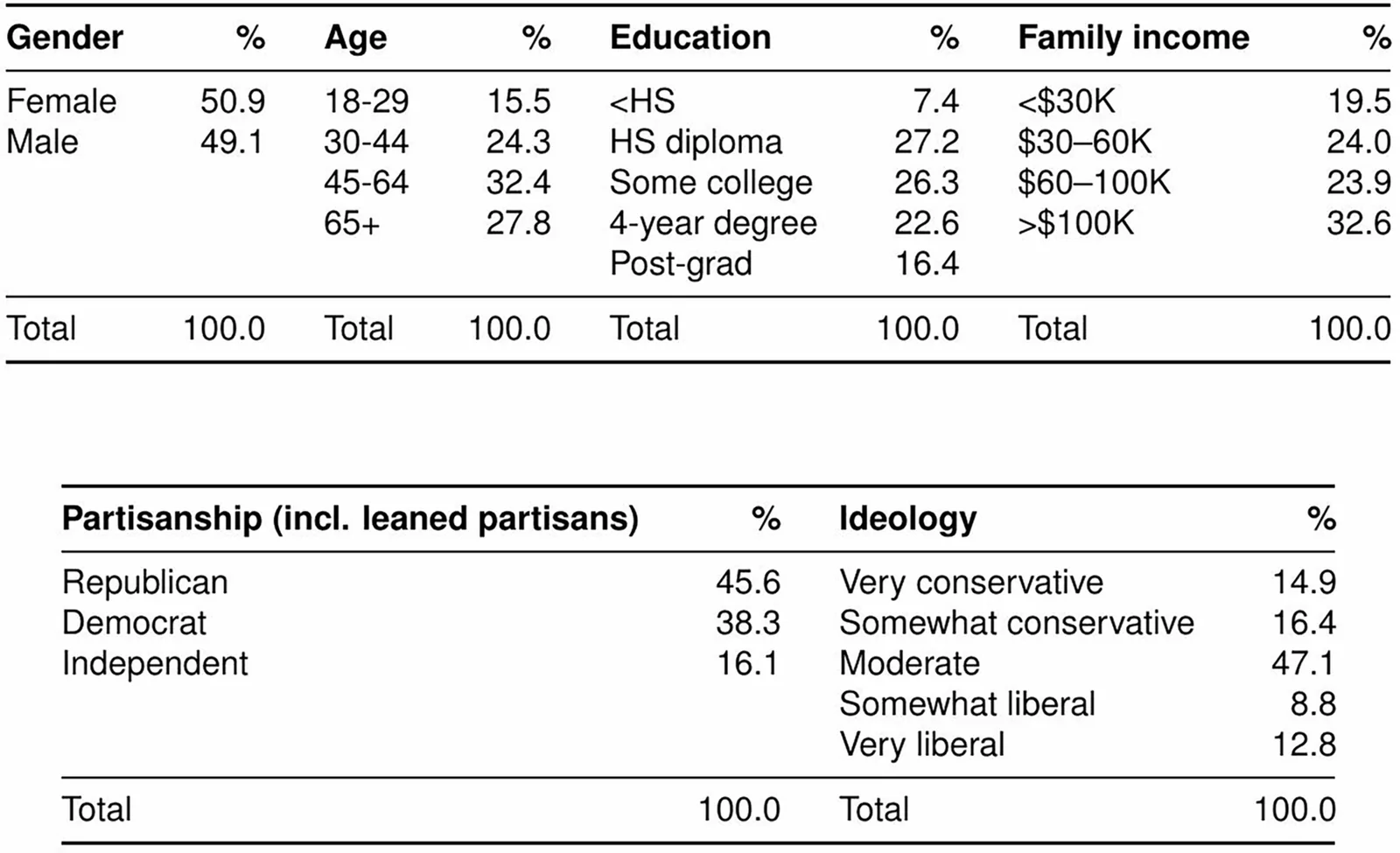
Summary statistics for Study 2

**Extended Data Table 3:**
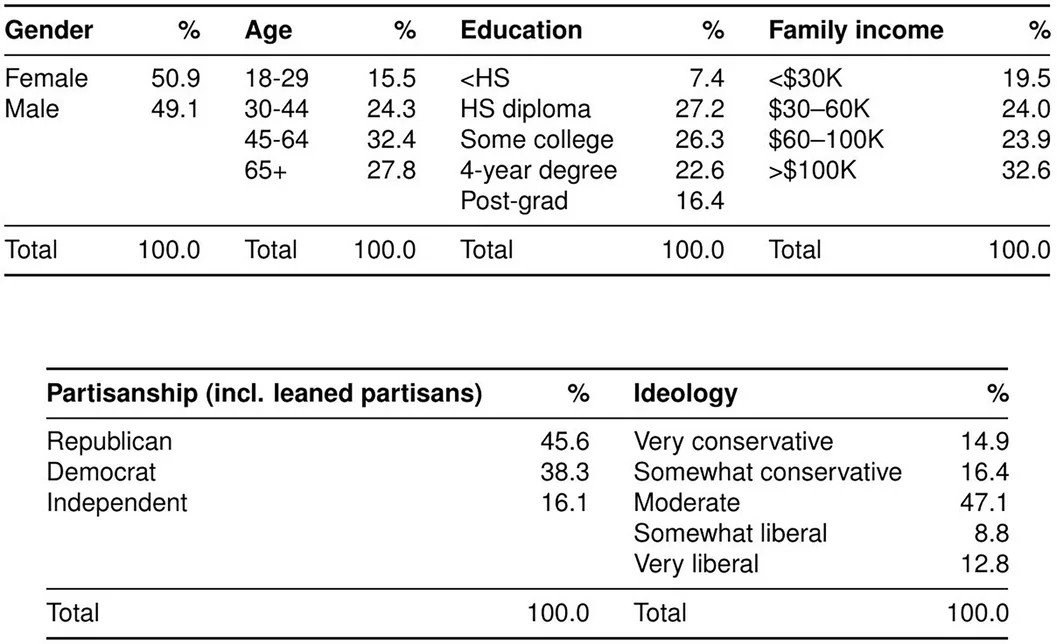
Summary statistics for Study 3

**Extended Data Table 4:**
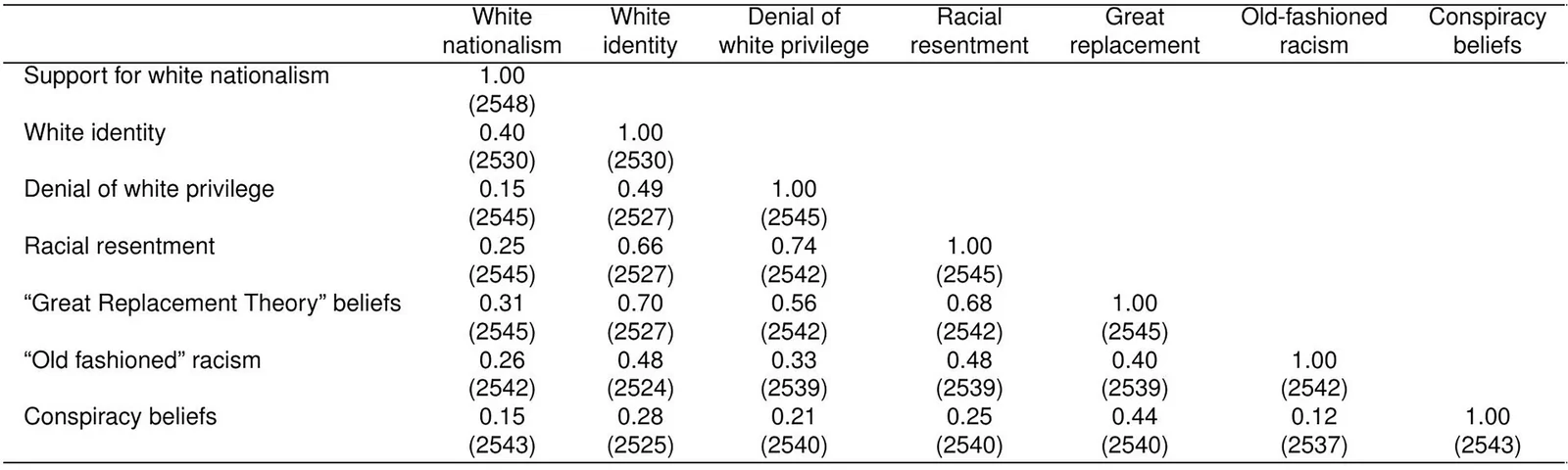
Correlations between support for white nationalism and other measures of racial attitudes and conspiracy beliefs, Study 3

Cells report pairwise correlations and valid *N*. See Supplementary Information section A for details of measures.

**Extended Data Table 5:**
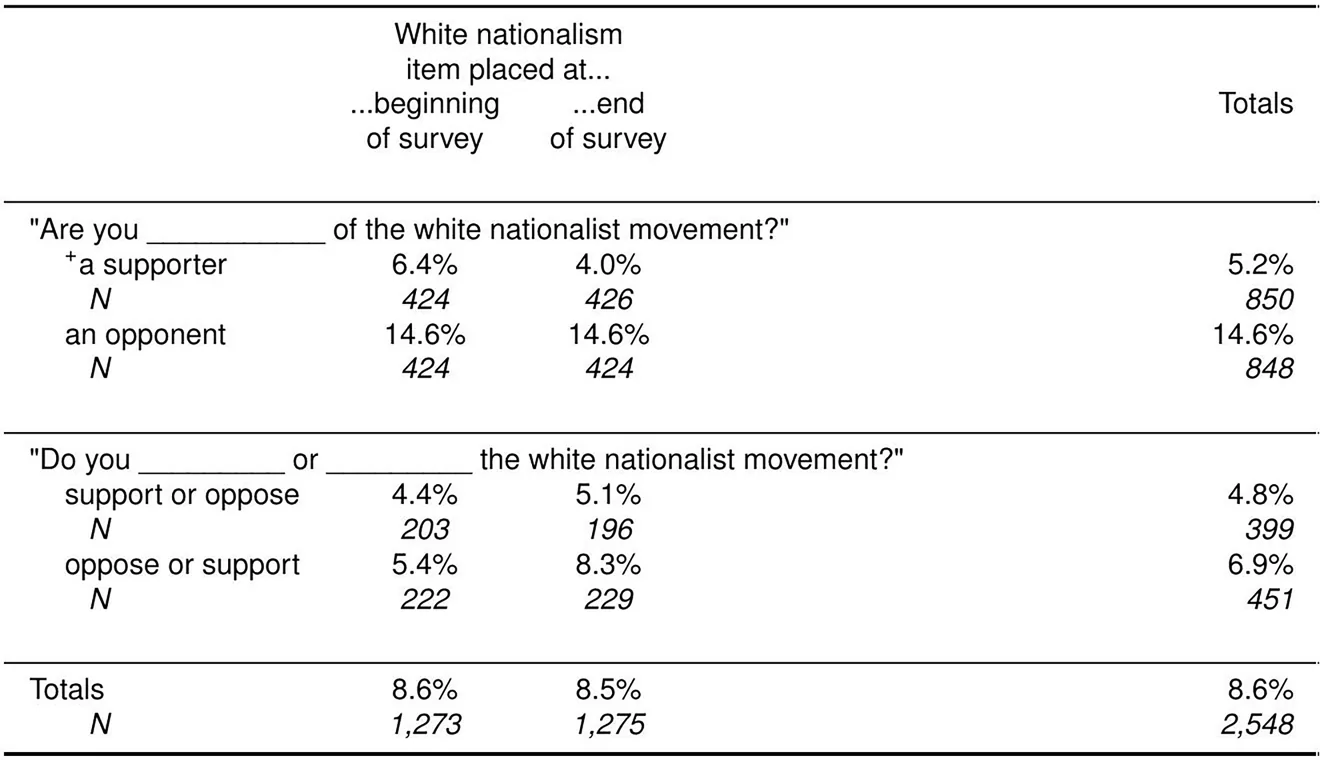
Experimental conditions and expressed support for the white nationalist movement, Study 3

Entries are percentages of respondents expressing support for the white nationalist movement in each treatment condition and number of respondents in each condition. See Supplementary Information section F for details and test results. +This version is identical to the white nationalism item fielded in Study 1 and Study 2.

**Extended Data Table 6:**
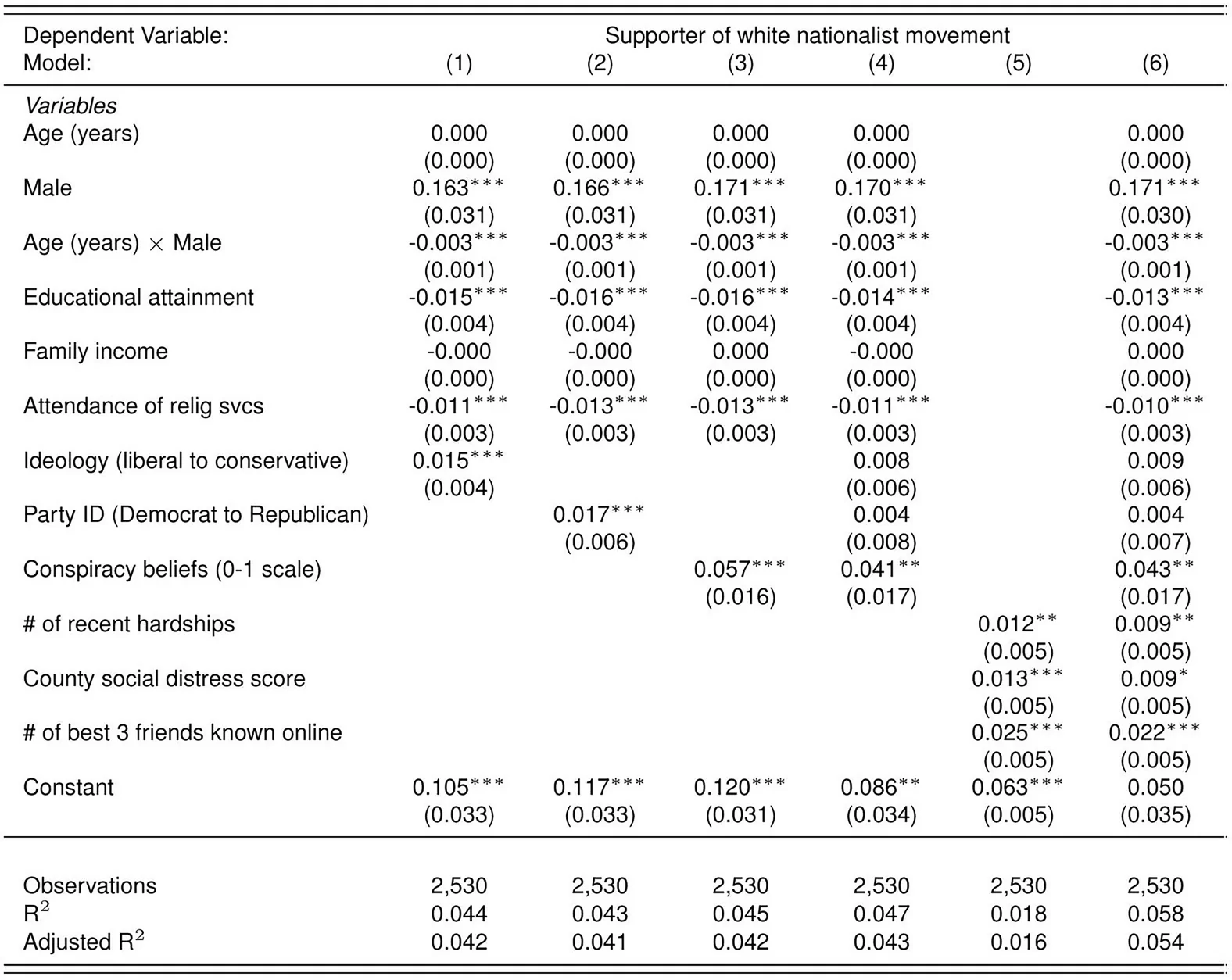
Robustness of associations between predictors and support for white nationalism considered ceteris paribus, Study 1

Weighted OLS regression estimates of ceteris paribus associations between demographic and political covariates and support for white nationalism. Models 1–3 include all demographic predictors and—due to their anticipated collinearity—each of the three political predictors entered separately. All political predictors are included together in Model 4. Model 5 jointly tests personal hardship, local social distress, and online-only friendships as predictors of white nationalist support. Model 6 includes demographics, political views, and the three theoretically motivated predictors found to be statistically significant predictors in bivariate tests displayed in Fig. 3. ***, **, and * indicate statistical significance at the 1%, 5%, and 10% level, respectively (all tests are two-sided). See Supplementary Information section G for details.

**Extended Data Table 7:**
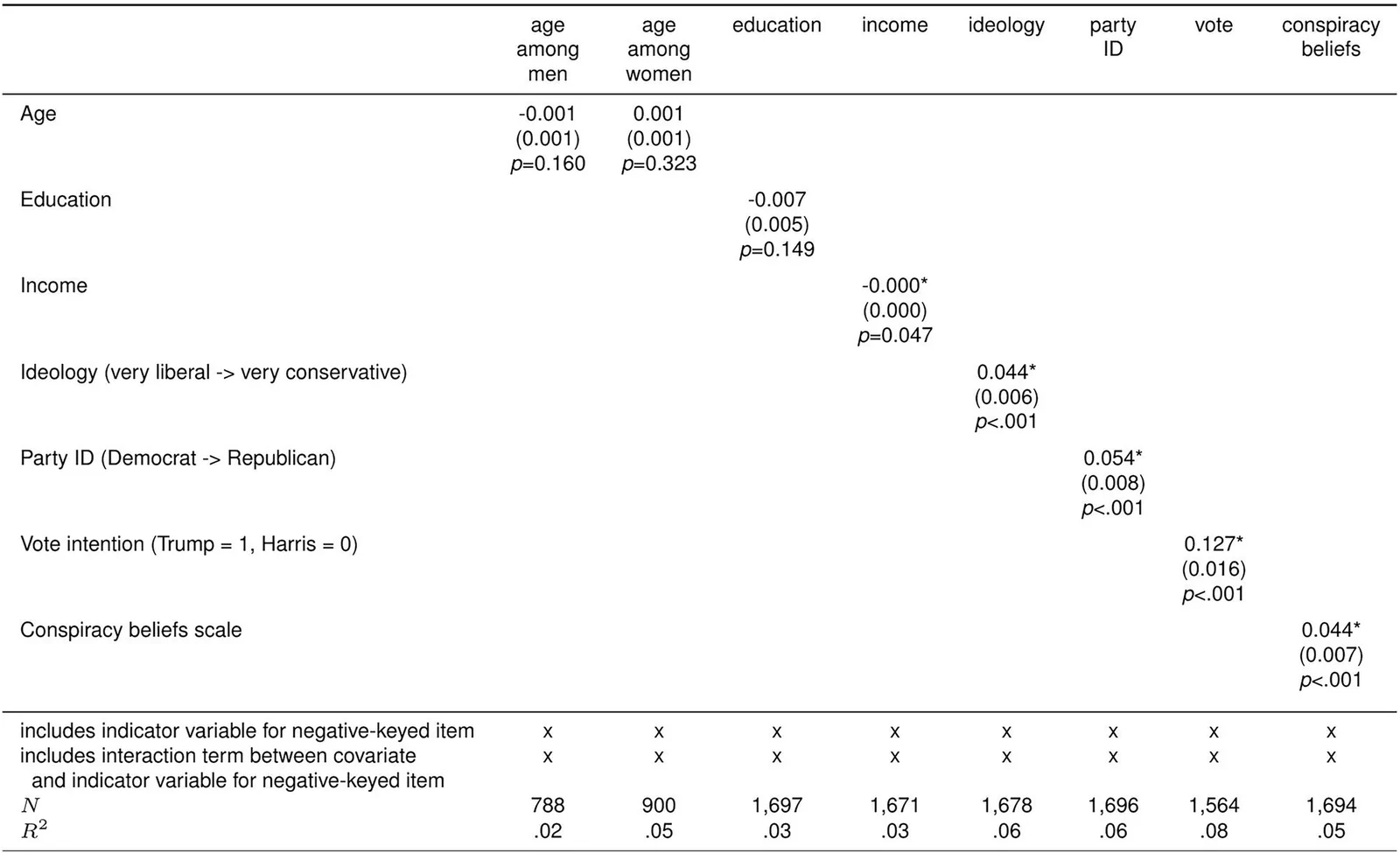
Predictors of support for white nationalism corrected for acquiescence bias, Study 3

Analyses of survey experiment in Study 3 where respondents were randomly assigned to either a positive-keyed item (“Are you a SUPPORTER of the white nationalist movement?”) or negative-keyed item (“Are you an OPPONENT of the white nationalist movement?”). An indicator variable was generated for each respondent, scored 1 when the item was positive-keyed and −1 when negative-keyed. For each of the covariates listed in the table columns, a separate regression was conducted in which the white nationalism measure was regressed on the covariate, the reverse-keying indicator, and their interaction. Displayed are the covariate coefficients, which by construction are estimates of the relationships between the predictor and white nationalist support net of acquiescence bias. * indicates statistical significance at the 5% level (all tests are two-sided). See Supplementary Information section G for details.

## Data Availability

The replication data are available at P.J.E.’s Harvard Dataverse repository page at https://doi.org/10.7910/DVN/J6G51T. The repository contains survey data, contextual data, and all code necessary for replication. Source data are provided with this paper.

## Code availability

This study uses the R open-source statistical software program (v.4.6.1)^66^ for all data analysis and data visualizations. Details on the code used are provided at P.J.E.’s Harvard Dataverse repository page, which can be accessed via https://doi.org/10.7910/DVN/J6G51T.
