# Supplementary material for: Predictors and prevalence of support for white nationalism in the United States
Source: Nature. 2026 Sep 16;657(8133):964–72. doi: 10.1038/s41586-026-11018-0 (PMC13600921; doi:10.1038/s41586-026-11018-0)
Supplement: Supplementary file 1 [file 41586_2026_11018_MOESM1_ESM.pdf]

nature portfolio

https://doi.org/10.1038/s41586-026-11018-0

Supplementary information

Predictors and prevalence of support for white nationalism in the United States

In the format provided by the authors and unedited

Supplementary Information for

Predictors and prevalence of support for white nationalism in the United States

Patrick J. Egan, James Bisbee and Joshua A. Tucker

Address correspondence to:
Patrick J. Egan (patrick.egan@nyu.edu)

This PDF file includes:

Supplementary Information
Figs. S1 to S6
Tables S1 to S28
SI References

Supplementary Information Guide

Sections

Section A: Full text of survey questionnaires (Studies 1, 2, and 3)

Section B: Descriptive statistics (Studies 1, 2 and 3)

Section C: Details of tests validating our measure of support for white nationalist views (Study 1)

Section D: Correlations between our measure of support for white nationalism and other measures of racial attitudes and conspiracy beliefs (Study 3)

Section E: Robustness of estimates to respondent inattentiveness (Studies 1 and 3)

Section F: Details of survey experiments assessing acquiescence bias and priming effects (Study 3)

Section G: Estimation of relationship between support for white nationalism and demographic and political covariates (Studies 1 and 2); robustness of relationships to acquiescence bias (Study 3)

Section H: Robustness checks and sensitivity analysis (Study 1)

Section I: Assessing evidence for strain, status threat, and online mobilization theories (Study 1)

Section J: Occupational classification and measures of occupational insecurity (Study 1)

Section K: Sources and generation of county-level data (Study 1)

Section L: Estimates giving rise to age- and sex-specific trends in support for white nationalism in Figure 4 (Studies 1 and 2)

SI section A:
Full text of survey questionnaires (Studies 1, 2, and 3)

Survey question wording - Study 1. Our survey questionnaire for the 2021 CES module appears on the following pages. All of our participants also responded to the 2021 CES Common Content (1). The full Common Content questionnaire may be found at the CES public data repository at the Harvard Dataverse: https://doi.org/10.7910/DVN/OPQOCU.

NYU Center for Social Media and Politics (CSMAP)
Half Module for 2021 CES

This version: October 2021

IMPORTANT NOTE FOR PROGRAMMERS:

Questions SMP101 through SMP119 are designed only to be fielded with white CES respondents.
Questions SMP101 through SMP119 should be skipped by respondents who do not identify as white.

Respondents should only respond to questions SMP101 through SMP119 if *race* = 1 in their profile data. All other respondents should skip these questions.

NYU CES MODULE QUESTIONNAIRE -1

IF *implicit_page_employ* equals either “Working full time now” or “Working part time now”,
display SMP001 and SMP002 and skip SMP003 and SMP004

ELSE
Skip SMP001 and SMP002 and display SMP003 and SMP004

Instructions for programmers

SMP001, SMP002
Variable Names

SHORT TEXT BOX
Variable Type

Work – current
Workplace - current
Variable Labels

Thinking about your current job, what kind of work do you do?
Question Text

SMP001 TEXT BOX

And what kind of place do you currently work for?
Question Text

SMP002 TEXT BOX

SMP003, SMP004
Variable Names

SHORT TEXT BOX
Variable Type

Work – last
Workplace - last
Variable Labels

Thinking about your last job, what kind of work did you normally do there?
Question Text

SMP003 TEXT BOX

What kind of place did you work for?
Question Text

SMP004 TEXT BOX

SMP005 through SMP009
Variable Names

MULTIPLE CHOICE
Variable Type

Recent – breakup
Recent – death
Recent – arrest
Recent – health
Recent - none
Variable Labels

Now we'd like to ask you a few more questions about things that may have happened in your life recently.

Over the past year, have you… (Check all that apply)
Question Text

SMP005	…ended a relationship?
SMP006	…experienced the death of a loved one or family member?
SMP007	…been arrested?
SMP008	…experienced a serious threat to your health?
SMP009	None of the above

SMP010
Variable Names

SINGLE CHOICE
Variable Type

Social Media Use
Variable Label

How often do you use online social media?
Question Text

1 – At least 10 times a day
2 – Several times a day
3 – About once a day
4 – 3 to 6 days a week
5 – 1 to 2 days a week
6 – Every few weeks
7 – Less often
8 – Don’t know

SMP011 through SMP024
Variable Names

MULTIPLE CHOICE
Variable Type

Social Media Use -	Bitchute
Social Media Use -	Discord
Social Media Use -	Facebook
Social Media Use -	Gab
Social Media Use -	Instagram
Social Media Use -	Parler
Social Media Use -	Reddit
Social Media Use -	Snapchat
Social Media Use -	Telegram
Social Media Use -	Twitch
Social Media Use -	Twitter
Social Media Use -	WhatsApp
Social Media Use -	YouTube
Social Media Use -	None

Variable Labels

Do you have accounts on any of the following platforms or apps? (Check all that apply)
Question Text

SMP011	Bitchute
SMP012	Discord
SMP013	Facebook
SMP014	Gab
SMP015	Instagram
SMP016	Parler
SMP017	Reddit
SMP018	Snapchat
SMP019	Telegram
SMP020	Twitch
SMP021	Twitter
SMP022	WhatsApp
SMP023	YouTube
SMP024	None of the above

SMP025 through SMP028
Variable Names

MULTIPLE CHOICE
Variable Type

Social Media New - Bitchute
Social Media New - Gab
Social Media New- Parler
Social Media New - Telegram
Variable Labels

Please only display platforms on which respondent indicated having an account:
If SMP011 = Yes, display SMP025
If SMP014 = Yes, display SMP026
If SMP016 = Yes, display SMP027
If SMP019 = Yes, display SMP028

Instructions for programmers

Have you **started using** any of the following platforms **in the past year**? (Check all that apply)
Question Text

SMP025	Bitchute
SMP026	Gab
SMP027	Parler
SMP028	Telegram

SMP029 SMP030 SMP031
Variable Names

GRID
Variable Type

How Know - Friend 1
How Know - Friend 2
How Know - Friend 3
Variable Labels

Please think of your three closest friends -- not counting your spouse, partner, or relatives -- and put the initial of their first name on this list.

For each friend, please say whether you know them **only from online interactions**, **only from offline interactions**, or if you interact with your friend **both online and offline**.
Question Text

ROWS [PLEASE MAKE THE TEXT BOXES SHORT – ENOUGH TO FIT JUST ONE LETTER]

SMP029 ___ is somebody I know…
SMP030 ___ is somebody I know…
SMP031 ___ is somebody I know…

COLUMNS
HEADER CENTERED ABOVE THE COLUMNS: I interact with this friend…
1 – …only online
2 – …only offline
3 – …both online and offline

SMP032-SMP034
Variable Names

GRID
Variable Type

Conspiracy – Run the country
Conspiracy – Small groups
Conspiracy - Plots
Variable Labels

Please <u>randomize</u> the order of the rows.
Instructions for programmers

Please indicate how much you agree or disagree with the following statements.
Question Text

ROWS

SMP032	The people who really "run" the country are not known to the voters.
SMP033	Big events like wars, the recent recession, and the outcomes of elections are controlled by small groups of people who are working in secret against the rest of us.
SMP034	Much of our lives are being controlled by plots hatched in secret places.

COLUMNS
1 - Strongly disagree
2 - Somewhat disagree
3 - Neither agree nor disagree
4 - Somewhat agree
5 - Strongly agree

IMPORTANT NOTE FOR PROGRAMMERS
(REPEATING INSTRUCTIONS AT BEGINNING OF THIS DOCUMENT)

Questions SMP101 through SMP119 are designed only to be fielded with white CES respondents.
Questions SMP101 through SMP119 should be skipped by respondents who do not identify as white.

Respondents should only respond to questions SMP101 through SMP119 if *race* = 1 in their profile data. All other respondents should skip these questions.

SMP101 through SMP106
Variable Names

GRID
Variable Type

White Identity – Important
White Identity – Proud
White Identity – In Common
White Identity – Remind
White Identity – Looking Out
White Identity – Political Party
Variable Labels

Ask only if *race* = 1
Please <u>randomize</u> the order of the rows.
Please keep the items together in a grid instead of asking them separately.
Instructions for programmers

Please indicate how much you agree or disagree with the following statements.
Question Text

ROWS

SMP101	Being white is important to my identity.
SMP102	White people in this country have a lot to be proud of.
SMP103	Whites in this country have a lot in common with each other.
SMP104	Whites need to do more to remind the world about the challenges that white people face.
SMP105	Whites need to start looking out more for one another.
SMP106	We need a political party focused on advancing white power.

COLUMNS
1 - Strongly disagree
2 - Somewhat disagree
3 - Neither agree nor disagree
4 - Somewhat agree
5 - Strongly agree

SMP107 through SMP113
Variable Names

GRID
Variable Type

Power-Whites
Power-Blacks
Power-Asians
Power-Hispanics
Power-Christians
Power-Jews
Power-Muslims
Variable Labels

Ask only if *race* = 1
Please randomize the order of the rows.
Please **bold** column text as indicated.
Please keep the items together in a grid instead of asking them separately.
Instructions for programmers

Please say whether you think the following groups have too little, too much, or the right amount of power in the U.S. today.
Question Text

ROWS

SMP107	Whites have…
SMP108	Blacks have…
SMP109	Asians have…
SMP110	Hispanics have…
SMP111	Evangelical Christians have…
SMP112	Jews have…
SMP113	Muslims have…

COLUMNS
1 - **Too little** power
2 - The **right amount** of power
3 - **Too much** power

SMP114 through SMP116
Variable Names

GRID
Variable Type

OK – Threats
OK – Harass
OK – Violence
Variable Labels

Ask only if *race* = 1
Please keep the items together in a grid instead of asking them separately.
Instructions for programmers

Please indicate your stance on the following questions.
Question Text

ROWS

SMP114	When, if ever, is it OK for whites to send threatening and intimidating messages to non-white leaders?
SMP115	When, if ever, is it OK for an ordinary white person in the public to harass an ordinary non-white person on the internet, in a way that makes that person feel unsafe?
SMP116	How often do you feel it is justified for whites to use violence in advancing their political goals these days?

COLUMNS
1 - Never
2 - Occasionally
3 - Frequently
4 - Always

Please randomly assign all respondents for whom ***race = 1*** evenly into two groups.

Group 1 should receive SMP117 and then SMP118. All Group 1 members skip SMP119.

Group 2 should skip SMP117 and SMP118 and receive SMP119.
Instructions for programmers

NYU CES MODULE QUESTIONNAIRE -15

SMP117
Variable Names

SINGLE CHOICE
Variable Type

White nationalist - short
Variable Label

Are you a supporter of the white nationalist movement?
Question Text

Ask only if *race* = 1 and respondent is assigned to Group 1
Instructions for programmers

COLUMNS
1 - Yes
2 - No

SMP118
SINGLE CHOICE
Variable Type

White nationalist - confirm
Variable Label

Here is some information about the white nationalist movement.

The white nationalist movement holds that whites are under threat in the United States and seeks to ensure the survival of the white race in this country. White nationalists believe that white people should hold more political and economic power than other groups. They believe that whites should maintain their majority in the U.S. and that white culture is superior to all other cultures.

Just to confirm your previous response, are you a supporter of the white nationalist movement?
Question Text

Ask only if *race* = 1 and respondent is assigned to Group 1
Instructions for programmers

COLUMNS
1 - Yes
2 - No

SMP119
SINGLE CHOICE
Variable Type

White nationalist - long
Variable Label

The white nationalist movement holds that whites are under threat in the United States and seeks to ensure the survival of the white race in this country. White nationalists believe that white people should hold more political and economic power than other groups. They believe that whites should maintain their majority in the U.S. and that white culture is superior to all other cultures.

Are you a supporter of the white nationalist movement?
Question Text

Ask only if *race* = 1 and respondent is assigned to Group 2.
Instructions for programmers

COLUMNS
1 - Yes
2 - No

Survey question wording - Study 2. Our survey questionnaire for Study 2, conducted in 2024 with NORC's AmeriSpeak panel, appears on the following pages.

AmeriSpeak Omnibus June 2024 Wave 2.0624.2
START OF NYU QUESTIONS wave 3 of 3
INSERT ITEM TIMESTAMPS: TIME_NYUSTART, DATE_NYUSTART

#[SHOW IF S_RACETH=1 AND P_OVER=1]
[DISPLAY]
DISPLAY_NYU.
Changing topics now…

#[SHOW IF RND_02=0 AND S_RACETH=1 AND P_OVER=1]
[SP]
NYU1.
Are you a supporter of the white nationalist movement?

CAWI RESPONSE OPTIONS:
01 Yes
02 No

CATI RESPONSE OPTIONS:
01 YES
02 NO

#[SHOW IF RND_02=0 AND S_RACETH=1 AND P_OVER=1]
[SP]
NYU2.
Here is some information about the white nationalist movement.
[SPACE]
The white nationalist movement holds that whites are under threat in the United States and seeks to ensure the survival of the white race in this country. White nationalists believe that white people should hold more political and economic power than other groups. They believe that whites should maintain their majority in the U.S. and that white culture is superior to all other cultures.
[SPACE]
Just to confirm your previous response, are you a supporter of the white nationalist movement?

CAWI RESPONSE OPTIONS:
01 Yes
02 No

CATI RESPONSE OPTIONS:
01 YES
02 NO

#[SHOW IF RND_02=1 AND S_RACETH=1 AND P_OVER=1]
[SP]
NYU3.
The white nationalist movement holds that whites are under threat in the United States and seeks to ensure the survival of the white race in this country. White nationalists believe that white people should hold more political and economic power than other groups. They believe that whites should maintain their majority in the U.S. and that white culture is superior to all other cultures.
[SPACE]
Are you a supporter of the white nationalist movement?

CAWI RESPONSE OPTIONS:
- 01 Yes
- 02 No

CATI RESPONSE OPTIONS:
- 01 YES
- 02 NO

END OF NYU QUESTIONS

Survey question wording - Study 3. Our survey questionnaire for Study 3, conducted in 2024 and 2025 with a sample recruited by CloudResearch, appears on the following pages.

Start of Block: consent

consent You are invited to participate in a survey on social groups conducted by Connect Cloud Research in conjunction with researchers from New York University.

The goal of this survey is to obtain opinions on social groups in the United States. You will be asked a number of questions about different groups. Your participation is voluntary. You are free not to answer any question or to withdraw from the study at any time. This survey should take approximately 10 minutes to complete.

A report of the results of this study will be provided to you upon request. In order to analyze responses to our questionnaire, your answers will be recorded. **No identifying information about you will be made public and any views you express will be kept completely confidential.**

Findings from this study will be reported in scholarly journals, at academic seminars, and at research association meetings. The data will be stored at a secured location and retained indefinitely. A benefit from participating in this study is that it may increase our understanding of views on social groups, and better understand causes of social tension between these groups.

Should you have questions regarding the research project, please contact Professor Patrick J. Egan (New York University) at patrick.egan@nyu.edu. For questions about your rights as a research participant, you may contact the University Committee on Activities Involving Human Subjects (UCAIHS), New York University, 665 Broadway, Suite 804, New York, New York, 10012, at ask.humansubjects@nyu.edu or (212) 998-4808. Please reference study # (IRB-FY2024-8846) when contacting the IRB (UCAIHS).

Do you agree to participate in this study?

- Yes (1)
- No (2)

End of Block: consent

Start of Block: pre_screen

1

pre_race What racial or ethnic group best describes you?

- White (1)
- Black or African-American (2)
- Hispanic or Latino (3)
- Asian or Asian-American (4)
- Native American (5)
- Middle Eastern (6)
- Mixed Race (7)
- Other (please specify below) (8)

____

Page Break

pre_hisp Are you of Spanish, Latino, or Hispanic origin or descent?

Yes (1)

No (2)

Page Break

3

pre_yob In what year were you born?

____

End of Block: pre_screen

Start of Block: kick

kick_message Thank you for your interest. Unfortunately, we already have sufficient responses of individuals matching your description. You may close this tab.

End of Block: kick

Start of Block: demographics

dem_svy How many surveys have you taken in the past month?

- This is my first (1)
- 1-3 other surveys (2)
- 4-8 other surveys (3)
- 9-15 other surveys (4)
- 16 or more other surveys (5)

Page Break

dem_gender What is your gender?

- Male (1)
- Female (2)
- Non-binary (3)
- Prefer not to say (4)

Page Break

dem_educ What is the highest level of education you have completed?

- Did not graduate from high school (1)
- High school graduate (2)
- Some college, but no degree (yet) (3)
- 2-year college degree (4)
- 4-year college degree (5)
- Postgraduate degree (MA, MBA, MD, JD, PhD, etc.) (6)

Page Break

attention1 People are very busy these days and many do not have time to follow what is happening in the government. We are testing whether people read questions. To show that you have read this much, please select "somewhat interested."

Very interested (1)
Somewhat interested (2)
Not very interested (3)
Not at all interested (4)

Page Break

dem_pid3 Generally speaking, do you think of yourself as a ...?

- Democrat (1)
- Republican (2)
- Independent (3)
- Other (please specify below) (4)
  ____
- Not sure (5)

Page Break

Display this question:

If Generally speaking, do you think of yourself as a ...? = Independent

dem_pid7_ind Do you think of yourself as closer to the Republican Party or to the Democratic Party?

- Closer to the Democratic Party (1)
- Closer to the Republican Party (2)
- Neither (3)
- Not sure (4)
- Don't know (5)

Page Break

Display this question:

If Generally speaking, do you think of yourself as a ...? = Democrat

dem_pid7_dem Would you call yourself a strong Democrat or a not very strong Democrat?

- Strong Democrat (1)
- Not very strong Democrat (2)
- Not sure (3)

Page Break

Display this question:

If Generally speaking, do you think of yourself as a ...? = Republican

dem_pid7_gop Would you call yourself a strong Republican or not very strong Republican?

- Strong Republican (1)
- Not very strong Republican (2)
- Not sure (3)

Page Break

dem_ideo5 In general, how would you describe your own political viewpoint?

- Very liberal (1)
- Liberal (2)
- Moderate (3)
- Conservative (4)
- Very conservative (5)
- Not sure (6)

Page Break

dem_vote_intent Which candidate for President of the United States did you prefer in the 2024 election?

- Donald Trump (Republican) (1)
- Kamala Harris (Democratic) (2)
- Other (please specify below) (3)
  ____
- I'm not sure (5)

End of Block: demographics

Start of Block: wn_original

wn_original Here is some information about the white nationalist movement:

The white nationalist movement holds that whites are under threat in the United States and seeks to ensure the survival of the white race in this country. White nationalists believe that white people should hold more political and economic power than other groups. They believe that whites should maintain their majority in the U.S. and that white culture is superior to all other cultures.

Are you a SUPPORTER of the white nationalist movement?

- Yes (1)
- No (2)
- Not sure (3)

End of Block: wn_original

Start of Block: wn_reverse

wn_reverse Here is some information about the white nationalist movement:

The white nationalist movement holds that whites are under threat in the United States and seeks to ensure the survival of the white race in this country. White nationalists believe that white people should hold more political and economic power than other groups. They believe that

whites should maintain their majority in the U.S. and that white culture is superior to all other cultures.

Are you an OPPONENT of the white nationalist movement?

- Yes (1)
- No (2)
- Not sure (3)

End of Block: wn_reverse

Start of Block: wn_alternate_supp

wn_alternate_supp Here is some information about the white nationalist movement:

The white nationalist movement holds that whites are under threat in the United States and seeks to ensure the survival of the white race in this country. White nationalists believe that white people should hold more political and economic power than other groups. They believe that whites should maintain their majority in the U.S. and that white culture is superior to all other cultures.

Do you SUPPORT or OPPOSE the white nationalist movement?

- I SUPPORT the white nationalist movement (1)
- I OPPOSE the white nationalist movement (2)
- Not sure (3)

End of Block: wn_alternate_supp

Start of Block: wn_alternate_opp

wn_alternate_opp Here is some information about the white nationalist movement:

The white nationalist movement holds that whites are under threat in the United States and seeks to ensure the survival of the white race in this country. White nationalists believe that white people should hold more political and economic power than other groups. They believe that whites should maintain their majority in the U.S. and that white culture

is superior to all other cultures.

Do you OPPOSE or SUPPORT the white nationalist movement?

I OPPOSE the white nationalist movement (1)
I SUPPORT the white nationalist movement (2)
Not sure (3)

End of Block: wn_alternate_opp

Start of Block: white_id_rev

white_id_rev Please indicate how much you agree or disagree with the following statements.

	Strongly disagree (1)	Somewhat disagree (2)	Neither agree nor disagree (3)	Somewhat agree (4)	Strongly agree (5)
Being white is not important to my identity. (1)					
White people in this country have much to be proud of. (2)					
Whites in this country have little in common with each other. (3)					
Whites need to do more to remind the world about the challenges that white people face. (4)					

15

Whites need to start looking out more for one another. (5)					
There is no need for a political party focused on advancing white power. (6)					

End of Block: white_id_rev

Start of Block: consp_rev

consp_rev Please indicate how much you agree or disagree with the following statements.

	Strongly disagree (1)	Somewhat disagree (2)	Neither agree nor disagree (3)	Somewhat agree (4)	Strongly agree (5)
The people who really “run” the country are not known to the voters. (1)					
Big events like wars, the recent recession, and the outcomes of elections are controlled by small groups of people who are working in secret against the rest of us. (2)					

The idea that people are controlling our lives with plots hatched in secret places is silly and false. (3)					
In our system, government decisions are transparent and visible to the public. (4)					

End of Block: consp_rev

Start of Block: white_priv_denial

white_priv_denial Please indicate how much you agree or disagree with the following statements.

	Strongly disagree (1)	Somewhat disagree (2)	Neither agree nor disagree (3)	Somewhat agree (4)	Strongly agree (5)
White people have certain advantages that minorities do not have in this society. (1)					
I feel that white skin in the United States opens many doors for whites during their everyday lives. (2)					

17

I do not feel that white people have any benefits due to their race. (3)					

End of Block: white_priv_denial

Start of Block: racial_resent

racial_resent Please indicate how much you agree or disagree with the following statements.

	Strongly disagree (1)	Somewhat disagree (2)	Neither agree nor disagree (3)	Somewhat agree (4)	Strongly agree (5)
Irish, Italians, and Jewish and many other minorities overcame prejudice and worked their way up. Blacks should do the same without any special favors. (1)					
Generations of slavery and discrimination have created conditions that make it difficult for blacks to work their way out of the lower class. (2)					

Over the past few years, blacks have gotten less than they deserve. (3)	
It's really a matter of some people just not trying hard enough: if blacks would only try harder they could be just as well off as whites. (4)	

End of Block: racial_resent

Start of Block: replacement_theory

replacement_theory Please indicate how much you agree or disagree with the following statements.

	Strongly disagree (1)	Somewhat disagree (2)	Neither agree nor disagree (3)	Somewhat agree (4)	Strongly agree (5)
There is a group of people in this country who are trying to replace native-born Americans with immigrants who agree with their political views. (1)					

Immigrants are invading our country and replacing our cultural and ethnic background. (2)
Powerful politicians and corporate leaders are trying to replace white people in the US with cheaper foreign laborers. (3)

End of Block: replacement_theory

Start of Block: old_fashioned_marry

old_fashioned_marry How would you feel if a close relative of yours were planning to marry a black person?
- Very in favor (1)
- Somewhat in favor (2)
- Neither in favor nor opposed (3)
- Somewhat opposed (4)
- Very opposed (5)

End of Block: old_fashioned_marry

Start of Block: old_fashioned_neighbors

old_fashioned_neighb How would you feel to live in a neighborhood where half of your neighbors are black?
- Very in favor (1)
- Somewhat in favor (2)
- Neither in favor nor opposed (3)
- Somewhat opposed (4)
- Very opposed (5)

End of Block: old_fashioned_neighbors

Start of Block: old_fashioned_learn

old_fashioned_learn On the average black Americans have worse jobs, income, and housing than white people. Do you think these differences are because most black Americans have less in-born ability to learn?

- Yes (1)
- No (2)

End of Block: old_fashioned_learn

Start of Block: debrief

Q37

Name of Study: Support for White Nationalist Views in the US

Thank you for your participation in this research study. For this study, it was important that information was withheld from you. Now that your participation is complete, you will be given more information about this.

What you should know about this study. The purpose of this study was to examine relationships between support for white nationalism and other measures about white identity, racism, and attitudes about immigration. We did not completely disclose this purpose to you at the beginning of the study in order to obtain responses that were as candid and honest as possible. No identifying information about you will be made public and any views you expressed will be kept completely confidential.

A report of the results of this study will be provided to you upon request. Findings from this study will be reported in scholarly journals, at academic seminars, and at research association meetings.

Right to withdraw data. Because you were not fully informed about the study during the consent process, you may choose to have your data not used for the research analysis. Please indicate below if you give permission to have your data included in the research analysis:

- I give permission for my data to be included in the study. (1)
- I DO NOT give permission for my data to be included in the research analysis. (2)

Q38 **If you have questions**

The main researcher conducting this study is Patrick J. Egan, a professor at NYU. If you have questions, you may contact Patrick J. Egan at patrick.egan@nyu.edu or at 212-992-8078. If you have any questions or concerns regarding your rights as a research participant in this study, you may contact the NYU Human Research Protection Program at ask.humansubjects@nyu.edu. Please reference study # (IRB-FY2024-8846) when contacting the IRB (UCAIHS).

End of Block: debrief

SI section B:
Descriptive statistics (Studies 1, 2 and 3)

Descriptive statistics - Study 1 (CES/YouGov)

Table S1. Sample demographic characteristics

Gender	%	Age	%	Education	%	Family income	%
Female	50.7	18-29	16.7	<HS	5.8	<$30K	25.9
Male	48.2	30-44	22.2	HS diploma	27.2	$30-$50K	19.9
Other	1.1	45-64	34.7	Some college	19.7	$50-$70K	17.1
		65+	26.5	2-year degree	8.8	$70-$100K	17.5
				4-year degree	24.3	>$100K	19.7
				Post-grad	14.2		
Total	100.0	Total	100.0	Total	100.0	Total	100.0

Attendance of relig. svcs	%	Partisanship (incl. leaned partisans)	%	Ideology	%	2020 Presidential vote	%
Weekly	22.4	Republican	44.8	Very conservative	16.1	Trump	40.6
Monthly	5.7	Democrat	36.2	Conservative	23.4	Biden	31.3
Annually	9.7	Independent	16.2	Moderate	25.7	Other	1.1
Seldom	22.6	Not sure	2.8	Liberal	15.5	Didn't vote	27.0
Never	37.4			Very liberal	11.6		
Don't know	2.1			Not sure	7.7		
Total	100.0	Total	100.0	Total	100.0	Total	100.0

Hardships in past year	% with hardship	Number of 3 closest friends known only online	%	Frequency of social media use	%	Political activities on social media, past 24 hrs	% engaged in activity
Death of loved one	28.8	0 of 3	81.2	At least 10x/day	14.9	Read story or watched video	33.0
Visited an E.R.	22.5	1 of 3	12.9	Several times/day	40.7		
Serious health threat	15.2	2 of 3	3.0	About 1x/day	16.8	Posted comment	17.2
Lost job	8.0	3 of 3	2.9	Weekly	10.2		
Ended a relationship	6.3			Less often	14.9	Forwarded story, video, or link	14.8
Pay cut at work	4.9	Total	100.0	Don't know	2.4		
Victim of crime	3.3					Posted story, video, or link	14.4
Been arrested	1.9			Total	100.0		
Divorced	0.8					Followed political event	13.1
Total hardships:	%						
0	44.3						
1	31.7						
2	16.3						
3 or more	7.8						
Total	100.0						

Table S2. Views on white identity, white power, and white nationalism

White identity and white collective action	% agreeing
"White people in this country have a lot to be proud of."	50.6%
"Being white is important to my identity."	27.0%
"Whites in this country have a lot in common with each other."	26.7%
"Whites need to start looking out more for one another."	25.0%
"Whites need to do more to remind the world about the challenges that white people face."	22.1%
"We need a political party focused on advancing white power."	7.3%

Group political power	% saying group has too much power
Evangelical Christians	38.1%
Whites	33.9%
Muslims	26.1%
Blacks	20.0%
Jews	12.1%
Hispanics	11.8%
Asians	9.1%

Threats, harassment, violence	% saying action is at least "occasionally" OK or justified
"How often do you feel it is justified for whites to use violence in advancing their political goals these days?"	8.0%
"When, if ever, is it OK for whites to send threatening and intimidating messages to non-white leaders?"	7.3%
"When, if ever, is it OK for an ordinary white person in the public to harass an ordinary non-white person on the internet, in a way that makes that person feel unsafe?"	6.5%

Support for white nationalism – question variation	% saying yes
"Are you a supporter of the white nationalist movement?"	3.8%
"The white nationalist movement holds that whites are under threat in the United States and seeks to ensure the survival of the white race in this country. White nationalists believe that white people should hold more political and economic power than other groups. They believe that whites should maintain their majority in the U.S. and that white culture is superior to all other cultures. Are you a supporter of the white nationalist movement?"	7.6%
**Combined responses of all participants after reading description**	**6.7%**

Descriptive statistics - Study 2 (NORC AmeriSpeak)

Table S3. Sample demographic characteristics

Gender	%	Age	%	Education	%	Family income	%
Female	50.9	18-29	15.5	<HS	7.4	<$30K	19.5
Male	49.1	30-44	24.3	HS diploma	27.2	$30–60K	24.0
		45-64	32.4	Some college	26.3	$60–100K	23.9
		65+	27.8	4-year degree	22.6	>$100K	32.6
				Post-grad	16.4		
Total	100.0	Total	100.0	Total	100.0	Total	100.0

Partisanship (incl. leaned partisans)	%	Ideology	%
Republican	45.6	Very conservative	14.9
Democrat	38.3	Somewhat conservative	16.4
Independent	16.1	Moderate	47.1
		Somewhat liberal	8.8
		Very liberal	12.8
Total	100.0	Total	100.0

Table S4. Support for white nationalism – question variation

	% saying yes
"Are you a supporter of the white nationalist movement?"	3.0%
"The white nationalist movement holds that whites are under threat in the United States and seeks to ensure the survival of the white race in this country. White nationalists believe that white people should hold more political and economic power than other groups. They believe that whites should maintain their majority in the U.S. and that white culture is superior to all other cultures. Are you a supporter of the white nationalist movement?"	4.3%
**Combined responses of all participants after reading description**	**4.9%**

Descriptive statistics - Study 3 (CloudResearch)

Table S5. Sample demographic characteristics

Gender	%	Age	%	Education	%	Family income	%
Female	52.4	18-29	13.9	HS or less	14.8	<$40K	25.0
Male	47.2	30-44	36.4	some college, no degree	20.1	$40–80K	31.7
Nonbinary	0.4	45-64	36.3	2-year degree	11.5	$80–125K	21.4
No response	0.1	65+	13.4	4-year degree	36.1	$125-200K	13.7
				Post-grad	17.5	>$200K	6.6
				No response	0.0	No response	1.7
Total	100.0	Total	100.0	Total	100.0	Total	100.0

Partisanship (incl. leaned partisans)	%	Ideology	%	2024 Vote	%
Republican	41.6	Very conservative	10.9	Trump	43.1
Democrat	46.2	Conservative	24.7	Harris	49.1
Independent	12.0	Moderate	25.2	Other	4.7
No response	0.2	Liberal	23.8	Not sure	3.1
		Very liberal	14.3	No response	0.1
		Not sure	1.0		
		No response	0.0		
Total	100.0	Total	100.0	Total	100.0

Table S6. Support for white nationalism by experimental treatment condition

	White nationalism item placed at... ...beginning of survey	...end of survey	Totals
"Are you ___________ of the white nationalist movement?"			
[+]a supporter	6.4%	4.0%	5.2%
N	*424*	*426*	*850*
an opponent	14.6%	14.6%	14.6%
N	*424*	*424*	*848*
"Do you _________ or _________ the white nationalist movement?"			
support or oppose	4.4%	5.1%	4.8%
N	*203*	*196*	*399*
oppose or support	5.4%	8.3%	6.9%
N	*222*	*229*	*451*
Totals	8.6%	8.5%	8.6%
N	*1,273*	*1,275*	*2,548*

Cell entries are percentages of respondents expressing support for the white nationalist movement in each treatment condition and number of respondents in each condition.
[+]This version is identical to item fielded in Study 1 and Study 2.

SI section C:
Details of tests validating our measure of support for white nationalist views (Study 1)

Test for overall treatment effect in Study 1. We perform our test for the treatment effect of being presented with a description of white nationalist ideology in Study 1 by regressing respondents expressed support for white nationalism on an indicator variable scored 1 if the subject was in the treatment group and 0 if in the control group. The coefficient on the treatment group indicator is significant at p<.001.

Table S7. Test for treatment effect

	coef<br>(se)
treatment group	**0.039***<br>**(0.011)**<br>***p* <.001**
intercept	0.038*<br>(0.006)
N	3,227
R^2	0.01

Robust standard errors in parentheses;
coefficients statistically significant at $*p < .05$.

Tests for heterogeneity of treatment effect in Study 1. We test for the heterogeneity of our treatment effect in Study 1 (that is, the effect of being provided a description of white nationalist ideology on their support for white nationalism) by respondents' attitudes about race. This is done with regressions of responses to the white nationalism question on an interaction of a treatment indicator with scales measuring respondents' strength of white identity (***identity***), opposition to minority group power (***groups***), and support for threats, intimidation, and violence against non-whites (***threats***). Descriptions of these scales can be found in *Materials and Methods*.

The coefficients relevant to these tests are shown in bold text in Table S8, Models 1, 3, and 5. They indicate that the treatment effect was stronger for respondents scoring higher on ***identity*** (Model 1, $p < .001$), ***groups*** (Model 3, $p < .001$) and ***threats*** (Model 5, $p < .10$).

We further test whether these heterogeneous effects were stronger among whites with low levels of knowledge about politics and public affairs. This is done with a triple interaction among the treatment indicator, racial attitudes, and a political knowledge scale developed from respondents' answers to survey items on the Common Content (described further below).

The coefficients relevant to our tests for heterogeneous treatment effects by political knowledge are shown in bold text in Table S8, Models 2, 4, and 6. Negative coefficients on these triple interaction terms confirm that the heterogeneity of treatment effects described in the paragraph above was stronger among those with less political knowledge. The p-values associated with these triple interaction coefficients are ***identity***: (Model 2, $p < .04$); ***groups***: (Model 4, $p < .12$); ***threats***: (Model 6, $p < .28$).

Table S8. Tests for heterogeneity of treatment effect

	identity		***groups***		***threats***	
	1	2	3	4	5	6
treatment group	-0.046*	-0.144*	-0.018	-0.008	0.033*	0.089*
	(0.018)	(0.068)	(0.011)	(0.036)	(0.010)	(0.039)
racial attitude scale (as listed at top of column)	0.202*	0.387*	0.118*	0.119	0.255*	0.135
	(0.034)	(0.130)	(0.029)	(0.074)	(0.087)	(0.157)
treatment group x racial attitude scale	**0.215***	0.522*	**0.288***	0.476*	**0.266**	0.534
	**(0.060)**	(0.185)	**(0.068)**	(0.158)	**(0.157)**	(0.328)
	***p*<.001**		***p*<.001**		***p*=.091**	
political knowledge		0.071		-0.057*		-0.063*
		(0.052)		(0.026)		(0.031)
treatment group x political knowledge		0.144		-0.004		-0.077
		(0.078)		(0.043)		(0.047)
racial attitude scale x political knowledge		-0.269		-0.014		0.196
		(0.159)		(0.094)		(0.223)
treatment group x racial attitude scale x political knowledge		**-0.468***		**-0.328**		**-0.504**
		**(0.227)**		**(0.202)**		**(0.464)**
		***p*=.039**		***p*=.104**		***p*=.278**
intercept	-0.047*	-0.100*	0.012*	0.051*	0.026*	0.068*
	(0.010)	(0.045)	(0.006)	(0.021)	(0.006)	(0.025)
N	3,177	3,153	3,227	3,200	3,205	3,180
R^2	0.14	0.18	0.11	0.14	0.06	0.09

Robust standard errors in parentheses; coefficients statistically significant at $*p < .05$.

SI section D:
Correlations between our measure of support for white nationalism and other measures of racial attitudes and conspiracy beliefs (Study 3)

Table S9. Correlation matrix of measures in Study 3

	White nationalism	White identity	Denial of white privilege	Racial resentment	Great replacement	Old-fashioned racism	Conspiracy beliefs
Support for white nationalism	1.00						
	(2548)						
White identity	0.40	1.00					
	(2530)	(2530)					
Denial of white privilege	0.15	0.49	1.00				
	(2545)	(2527)	(2545)				
Racial resentment	0.25	0.66	0.74	1.00			
	(2545)	(2527)	(2542)	(2545)			
“Great Replacement Theory” beliefs	0.31	0.70	0.56	0.68	1.00		
	(2545)	(2527)	(2542)	(2542)	(2545)		
“Old fashioned” racism	0.26	0.48	0.33	0.48	0.40	1.00	
	(2542)	(2524)	(2539)	(2539)	(2539)	(2542)	
Conspiracy beliefs	0.15	0.28	0.21	0.25	0.44	0.12	1.00
	(2543)	(2525)	(2540)	(2540)	(2540)	(2537)	(2543)

Cells report pairwise correlations and valid *N*.

SI section E:
Robustness of estimates to respondent inattentiveness (Studies 1 and 3)

A concern raised by studies relying on a simple yes-or-no question of the kind we use to measure support for white nationalist ideology is that inattentive survey respondents may answer such questions at random, biasing estimates toward 50 percent. This becomes more of a concern with a yes-or-no survey question like ours that is measuring a population parameter whose true mean is relatively close to zero, as even a small degree of random response can result in estimates that are substantially larger than the population mean (2). Given that previous work has shown that the time respondents take to complete a survey is negatively correlated with inattentiveness (3, 4), here we analyze the relationship between survey completion time and support for white nationalism to assess the extent to which inattentiveness may bias our estimates.

Study 1. Respondents were permitted to take as long as they wished to complete our survey in Study 1, resulting in a highly skewed distribution of minutes to completion (min = 8.7; median = 26.3; max = 27,401.1). See Figure S1. A bivariate regression of support for white nationalism on minutes taken for survey completion recovers a statistically significant ($p < .001$) coefficient of .00000381. This is substantively small compared to our population estimate of 6.7 percent: each additional hour of survey completion time is associated with a decrease in support for white nationalism of just .02 percentage points.

The skewed distribution of completion times warrants an analysis that allows for non-linearity in the relationship between completion time and support for white nationalism. We do this by dividing our sample into quantiles according to the time each respondent took to complete the survey as measured by the CES. We do this in three ways, dividing the sample into 5, then 10, and then finally 20 such quantiles. Table S10 indicates the minimum, mean and maximum number of minutes taken to the complete the survey for each of the quantiles.

To assess the extent to which survey completion time was independently associated with support for white nationalism, we regressed respondents' expressed support for white nationalism on indicator variables for each quantile, plus the three scales displayed in Figure 1 (strength of white identity; opposition to minority group power, and support for threats against nonwhites), as well as all of the covariates displayed in Figure 2: age, gender and their interaction; education; income; attendance of religious services; ideology; party identification; 2020 presidential vote; and conspiratorial beliefs. Estimates are displayed in Table S11.

The coefficients on the quantile indicators are thus estimates of the extent to which survey time completion is associated with support for white nationalism after controlling for other predictors which themselves may be associated with survey completion time. We examine these coefficients first with tests of their joint significance, which fail to reject the null when respondents are divided into 5 quantiles ($p = .85$) or 20 quantiles ($p = .24$) of survey time completion, and marginally significant when respondents are divided into 10 quantiles ($p = .08$). We then plot (Figure S2) these coefficients' values as average predictive margins–that is, the average predicted level of support for white nationalism in our sample associated with each quantile, holding all other covariates at their actual values. Our population estimate of .067 is plotted with a horizontal blue line for comparison. Each of these plots indicates that those in the fastest quantile were unusually high in their support for white nationalism, but reassuringly that the *ceteris paribus* relationship between survey completion time and support for white nationalism is non-monotonic.

Taken together, these findings indicate that estimates of support for white nationalism robust to concerns about inattentiveness can be obtained by dropping the respondents in the fastest quantiles and recalculating the estimated mean. We do this in Figure S3 for all of the quantiles in the analysis, generating alternate estimates of support for white nationalism by iteratively dropping one quantile at a time and recalculating the estimated mean support. This produces estimates ranging between 6.0 percent (after dropping the fastest 20 percent of the sample) to 6.4 percent (after dropping the fastest 5 percent of the sample). These results are reassuringly close to the estimate of 6.7 percent derived from our entire sample.

Study 3. We repeated the analysis performed in Table S11 with data from Study 3. We once again regressed respondents' expressed support for white nationalism on indicator variables for respondents divided into 5, 10, and 20 time quantiles. As shown in Table S12, all three analyses failed to reject the null of no joint significance of the coefficients on these indicators (at $p = .48$, $p = .58$, and $p = .89$ respectively), ruling out concerns that response time was associated with support for white nationalism in Study 3.

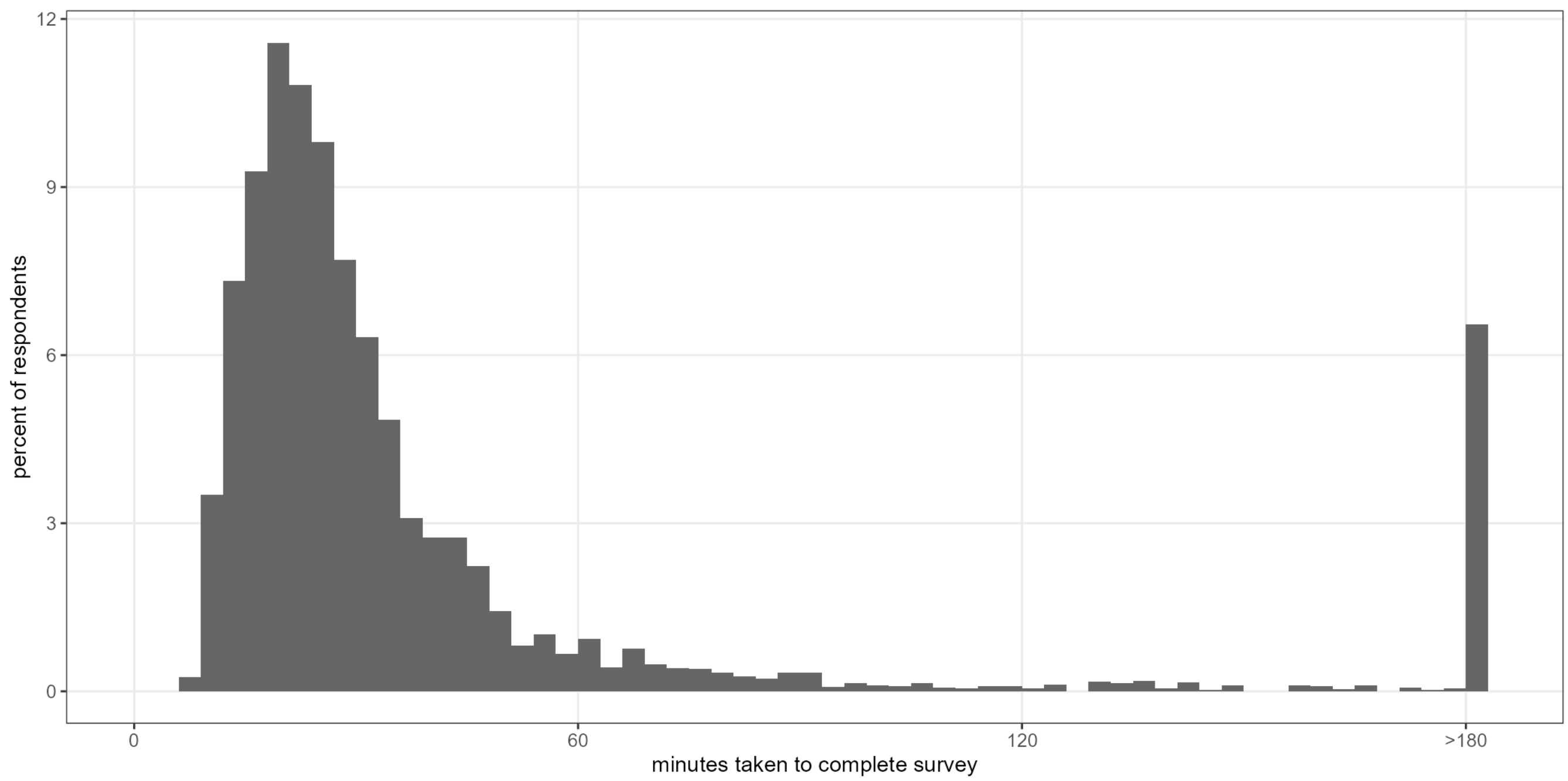

Fig. S1. Distribution of survey completion times

Table S10. Summary statistics for quantiles of minutes taken to complete survey

	minutes taken			
5 quantiles	min	mean	max	freq
1	8.7	14.5	17.9	646
2	17.9	20.5	23.0	646
3	23.1	26.1	29.5	646
4	29.5	35.1	43.4	645
5	43.5	816.4	27,401.1	644
Total	8.7	182.2	27,401.1	3,227

	minutes taken			
10 quantiles	min	mean	max	freq
1	8.7	12.5	14.7	323
2	14.7	16.4	17.9	323
3	17.9	19.2	20.4	326
4	20.5	21.7	23.0	320
5	23.1	24.5	26.0	322
6	26.0	27.6	29.5	324
7	29.5	31.8	34.3	322
8	34.4	38.4	43.4	323
9	43.5	54.9	77.2	322
10	77.6	1,577.9	27,401.1	322
Total	8.7	182.2	27,401.1	3,227

	minutes taken			
20 quantiles	min	mean	max	freq
1	8.7	11.2	12.7	162
2	12.7	13.8	14.7	161
3	14.7	15.7	16.5	162
4	16.5	17.2	17.9	161
5	17.9	18.6	19.1	162
6	19.1	19.8	20.4	164
7	20.5	21.1	21.7	158
8	21.7	22.4	23.0	162
9	23.1	23.8	24.4	161
10	24.4	25.2	26.0	161
11	26.0	26.8	27.5	162
12	27.6	28.5	29.5	162
13	29.5	30.6	31.8	161
14	31.8	33.0	34.3	161
15	34.4	36.1	38.1	161
16	38.1	40.7	43.4	162
17	43.5	47.2	52.0	160
18	52.1	62.6	77.2	162
19	77.6	132.6	266.8	161
20	276.2	3,023.1	27,401.1	161
Total	8.7	182.2	27,401.1	3,227

Table S11. Tests of joint significance of survey completion time quantiles as predictors of support for white nationalism in Study 1, controlling for all covariates in Figures 1 and 2

	5 quantiles		10 quantiles		20 quantiles	
survey completion time quantile (excluded category: 1)						
quantile 2	-0.017	(0.016)	-0.004	(0.026)	-0.032	(0.041)
quantile 3	-0.008	(0.018)	0.006	(0.027)	-0.010	(0.036)
quantile 4	-0.012	(0.018)	-0.046*	(0.022)	-0.031	(0.038)
quantile 5	-0.015	(0.016)	-0.017	(0.026)	-0.017	(0.036)
quantile 6			-0.005	(0.027)	-0.002	(0.040)
quantile 7			0.004	(0.029)	-0.048	(0.033)
quantile 8			-0.033	(0.024)	-0.075*	(0.031)
quantile 9			-0.003	(0.025)	-0.020	(0.039)
quantile 10			-0.032	(0.023)	-0.049	(0.032)
quantile 11					-0.025	(0.039)
quantile 12					-0.017	(0.036)
quantile 13					-0.018	(0.042)
quantile 14					-0.007	(0.039)
quantile 15					-0.048	(0.036)
quantile 16					-0.050	(0.033)
quantile 17					-0.000	(0.040)
quantile 18					-0.036	(0.033)
quantile 19					-0.053	(0.030)
quantile 20					-0.043	(0.035)
age (excluded category: 18-29)						
30-44	-0.050	(0.037)	-0.048	(0.037)	-0.045	(0.036)
45-64	-0.091*	(0.033)	-0.090*	(0.033)	-0.089*	(0.033)
65+	-0.091*	(0.034)	-0.087*	(0.033)	-0.087*	(0.033)
gender (excluded category: male)						
Female	-0.039	(0.035)	-0.036	(0.034)	-0.037	(0.034)
Other	-0.059	(0.031)	-0.060	(0.031)	-0.061	(0.032)
age 30-44 x Female	0.025	(0.041)	0.024	(0.041)	0.023	(0.041)
age 30-44 x Other	0.013	(0.073)	0.010	(0.073)	0.006	(0.074)
age 45-64 x Female	0.062	(0.037)	0.061	(0.037)	0.063	(0.037)
age 45-64 x Other	0.064	(0.039)	0.063	(0.041)	0.062	(0.043)
age 65+ x Female	0.068	(0.038)	0.065	(0.038)	0.067	(0.038)
age 65+ x Other	0.163*	(0.042)	0.172*	(0.042)	0.170*	(0.048)
education (excluded category: no HS)						
High school graduate	-0.086*	(0.039)	-0.088*	(0.039)	-0.089*	(0.039)
Some college	-0.080*	(0.039)	-0.084*	(0.039)	-0.083*	(0.039)
2-year	-0.089*	(0.039)	-0.090*	(0.039)	-0.090*	(0.039)
4-year	-0.093*	(0.038)	-0.094*	(0.038)	-0.095*	(0.038)
Post-grad	-0.108*	(0.039)	-0.109*	(0.039)	-0.111*	(0.038)
ideology (excluded category: very liberal)						
Liberal	-0.023	(0.013)	-0.022	(0.013)	-0.022	(0.013)
Moderate	-0.050*	(0.018)	-0.051*	(0.018)	-0.050*	(0.018)
Conservative	-0.054	(0.030)	-0.052	(0.030)	-0.052	(0.029)
Very conservative	-0.049	(0.032)	-0.050	(0.032)	-0.049	(0.032)
Not sure	-0.034	(0.035)	-0.036	(0.035)	-0.034	(0.035)
Party identification (excluded category: Democrat)						
Independent	-0.017	(0.022)	-0.015	(0.021)	-0.015	(0.021)
Republican	-0.024	(0.026)	-0.022	(0.025)	-0.022	(0.025)
Not sure	-0.015	(0.055)	-0.017	(0.054)	-0.019	(0.055)
2020 vote (excluded category: Trump)						
Biden	0.034	(0.022)	0.035	(0.022)	0.035	(0.022)
Other	-0.027	(0.025)	-0.023	(0.025)	-0.021	(0.025)
Did not vote	0.005	(0.020)	0.005	(0.020)	0.005	(0.020)
Attendance of relig svcs (excluded category: weekly)						
Monthly	0.019	(0.032)	0.020	(0.031)	0.018	(0.031)
Yearly	-0.008	(0.022)	-0.005	(0.022)	-0.005	(0.023)
Seldom	-0.042*	(0.017)	-0.040*	(0.017)	-0.041*	(0.017)
Never	-0.023	(0.018)	-0.021	(0.018)	-0.021	(0.018)
Don't know	-0.040	(0.054)	-0.039	(0.055)	-0.038	(0.055)
Income (excluded category <$30K)						
21-40% (30−50K)	-0.018	(0.023)	-0.017	(0.023)	-0.017	(0.023)
41-60% (50−70K)	-0.013	(0.023)	-0.012	(0.023)	-0.011	(0.023)
61-80% (70−100K)	0.007	(0.024)	0.008	(0.024)	0.008	(0.024)
81-100% (>$100K)	-0.001	(0.022)	0.001	(0.023)	0.001	(0.023)
missing data	-0.025	(0.025)	-0.023	(0.025)	-0.023	(0.025)
White identity scale	0.304*	(0.034)	0.302*	(0.034)	0.301*	(0.034)
Opposition to non-white power scale	0.198*	(0.040)	0.199*	(0.040)	0.199*	(0.040)
Support for threats/harassment/violence scale	0.325*	(0.086)	0.327*	(0.086)	0.324*	(0.085)
Conspiracy beliefs scale	-0.000*	(0.000)	-0.000*	(0.000)	-0.000*	(0.000)
intercept	0.141*	(0.059)	0.137*	(0.061)	0.154*	(0.067)
N	3,139		3,139		3,139	
R^2	0.23		0.23		0.23	
test for joint significance of survey completion quantiles	F = 0.34; df = 4; p = .85		F = 1.72; df = 9; p = .08		F = 1.21; df = 19; p = .24	

Robust standard errors in parentheses; coefficients statistically significant at $^*p < .05$

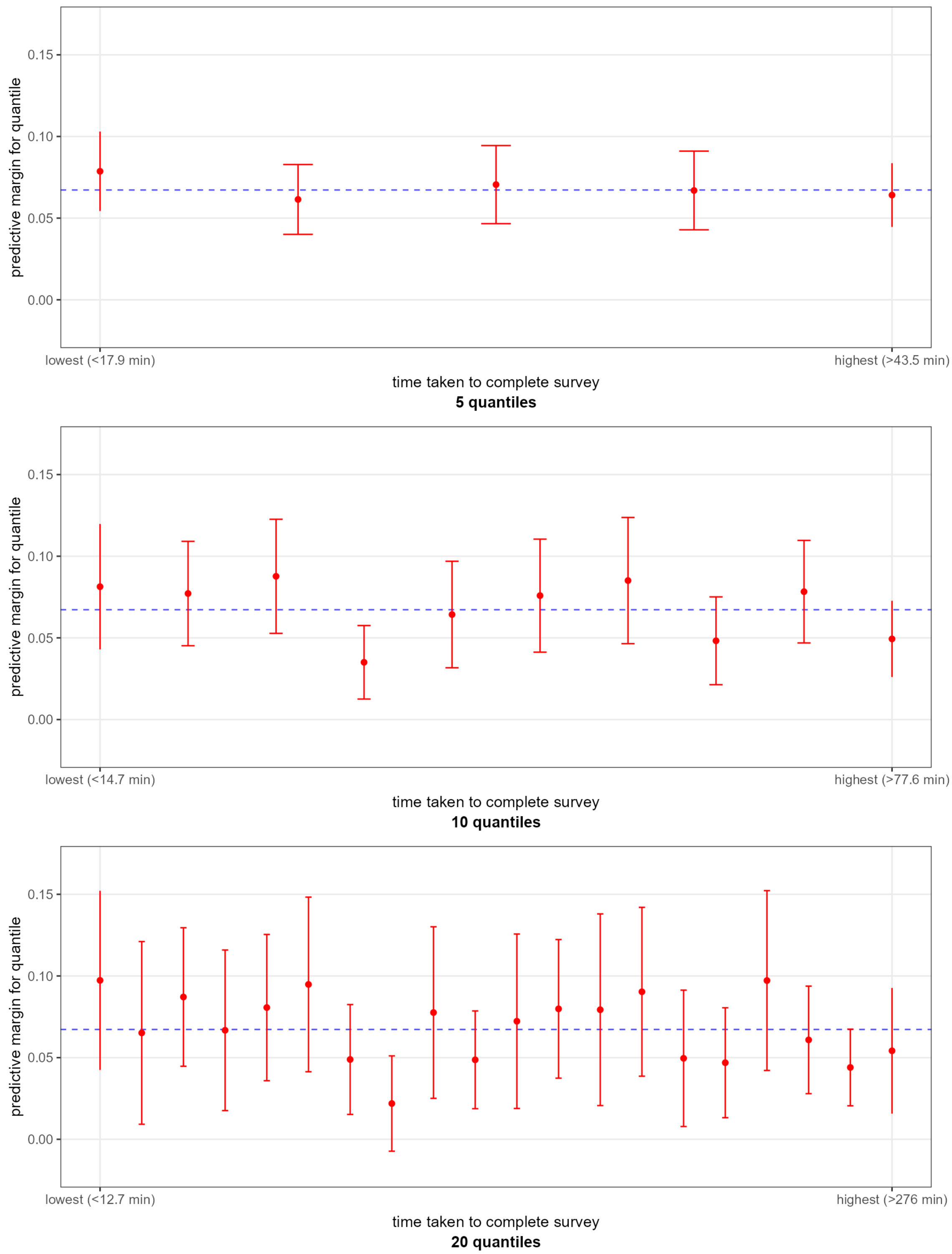

Fig. S2. Average predictive margins associated with survey completion time quantiles, controlling for all covariates in Figures 1 and 2

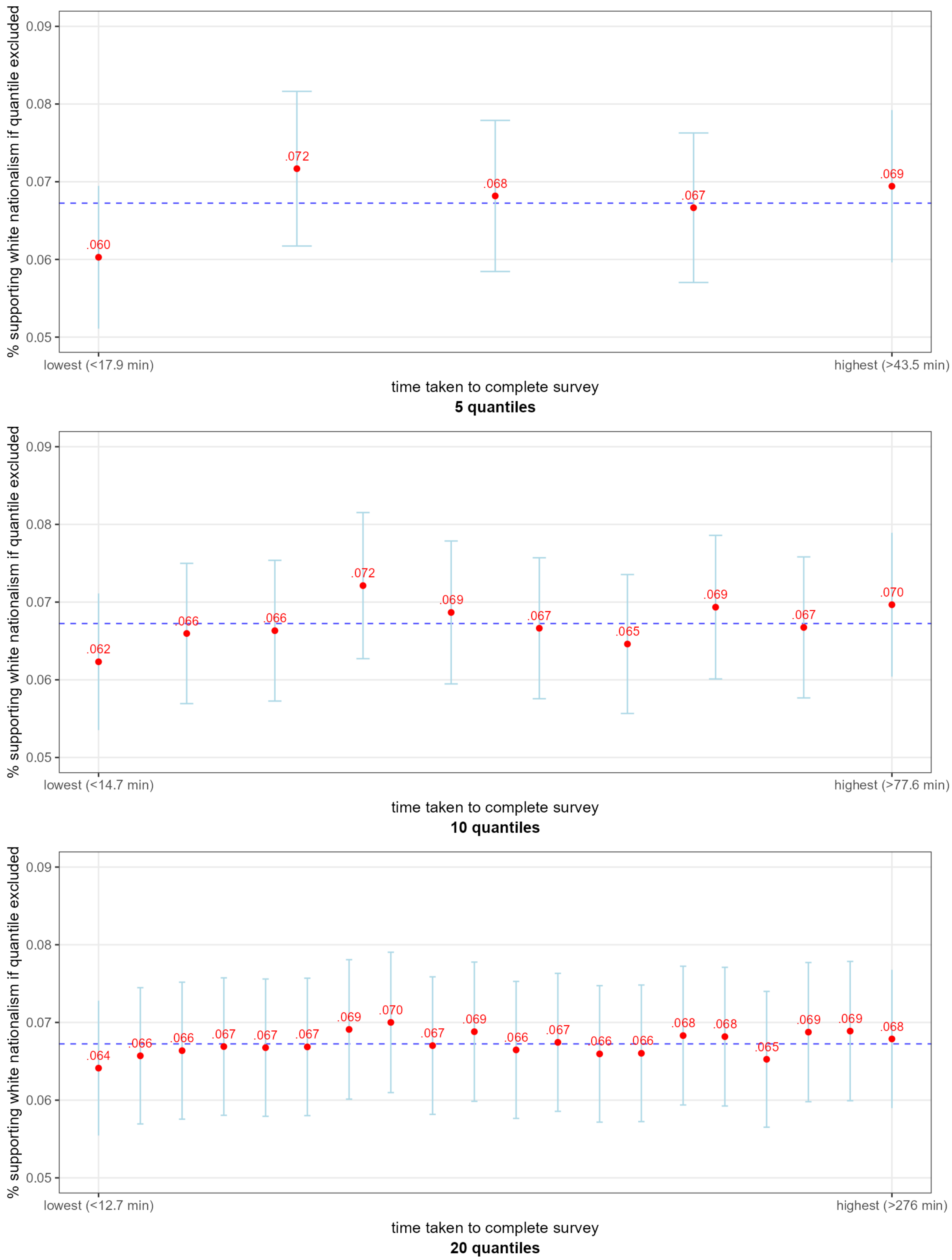

Fig. S3. Estimates of support for white nationalism derived from iteratively excluding respondents in each survey completion time quantile

Table S12. Tests of joint significance of survey completion time quantiles as predictors of support for white nationalism in Study 3, controlling for all relevant covariates

	5 quantiles	10 quantiles	20 quantiles
survey completion time quantile (excluded category: 1)			
quantile 2	-0.022 (0.022)	-0.005 (0.031)	-0.022 (0.044)
quantile 3	-0.010 (0.022)	-0.004 (0.031)	-0.009 (0.045)
quantile 4	-0.029 (0.022)	-0.043 (0.031)	-0.025 (0.044)
quantile 5	0.006 (0.022)	-0.018 (0.031)	-0.008 (0.044)
quantile 6		-0.007 (0.032)	-0.025 (0.045)
quantile 7		-0.028 (0.031)	-0.068 (0.044)
quantile 8		-0.036 (0.032)	-0.041 (0.046)
quantile 9		0.028 (0.032)	-0.024 (0.044)
quantile 10		-0.020 (0.031)	-0.037 (0.045)
quantile 11			-0.007 (0.044)
quantile 12			-0.034 (0.046)
quantile 13			-0.016 (0.045)
quantile 14			-0.062 (0.044)
quantile 15			-0.057 (0.046)
quantile 16			-0.040 (0.045)
quantile 17			0.023 (0.045)
quantile 18			0.007 (0.045)
quantile 19			-0.007 (0.044)
quantile 20			-0.060 (0.046)
age (excluded category: 18-29)			
30-44	-0.056 (0.031)	-0.057 (0.031)	-0.058 (0.031)
45-64	-0.089* (0.032)	-0.087* (0.032)	-0.087* (0.033)
65+	-0.127* (0.041)	-0.124* (0.042)	-0.125* (0.042)
gender (excluded category: male)			
female	-0.095* (0.039)	-0.093* (0.039)	-0.098* (0.039)
female $\times$ 30-44	0.055 (0.045)	0.054 (0.045)	0.058 (0.046)
female $\times$ 45-64	0.103* (0.045)	0.098* (0.045)	0.099* (0.046)
female $\times$ 65+	0.114* (0.054)	0.110* (0.054)	0.113* (0.055)
education (excluded category: HS or less)			
some college	-0.005 (0.023)	-0.007 (0.023)	-0.008 (0.024)
2-year	-0.031 (0.026)	-0.030 (0.026)	-0.025 (0.026)
BA	0.044* (0.022)	0.044* (0.022)	0.044* (0.022)
post grad	0.034 (0.026)	0.035 (0.026)	0.035 (0.026)
income (excluded category: <$40K)			
$40-80K	-0.025 (0.018)	-0.026 (0.018)	-0.026 (0.019)
$80-125K	0.005 (0.022)	0.008 (0.022)	0.007 (0.022)
$125-200K	-0.077* (0.024)	-0.078* (0.024)	-0.078* (0.024)
$200K+	-0.046 (0.031)	-0.047 (0.032)	-0.052 (0.032)
ideology (excluded category: very liberal)			
liberal	0.006 (0.024)	0.004 (0.024)	0.005 (0.024)
moderate	-0.023 (0.029)	-0.027 (0.029)	-0.024 (0.030)
conservative	-0.040 (0.037)	-0.043 (0.037)	-0.035 (0.038)
very conservative	0.056 (0.042)	0.052 (0.042)	0.060 (0.043)
not sure	-0.061 (0.075)	-0.060 (0.076)	-0.054 (0.076)
party ID, with leaners (excluded category: Democratic)			
Independent	0.014 (0.029)	0.014 (0.029)	0.010 (0.030)
Republican	-0.006 (0.035)	-0.008 (0.035)	-0.013 (0.036)
2024 vote (excluded category: Trump)			
Harris	0.011 (0.032)	0.008 (0.032)	0.010 (0.032)
Other	-0.008 (0.036)	-0.015 (0.037)	-0.015 (0.037)
Not sure	-0.028 (0.044)	-0.026 (0.044)	-0.021 (0.044)
White identity scale	0.082* (0.012)	0.081* (0.012)	0.083* (0.012)
Conspiracy beliefs scale	0.014 (0.008)	0.014 (0.008)	0.014 (0.008)
Denial of white privilege scale	-0.007 (0.011)	-0.008 (0.011)	-0.008 (0.011)
Racial resentment scale	-0.023 (0.014)	-0.022 (0.014)	-0.022 (0.014)
Great replacement theory belief scale	0.018 (0.012)	0.019 (0.012)	0.018 (0.012)
Old-fashioned racism scale	0.031* (0.009)	0.032* (0.009)	0.031* (0.009)
Intercept	0.155* (0.048)	0.162* (0.051)	0.174* (0.058)
N	818	818	818
R^2	.24	.24	.25
test for joint significance of survey completion time profiles	$F = .88$<br>$df = 4$<br>$p = .48$	$F = .84$<br>$df = 9$<br>$p = .58$	$F = .61$<br>$df = 19$<br>$p = .90$

SI section F:
Details of survey experiments assessing acquiescence bias and priming effects (Study 3)

Ruling out acquiescence bias. We investigated whether our yes-no survey item about supporting the white nationalist movement could be subject to acquiescence bias—that is, the tendency to agree with statements in surveys regardless of actual beliefs. Study 3 featured an experiment (following (5)) in which after being provided the description of white nationalist beliefs, half of respondents were randomly assigned our *positive-keyed* original question ("Are you a SUPPORTER of the white nationalist movement?") and the other half were asked a *negative-keyed* version of the question ("Are you an OPPONENT of the white nationalist movement?"). Both of these items featured the response set of "yes," "no," and "not sure." In line with the guidance in (5), we coded "not sure" as not agreeing with the question in both conditions. Therefore endorsement of white nationalistic views was indicated with a "yes" in the positive-keyed item, but with a "no" or "not sure" in the negative-keyed item.

If responses to the positive-keyed item (which was identical to the item we fielded in Studies 1 and 2) were free of acquiescence bias, the share of respondents coded as endorsing white nationalist views should be equivalent in both conditions. But if acquiescence bias were present, it would push endorsement rates of white nationalism in response to the positive-keyed item to be higher than to the negative-keyed item.

Table S13. Acquiescence Bias Test

	Question version	
**Response**	**Positive-keyed**<br>*(""Are you a SUPPORTER of...."")*	**Negative-keyed**<br>*(""Are you an OPPONENT of...."")*
Yes	**5.2%***	74.3%
	44	*630*
No	84.7%	**14.6%***
	720	*124*
Not sure	10.1%	11.1%
	86	*94*
**Total**	**100.0%**	**100.0%**
	N = 850	*N = 848*

**Coded as endorsement of white nationalism.*
Negative-keyed item elicits statistically significantly greater rate of endorsement (t-test $p < .001$).

As shown in Table S13, this test ruled out acquiescence bias as a concern on our positive-keyed original item: the negative-keyed item in fact elicited greater rates of endorsement of white nationalism (14.6% responded "no") than our positive-keyed item (where 5.2% responded "yes," an endorsement rate in line with those in Studies 1 and 2).

To further rule out acquiescence bias and to confirm that our findings were robust to alternative question wording, another set of participants in Study 3 were provided the description of white nationalist beliefs and then randomly assigned to either be presented with the question “Do you SUPPORT or OPPOSE the white nationalist movement?” with response options in this order: “I SUPPORT the white nationalist movement”, “I OPPOSE the white nationalist movement” and “Not sure.” The other half were presented with the reverse-ordered question “Do you OPPOSE or SUPPORT the white nationalist movement?” with response options presented in this order:“I OPPOSE the white nationalist movement”, “I SUPPORT the white nationalist movement” and “Not sure.”

Table S14. Response Option Order Effects Test

**Response**	**Option listed first**	
	**SUPPORT listed first** *(“”DO you SUPPORT or OPPOSE....“”)*	**OPPOSE listed first** *(“”Do you OPPOSE or SUPPORT ....“”)*
I SUPPORT the white nationalist movement	4.8% *19*	6.9% *31*
I OPPOSE the white nationalist movement	83.2% *332*	82.5% *372*
Not sure	12.0% *48*	10.6% *48*
**Total**	**100.0%** *N = 399*	**100.0%** *N = 451*

No significant differences in support rates in the two conditions (t-test $p = .19$).

As shown in Table S14, these results were opposite that we would expect if acquiescence bias or inattentive respondents artificially boosted the share of respondents choosing the first option offered. Those given the OPPOSE option first were slightly more likely to select SUPPORT (6.9% did so) than those given the SUPPORT option first (4.8%). A differences-in-means test failed to reject the null that rates were equal in the two conditions ($p = .19$). In addition, endorsement rates in the two conditions (4.8% and 6.9%) were similar to that elicited by our original item in Study 3 as reported above (5.2%), providing further reassurance that our overall prevalence estimates are not artifacts of particular question wording.

Generalizing beyond Study 3’s sample regarding acquiescence bias. We indirectly investigate how well our findings from Study 3’s Connect sample might generalize to other samples with regard to attentiveness and acquiescence bias as follows. We start by splitting Study 3 respondents into quartiles based upon their survey completion time. We then examine their responses by quartile to both the positive- and negative-keyed versions of our white nationalism item described above.

Survey completion time is a recognized proxy for respondent attentiveness. We can thus consider these quartiles of Study 3 participants as four samples of respondents who vary with regard to their degree of attentiveness, from least attentive (the fastest respondents) to most attentive (the slowest respondents). If patterns in responses to the positive- and negative-keyed versions are consistent across these four groups, then we gain some confidence that our Study 3 findings can generalize to other samples regardless of their respondents’ level of attentiveness. If instead we see response patterns that vary with completion time, we become less confident about generalization.

As shown in Figure S4 below, the pattern of results is remarkably stable across completion time (and thus, we believe, respondent attentiveness). Support for white nationalism derived from our

positive-keyed item is always less than that yielded by the negative-keyed item across all four quartiles. Critically, there is no evidence that support registered on our positive-keyed measure (which would be most vulnerable to acquiescence bias) increases among less attentive respondents: if anything, the least attentive quartile yields a lower endorsement rate on the positive-keyed item than the most attentive quartile, the opposite of what an acquiescence-bias account would predict.

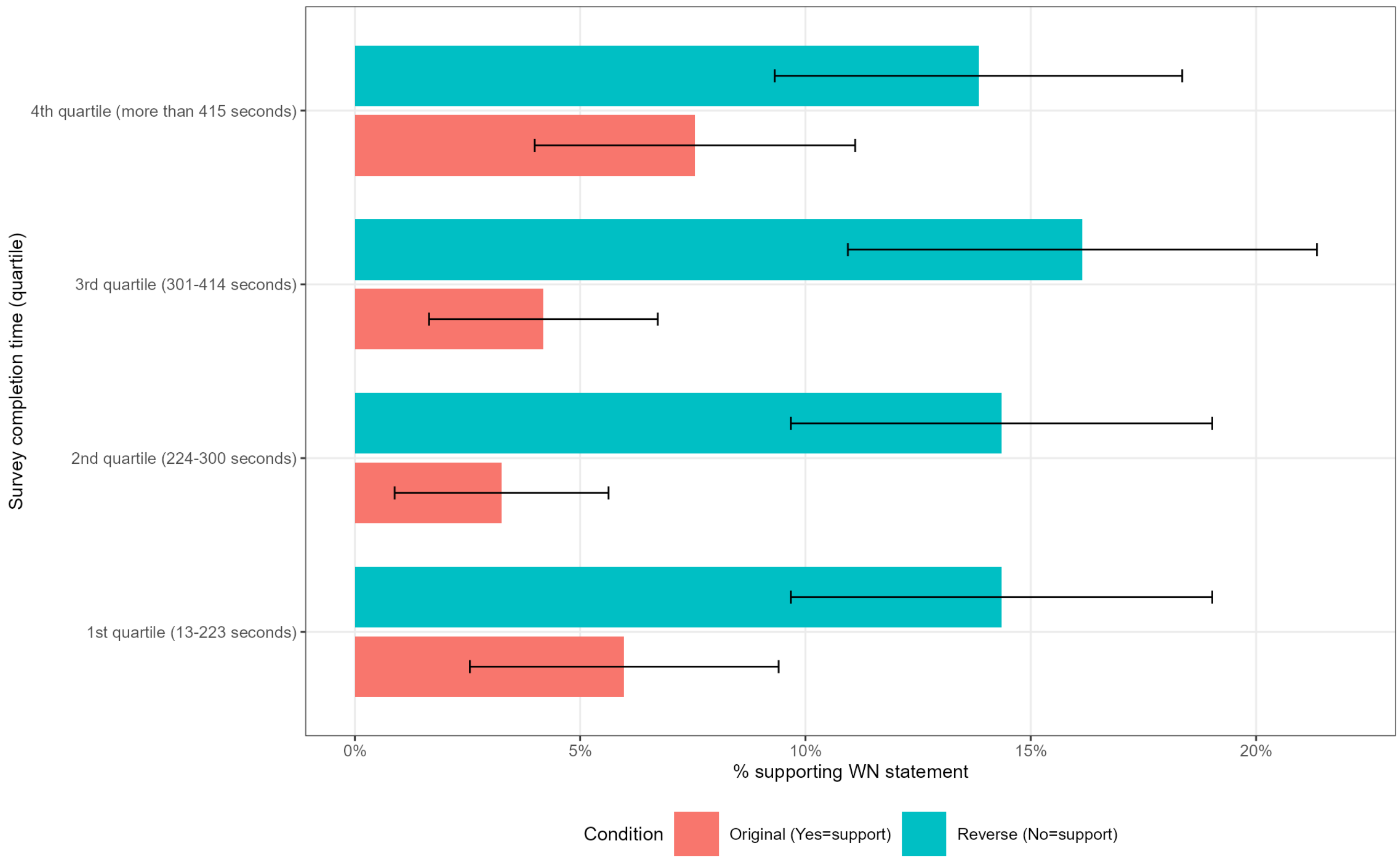

Fig. S4. Expressed support for white nationalism by question-wording condition and quartiles of survey completion time in Study 3.

Ruling out priming effects. A final concern is that our prevalence estimates in Study 1 may have been biased upward by priming effects (6), because in that study our measure of support for white nationalism was placed at the end of a survey which contained an extensive series of questions about racial attitudes. Study 3 investigated this concern with an experiment in which half of participants were randomly assigned to answer a question about their support for white nationalism before responding to any of the other items in the survey about racial attitudes. The other half was assigned to do this at the conclusion of the survey, which included several batteries of items related to racial attitudes, including white identity, racial resentment, and "old-fashioned" racism.

To conduct this test of priming effects, we pooled all responses to the four versions of the white nationalism item described above, categorizing all responses as either endorsing or not endorsing white nationalism. If exposure to batteries of questions about racial attitudes were to have a priming effect, the endorsement rate would be higher among those assigned to respond to the white nationalism item at the end of the survey than those at the beginning.

Table S15. Item Placement Effects Test

	White nationalism item placed at...	
**Response**	**...beginning of survey**	**...end of survey**
Endorses white nationalism	8.6%	8.5%
	110	*108*
Does not endorse white nationalism	91.4%	91.5%
	1,163	*1,167*
**Total**	**100.0%**	**100.0%**
	1,273	*1,275*

No significant differences in support rates in the two conditions (t-test $p = .88$).

As shown in Table S15, no evidence for priming effects was found: this test failed to reject the null that rates were equal in the two conditions (t-test $p = .88$).

SI section G:
Estimation of relationship between support for white nationalism and demographic and political covariates (Studies 1 and 2); robustness of relationships to acquiescence bias (Study 3)

Estimation of main results (Study 1). Figure 2 in the main text plots the predictors of support for white nationalist views, and includes coefficient estimates from bivariate regressions of white nationalist support on a series of demographic and political covariates in Study 1. We run these regressions two ways. To generate the coefficient estimates displayed in Figure 2, we regress white nationalism support on categorical versions of these predictors (Table S16) by ordering each covariate from the notional "least" to "most" categories (e.g., education is ordered from "No high school" to "Post-grad", income is ordered from lowest to highest quintile, ideology is ordered from left to right). We then estimate the regression with indicator variables for each of these levels of the predictor, using the "least" category as the reference point. The coefficients reported in Figure 2 are those associated with the "most" category relative to the "least" category. For example, the specification for conspiracy beliefs can be expressed as follows:

$$\begin{aligned} wn_i = \alpha \\ &+ \beta_2 \mathbb{I}(\textit{Second quintile}_i) \\ &+ \beta_3 \mathbb{I}(\textit{Third quintile}_i) \\ &+ \beta_4 \mathbb{I}(\textit{Fourth quintile}_i) \\ &+ \beta_5 \mathbb{I}(\textit{Top quintile}_i) + \varepsilon_i \end{aligned}$$

where the bottom quintile of belief in conspiracy theories is contained in the intercept α. Figure 2 reports the β_5 coefficient and its 95% confidence interval, capturing the difference between the top and bottom quintiles.

To perform the significance tests reported in the text on the associations between these predictors and white nationalism support, we regress support on continuous versions of the predictors (Table S17). In both sets of analyses, male and female age groups are estimated separately. We drop the "Not sure" categories for ideology and partisanship, and the "Did not vote" category for 2020 vote. The coefficients reported represent the difference between very conservative and very liberal respondents, Republican and Democratic respondents, and Trump and Biden voters.

Table S16. Demographic and political correlates of support for white nationalist views: as categorical predictors (Study 1; source of predictions displayed in Figure 2)

	age among men	age among women	education	income	relig. svcs. attendance	ideology	party ID	2020 presidential vote	conspiracy beliefs scale
intercept	0.169*	0.064*	0.171*	0.109*	0.092*	0.029*	0.039*	0.083*	0.025*
	(0.040)	(0.016)	(0.051)	(0.022)	(0.013)	(0.012)	(0.008)	(0.008)	(0.008)
age 30-44	-0.072	-0.025							
	(0.045)	(0.019)							
age 45-64	-0.121*	-0.009							
	(0.041)	(0.019)							
age 65+	-0.128*	0.013							
	(0.042)	(0.027)							
High school graduate			-0.087						
			(0.053)						
Some college			-0.094						
			(0.053)						
2-year			-0.097						
			(0.054)						
4-year			-0.128*						
			(0.052)						
Post-grad			-0.151*						
			(0.052)						
income 21-40% ($30-$50K)				-0.038					
				(0.026)					
income 41-60% ($50-$70K)				-0.047					
				(0.025)					
income 61-80% ($70-$100K)				-0.036					
				(0.027)					
income 81-100% (>$100K)				-0.066*					
				(0.024)					
attendance: monthly					0.008				
					(0.037)				
attendance: yearly					-0.003				
					(0.027)				
attendance: seldom					-0.049*				
					(0.016)				
attendance: never					-0.039*				
					(0.016)				
Liberal						0.001			
						(0.015)			
Moderate						0.035*			
						(0.017)			
Conservative						0.042*			
						(0.018)			
Very conservative						0.078*			
						(0.020)			
Independent							0.032		
							(0.019)		
Republican							0.048*		
							(0.012)		
2020 Vote: Biden								-0.056*	
								(0.010)	
Conspiracy beliefs: 2nd quintile									0.042*
									(0.018)
Conspiracy beliefs: 3rd quintile									0.061*
									(0.019)
Conspiracy beliefs: 4th quintile									0.028*
									(0.012)
Conspiracy beliefs: 5th quintile									0.086*
									(0.018)
N	1,460	1,727	3,227	2,946	3,169	3,043	3,158	2,757	3,210
R^2	.03	.00	.02	.01	.01	.01	.01	.01	.01

Robust standard errors in parentheses; coefficients statistically significant at $*p < .05$.

Table S17. Demographic and political correlates of support for white nationalist views: as continuous predictors (Study 1; source of significance tests reported in text)

	age among men	age among women	education	income	relig. svcs. attendance	ideology	party ID	2020 presidential vote	conspiracy beliefs scale
Age	-0.002*	0.000							
	(0.001)	(0.000)							
	p< 0.001	p= 0.372							
Education			-0.019*						
			(0.004)						
			p< 0.001						
Income				-0.001*					
				(0.000)					
				p= 0.012					
Attendance of relig svcs (weekly -> never)					-0.012*				
					(0.004)				
					p= 0.004				
Ideology (very liberal -> very conservative)						0.020*			
						(0.004)			
						p< 0.001			
Party ID (Democrat -> Republican)							0.024*		
							(0.006)		
							p< 0.001		
Vote in 2020 presidential election (Trump -> Biden)								-0.056*	
								(0.010)	
								p< 0.001	
Conspiracy beliefs scale									0.081*
									(0.018)
									p< 0.001
intercept	0.202*	0.038	0.137*	0.099*	0.106*	-0.000	0.017	0.083*	0.029*
	(0.040)	(0.022)	(0.019)	(0.015)	(0.016)	(0.013)	(0.013)	(0.008)	(0.009)
N	1,460	1,727	3,227	2,946	3,169	3,043	3,158	2,757	3,210
R^2	.02	.00	.01	.00	.01	.01	.01	.01	.01

Robust standard errors in parentheses; coefficients statistically significant at $*p < .05$

Replication of main results with probability-based sample (Study 2). We confirmed that relationships between key covariates and endorsement of white nationalism found in Study 1 held with the probability-based sample collected in Study 2. (See *Methods* for a description of the sample.) We use these analyses to replicate Figure 2 in the text with Study 2 data (see Figure S5 below).

Table S18. Replication of demographic and political correlates of support for white nationalist views: as continuous predictors (Study 2; source of significance tests reported in text)

	age among men	age among women	education	income	ideology	party ID
Age	-0.002*	-0.000				
	(0.001)	(0.001)				
	p=0.006	*p*=0.868				
Education			-0.019*			
			(0.006)			
			p=0.002			
Income				-0.000*		
				(0.000)		
				p=0.020		
Ideology (very liberal -> very conservative)					0.021*	
					(0.006)	
					p<.001	
Party ID (Democrat -> Republican)						0.019*
						(0.004)
						p<.001
intercept	0.148*	0.051	0.109*	0.072*	-0.016	-0.012
	(0.043)	(0.027)	(0.024)	(0.014)	(0.017)	(0.008)
N	1,007	1,107	2,114	2,114	2,084	2,112
R^2	.03	.00	.01	.01	.01	.02

Robust standard errors in parentheses; coefficients statistically significant at *$p < .05$

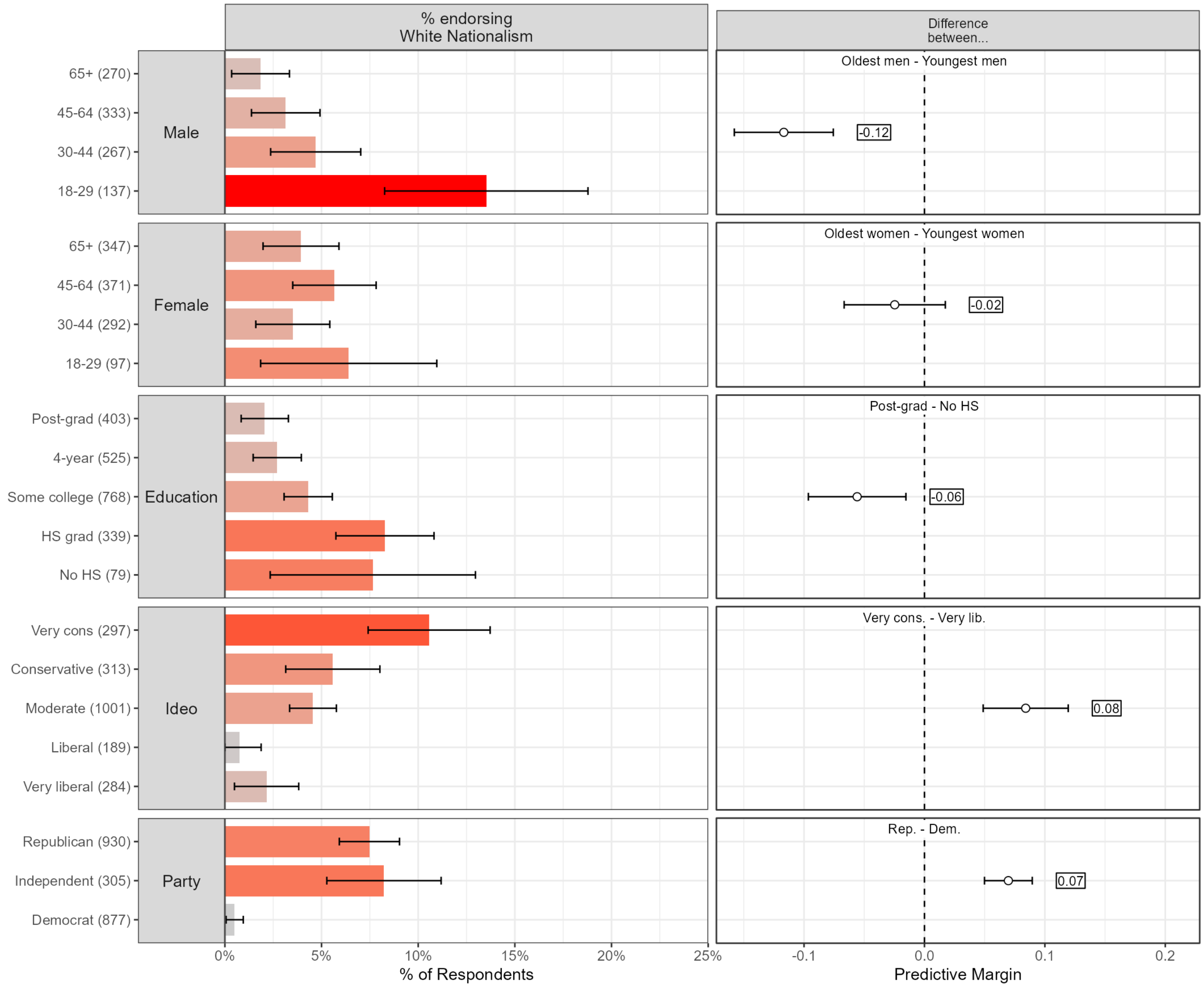

Fig. S5. Replication of Figure 2 in the text with data from Study 2 (where data are available)

Robustness of relationships to acquiescence bias (Study 3). A concern is that the relationships reported in Study 1 and Study 2 may be artifacts of acquiescence bias, which is more common among certain groups in the population, including younger, less educated, and more politically extreme respondents (5). We ruled out this concern using the survey experimental data collected in Study 3 as described in *SI Appendix* Section F.

Following (5), we created an indicator variable scored 1 if (as in Study 1 and Study 2) our white nationalism question was positive-keyed and asked "Are you a SUPPORTER of the white nationalist movement?" and scored -1 if it was negative-keyed and asked "Are you an OPPONENT of the white nationalist movement?" We then ran a series of regressions where the white nationalism measure was regressed the covariate, an indicator for the reverse-keyed item, and an interaction term between the covariate and the indicator. As described in (5), this coding and specification process produces coefficients on the covariates' constituent terms that by construction are estimates of the relationships that are free of any acquiescence bias.

As shown in Table S19, statistically significant relationships between white nationalist support

Table S19. Demographic and political correlates of support for white nationalist views corrected for acquiescence bias (Study 3; source of significance tests reported in text)

	age among men	age among women	education	income	ideology	party ID	vote	conspiracy beliefs
Age	-0.001<br>(0.001)<br>*p*=0.160	0.001<br>(0.001)<br>*p*=0.323						
Education			-0.007<br>(0.005)<br>*p*=0.149					
Income				-0.000*<br>(0.000)<br>*p*=0.047				
Ideology (very liberal -> very conservative)					0.044*<br>(0.006)<br>*p*<.001			
Party ID (Democrat -> Republican)						0.054*<br>(0.008)<br>*p*<.001		
Vote intention (Trump = 1, Harris = 0)							0.127*<br>(0.016)<br>*p*<.001	
Conspiracy beliefs scale								0.044*<br>(0.007)<br>*p*<.001
includes indicator variable for negative-keyed item	x	x	x	x	x	x	x	x
includes interaction term between covariate and indicator variable for negative-keyed item	x	x	x	x	x	x	x	x
N	788	900	1,697	1,671	1,678	1,696	1,564	1,694
R^2	.02	.05	.03	.03	.06	.06	.08	.05

Robust standard errors in parentheses; coefficients statistically significant at $^*p < .05$

and ideology, party ID, vote intention, conspiracy beliefs, and income persisted after accounting for any acquiescence bias (all $p < .05$). Near-significant negative relationships were found between white nationalist support and age among men ($p = .16$) and education ($p = .15$).

SI section H: Robustness Checks and Sensitivity Analysis (Study 1)

Our results should be interpreted as descriptive, rather than yielding causal claims. Here we re-analyze the data from Study 1 to undertake (1) robustness checks with multiple regression to determine the extent to which relationships between predictors and support for white nationalism persist in the *ceteris paribus* context, and (2) sensitivity analyses to characterize how vulnerable our bivariate results are to omitted unobservable confounders. We note that these tests do not exhaust the circumstances under which the significant bivariate relationships described in the main text might be rendered spurious. Other types of threats to causal inference may be present, including selection bias or reverse causality.

Robustness checks with multiple regression. We investigate the extent to which our key bivariate findings are robust to confounding by observables with multiple regressions in which white nationalist support is specified as the dependent variable (Table S20). We note here that the inclusion of some of these controls may introduce additional confounding due to post-treatment bias.

We begin with models including all demographic and political predictors displayed individually in Figure 2 to ascertain their strength in the context of *ceteris paribus*. Because of anticipated collinearity among the three political predictors (ideology, party identification, and belief in conspiracy theories), they are entered individually in Models 1 through 3, respectively. In each of these models, all predictors that were significant in the bivariate context (see Figure 2) remain so in the multiple regression context, except for income. Model 4 includes all three political predictors, and as expected their multicollinearity substantially reduces the magnitude and significance of their associations. The significance of all other predictors persists in this more saturated model.

In Model 5, we explore whether the three statistically significant findings regarding our theories—that white nationalist support is predicted by personal hardships, local social distress, and online-only friendships—–hold when white nationalist support is modeled as a function of all three of these factors simultaneously. All of these factors remain statistically significant in this model.

Finally in Model 6, *all* of these factors—demographics, political views, and the three theoretically-motivated predictors—–are included as regressors. Here, statistical significance is reduced somewhat on local social distress (which is now significant at the lower threshold of $p < .10$). All other coefficients remain statistically significant and all predictors remain signed in the same directions as in the bivariate analyses.

Table S20. Robustness checks with observable predictors

Dependent Variable:	Supporter of white nationalist movement					
Model:	(1)	(2)	(3)	(4)	(5)	(6)
Variables						
Age (years)	0.000	0.000	0.000	0.000		0.000
	(0.000)	(0.000)	(0.000)	(0.000)		(0.000)
Male	0.163***	0.166***	0.171***	0.170***		0.171***
	(0.031)	(0.031)	(0.031)	(0.031)		(0.030)
Age (years) × Male	-0.003***	-0.003***	-0.003***	-0.003***		-0.003***
	(0.001)	(0.001)	(0.001)	(0.001)		(0.001)
Educational attainment	-0.015***	-0.016***	-0.016***	-0.014***		-0.013***
	(0.004)	(0.004)	(0.004)	(0.004)		(0.004)
Family income	-0.000	-0.000	0.000	-0.000		0.000
	(0.000)	(0.000)	(0.000)	(0.000)		(0.000)
Attendance of relig svcs	-0.011***	-0.013***	-0.013***	-0.011***		-0.010***
	(0.003)	(0.003)	(0.003)	(0.003)		(0.003)
Ideology (liberal to conservative)	0.015***			0.008		0.009
	(0.004)			(0.006)		(0.006)
Party ID (Democrat to Republican)		0.017***		0.004		0.004
		(0.006)		(0.008)		(0.007)
Conspiracy beliefs (0-1 scale)			0.057***	0.041**		0.043**
			(0.016)	(0.017)		(0.017)
# of recent hardships					0.012**	0.009**
					(0.005)	(0.005)
County social distress score					0.013***	0.009*
					(0.005)	(0.005)
# of best 3 friends known online					0.025***	0.022***
					(0.005)	(0.005)
Constant	0.105***	0.117***	0.120***	0.086**	0.063***	0.050
	(0.033)	(0.033)	(0.031)	(0.034)	(0.005)	(0.035)
Observations	2,530	2,530	2,530	2,530	2,530	2,530
R^2	0.044	0.043	0.045	0.047	0.018	0.058
Adjusted R^2	0.042	0.041	0.042	0.043	0.016	0.054

IID standard errors in parentheses
*Signif. Codes: ***: 0.01, **: 0.05, *: 0.10*

Sensitivity analysis. We investigate the extent to which the three statistically significant findings in Table S20, Model 6 regarding our theories—that support for white nationalism is predicted by personal hardships, local social distress, and online-only friendships—are sensitive to confounding by unobserved omitted variables. We implement a widely used sensitivity analysis technique (7) with the `sensemakr` package (8), which builds on methods developed by (9–11).

This technique computes "robustness values," defined as the minimum joint strength of confounding required to reduce an estimated association of interest to zero or render it statistically insignificant. The joint strength of confounding is measured in terms of the partial R^2—the proportion of residual variance in the treatment and outcome that could be jointly explained by unobserved confounders. Robustness values can be expressed as multiples of the explanatory power (partial R^2) of an observed benchmark predictor, which helps contextualize how plausible it is that unobserved confounding could overturn the study's conclusions. We examine the sensitivity of our results in Table S20, Model 6 by using educational attainment as our benchmark covariate, given its persistence as a significant predictor across the multiple regression models.

Table S21. Relationship between a hypothetical omitted confounder, support for white nationalism, and each predictor (columns), expressed as multiples of the explanatory power of educational attainment, required to weaken the predictor's estimated association so that it no longer reaches the inference thresholds shown in the left-most column.

Threshold	# of recent hardships	Local social distress	# of online-only friends
Reduce to zero / Cross the null	35.4	9.1	19.1
90% confidence level	6.6	1.0	12.6
95% confidence level	0.6	-	11.4
99% confidence level	-	-	8.9

Table S21 summarizes the sensitivity of our three statistically significant theoretical findings in Table S20, Model 6 in terms of how many times stronger than educational attainment an omitted variable would need to be to reduce our estimates on these three predictors to zero (i.e. to "cross the null") or to render them statistically insignificant at $\alpha = 0.10$, $\alpha = 0.05$, and $\alpha = 0.01$:

- *Number of recent personal hardships* is somewhat sensitive to omitted variable bias. The association between personal hardships and support for white nationalism in Table S20, Model 6 is already not statistically significant at the 99% level, and an omitted variable that is about half as strongly related to both personal hardships and support for white nationalism as education is would be sufficient to bring the association below the 95% significance threshold.
- The association in Table S20, Model 6 between *local social distress* and support for white nationalism is quite sensitive to omitted variable bias. An omitted variable that is about as strongly related to both local social distress and support for white nationalism as education is would be sufficient to reduce the association below the 90% significance level.
- *Online-only friendships* is the predictor least sensitive to omitted variable bias. An omitted variable would need to be almost 19.1 times as strongly related to both online-only friendships and support for white nationalism as is education to eliminate the positive coefficient in Table S20, Model 6, and about 11.4 times as strongly related to reduce the association below the 95% significance level.

SI section I:
Assessing evidence for strain, status threat, and online mobilization theories (Study 1)

Figure 3 in the main text plots associations between white nationalist views and covariates associated with strain, status threat, and online mobilization theories. As in Figure 2, these are generated with separate bivariate regressions of white nationalist support on each covariate. We again run these regressions two ways. To generate the predictions displayed in Figure 3, we regress white nationalism support on categorical versions of these predictors (Tables S22, S24, S26). To perform the significance tests reported in the text on the associations between these predictors and white nationalism support, we regress support on continuous versions of the predictors (Tables S23, S25, S27). In addition, we show that personal hardships are positively and significantly correlated with county distress (Table S28).

Table S22. Strain theories (as categorical predictors; source of predictions displayed in Figure 3)

	personal hardships	occupational growth (historical)	occupational growth (projected)	county distress
intercept	0.047*	0.047*	0.071*	0.040*
	(0.006)	(0.011)	(0.011)	(0.008)
Recent personal hardships (excluded category: none)				
1 hardship	0.023			
	(0.013)			
2 hardships	0.060*			
	(0.022)			
3 or more hardships	0.036			
	(0.023)			
Occupational growth quartile, historical (excluded category: bottom quartile)				
Second quartile		0.027		
		(0.028)		
Third quartile		0.023		
		(0.023)		
Top quartile		0.027		
		(0.023)		
Projected growth in respondent's occupation (excluded category: decline)				
Average (3-4%)			-0.010	
			(0.020)	
Faster (5-7%)			-0.018	
			(0.017)	
Much Faster (8%+)			-0.025	
			(0.018)	
County social distress quartile (excluded category: lowest quartile)				
Second quartile				0.042*
				(0.016)
Third quartile				0.027
				(0.014)
Highest quartile				0.040*
				(0.015)
N	3227	1430	2117	3220
R^2	0.01	0.00	0.00	0.00

Robust standard errors in parentheses; coefficients statistically significant at $^*p < .05$

Table S23. Strain theories (as continuous predictors; source of significance tests reported in text)

	personal hardships	occupational growth (historical)	occupational growth (projected)	county distress	distress and hardships
# of recent personal hardships	0.015*				0.014*
	(0.005)				(0.006)
	p=.007				*p*=.011
Occupational growth quartile, historical		0.040			
		(0.034)			
		p=.243			
Projected growth in respondent's occupation			-0.208		
			(0.147)		
			p=.158		
County social distress score				0.023*	0.021*
				(0.010)	(0.010)
				p=.021	*p*=.031
intercept	0.054*	0.058*	0.067*	0.075*	0.062*
	(0.006)	(0.010)	(0.009)	(0.007)	(0.008)
N	3,227	1,430	2,117	3,220	3,220
R^2	.00	.00	.00	.00	.01

Robust standard errors in parentheses; coefficients statistically significant at *$p < .05$

Table S24. Status threat theories (as categorical predictors; source of predictions displayed in Figure 3)

	change in white pop %	white pop %	metropolitan status
Variables			
intercept	0.063*	0.064*	0.064*
	(0.012)	(0.011)	(0.012)
change in county % white population, 2009-2019 (excluded category: lowest quartile)			
2nd quartile	-0.007		
	(0.016)		
3rd quartile	-0.011		
	(0.016)		
Top quartile	0.036		
	(0.019)		
county % white population, 2019 (excluded category: lowest quartile)			
2nd quartile		-0.036*	
		(0.013)	
3rd quartile		0.022	
		(0.018)	
Top quartile		0.025	
		(0.018)	
Metropolitan status (excluded category: central metro)			
Suburban Metro			-0.002
			(0.018)
Small Metro			0.000
			(0.015)
Non-metro			0.021
			(0.019)
N	3,220	3,220	3,220
R^2	0.01	0.01	0.00

Robust standard errors in parentheses; coefficients statistically significant at *p<.05

Table S25. Status threat theories (as continuous predictors; source of significance tests reported in text)

	change in white pop %	white pop %	metropolitan status
change in county % white population, 2009-2019	0.004		
	(0.002)		
	p=0.078		
county % white population, 2019		0.001	
		(0.000)	
		p=0.070	
County urban/rural classification scale			0.004
			(0.004)
			p=0.312
intercept	0.084*	0.026	0.056*
	(0.012)	(0.022)	(0.013)
N	3,220	3,220	3,220
R^2	.00	.00	.00

Robust standard errors in parentheses; coefficients statistically significant at $^*p < .05$

Table S26. Online mobilization theories (as categorical predictors; source of predictions displayed in Figure 3)

	social media use	political activity on social media	online-only friendships
intercept	0.0703*	0.0642*	0.0617*
	(0.0125)	(0.0078)	(0.0069)
Social media use (excluded category: weekly or less)			
Daily	-0.0077		
	(0.0177)		
Multiple times per day	-0.0199		
	(0.0151)		
10 or more times per day	0.0291		
	(0.0226)		
Political activities on social media, past 24 hours (excluded category: None)			
One		0.0044	
		(0.0172)	
Two		-0.0014	
		(0.0197)	
Three or more		0.0172	
		(0.0174)	
# of 3 closest friends known online only (excluded category: none)			
One			0.0025
			(0.0179)
Two			0.0696
			(0.0421)
All three			0.1286*
			(0.0588)
N	3,161	3,227	2,985
R^2	0.00	0.00	0.01

Robust standard errors in parentheses; coefficients statistically significant at $^*p < .05$

Table S27. Online mobilization theories (as continuous predictors; source of significance tests reported in text)

	social media use	political activity on social media	online-only friendships
Frequency of social media use	0.003<br>(0.005)<br>*p*=0.594		
Social media political activities, past 24 hrs		0.003<br>(0.004)<br>*p*=0.476	
# of best 3 friends known online only			0.032*<br>(0.013)<br>*p*=0.012
intercept	0.056*<br>(0.017)	0.065*<br>(0.007)	0.059*<br>(0.007)
N	3,161	3,227	2,985
R^2	.00	.00	.01

Robust standard errors in parentheses; coefficients statistically significant at *$p < .05$

Table S28. Personal hardship and county distress (total hardships predicted by county distress)

	continuous	quartiles
County social distress score	0.096*	
	(0.037)	
	p=0.010	
County social distress quartile (excluded category: lowest quartile)		
Second quartile		0.197*
		(0.067)
		p=0.003
Third quartile		-0.000
		(0.060)
		p=1.000
Highest quartile		0.253*
		(0.070)
		p=0.000
N	3,220	3,220
R^2	0.00	0.01

Robust standard errors in parentheses; coefficients statistically significant at *$p < .05$

SI section J: Occupational classification and measures of occupational insecurity (Study 1)

To measure the occupational insecurity of our respondents, we relied on the U.S. Department of Labor's O*NET projections for 2019 to 2029, which describe the expected change in the number of jobs by occupation (12).

Our survey module asked respondents to describe either their current or most recent job with two open-ended questions: "What kind of work do you normally do?" and "What kind of place do you work for?" Three research assistants used these responses to classify all survey participants' occupations by Standard Occupational Classification (SOC) code using the O*NET website (http://onetonline.org). We had monthly check-ins with the coders to discuss their progress and resolve discrepancies in the codes.

We merge survey participants' SOC codes with 10-year O*NET projections describing how many jobs are projected to be added or lost in each SOC-classified occupation. We rely on O*NET's "Projected Growth (2019-2029)" metric, expressed as percent changes in the number of projected new jobs in the SOC category. In our analysis, we collapsed O*NET's "decline," "little or no change," and "slower than average" categories together into a "decline" category, which together represent a group of occupations that are projected to grow by less than 2%.

SI section K: Sources and generation of county-level data (Study 1)

Data sources. Our analysis relies on several contextual measures of demographic change and social distress measured at the county-level. To create these predictors, we relied on the following datasets:

- **Poverty rate.** Source: (13), "United States Summaries: US and All States and Counties."
  - Available as Excel spreadsheet (`est20all.xls`); converted by authors to Stata datafile `poverty.dta`.
- **Unemployment rate.** Source: (14), "Labor force data by county, 2021 annual averages."
  - Available as Excel spreadsheet (`laucnty21.xlsx`); converted by authors to Stata datafile `unemployment.dta`.
- **Drug and alcohol mortality rate.** Source: (15); datafile produced by authors via interactive point-and-click commands with website.
  - Keep all options on data request interface at defaults except:
    - Organize table layout: choose group results by County and check "age adjusted rate"
    - Select year and month: Highlight 2016, 2017, 2018, 2019 and 2020
    - Select cause of death:
      - Choose "Drug/Alcohol Induced Causes"
      - Keep "All Causes of Death" highlighted
    - Check "Export Results"
  - Available as .txt file ("Underlying Cause of Death, 1999-2020.txt"; NB: filename refers to data source, not this particular dataset); converted by authors to Stata datafile `substance.mortality.dta`.
- **County overall population change.** Source: (16).
  - Available as an Excel spreadsheet (`-est2019-alldata.csv`); county population change rate calculated by dividing POPESTIMATE2019 field by POPESTIMATE2010 field. Converted to Stata datafile `pop.change.dta`.
- **County white population levels and change.** Source: (17); database compiled from the US Census Bureau's American Community Survey (ACS) using the 2009 and 2019 ACS 5-year datasets.
  - Available as two files (`nhgis0003_ds196_20095_county.csv` and `nhgis0003_ds244_20195_county.csv`)
  - Preparation of this file was conducted using commands in the do-file `prepare.acs.data.do`. Final Stata datafile is `wht.pop.change.dta`.
- **Urban-rural county classification.** Source: (18).
  - Available as an Excel spreadsheet (`NCHSURCodes2013.xlsx`). Column of interest is "2013 Code". Converted to Stata datafile `nchscountyurbanruralcodes.dta`.

Creation of county distress measure. We constructed our measure of county distress by extracting the first factor derived from a factor analysis on four measures found in the first four of the datasets described above:

- Age-adjusted mortality rate from drugs and alcohol: `cty_drugalc_ageadjrate` (from `substance.mortality.dta`).
- County population change, 2010 to 2019: `cty_pop_change_2010_2019` (from `pop.change.dta`.
- County poverty rate, 2020: `cty_povrate_2020` (from `poverty.dta`).
- County unemployment rate, 2021: `cty_unemp_rate_2021` (from `unemployment.dta`).

SI section L: Estimates giving rise to age- and sex-specific trends in support for white nationalism in Figure 4 (Studies 1 and 2)

To generate Figure 4, we pooled data from Studies 1 and 2 on all respondents' age, sex, and support for white nationalism. Analysis was limited to those identifying as either male or female. We then ran the following regression in which white nationalist support (scored zero-one) among individuals of j years of age was regressed on an an intercept term for whether they were interviewed in Study 1, an indicator variable for female, fixed effects for each year j of age, and their interaction:

$$\begin{aligned} \text{white nationalist support} = {} & \alpha\, \mathbb{I}\Big(\text{interviewed in Study 1}\Big) + \beta\, \mathbb{I}\Big(\text{female}\Big) \\ & + \sum_j \delta_j\, \mathbb{I}\Big(\text{age}_j\Big) + \sum_j \gamma_j\, \mathbb{I}\Big(\text{age}_j \times \text{female}\Big) + \varepsilon \end{aligned}$$

We used the estimated equation to generate predicted values for each gender and age combination, which we converted into percentages and plotted as points in Figure 4. We used lowess smoothers at a span of .8 to draw the gender-specific age trends shown in the figure.

These patterns were consistent across Study 1 and Study 2, as shown in Figure S6 below, which replicates Figure 4 separately with the data from each of the two studies.

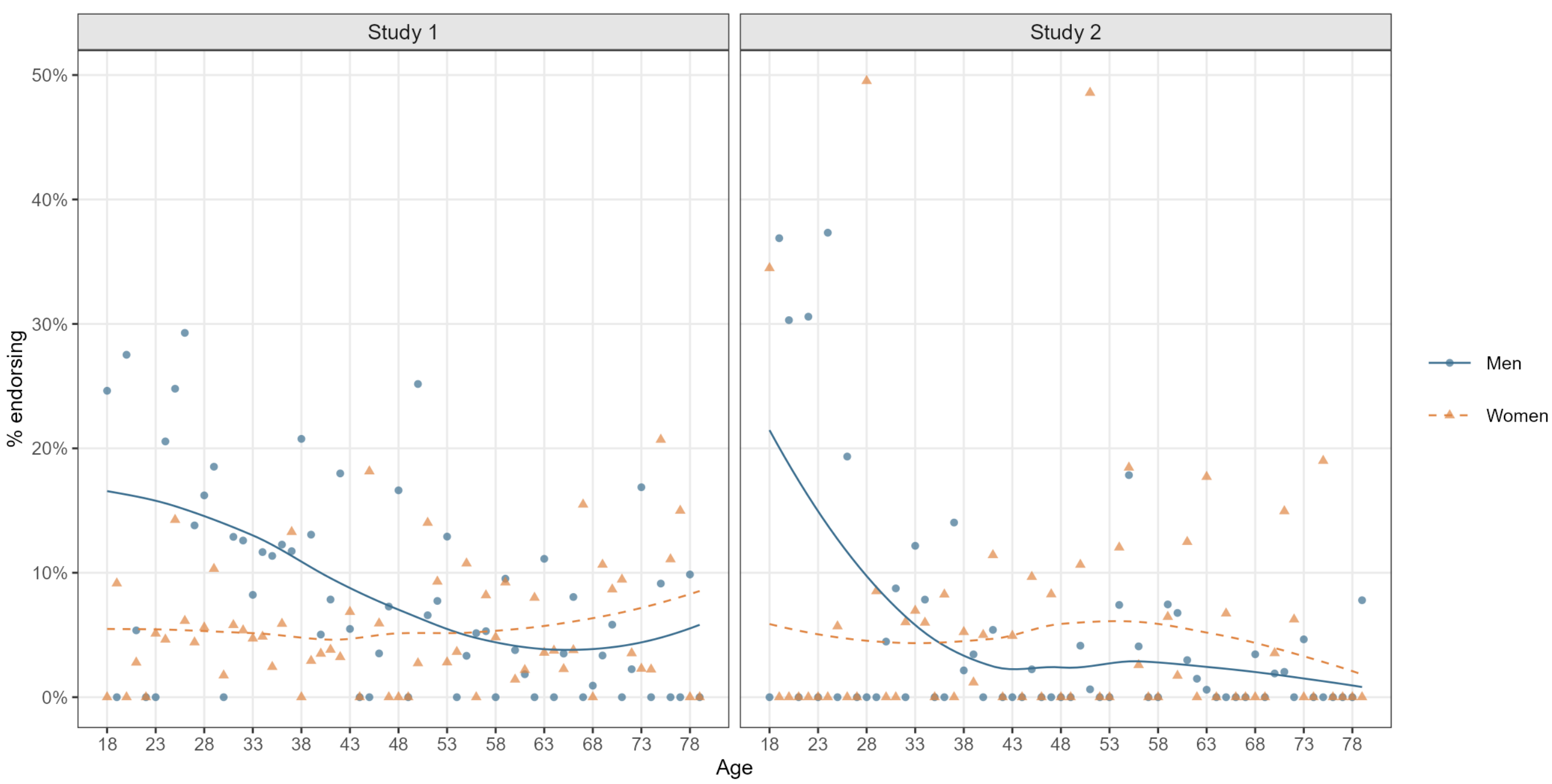

Fig. S6. Replication of Figure 4 separately with data from Study 1 and Study 2

References

1. Schaffner B, Ansolabehere S, Luks S (2022) Cooperative Election Study Common Content, 2021. *Harvard Dataverse* 1.
2. Westwood SJ, Grimmer J, Tyler M, Nall C (2022) Current research overstates American support for political violence. *Proceedings of the National Academy of Sciences* 119(12):e2116870119.
3. Alvarez RM, Atkeson LR, Levin I, Li Y (2019) Paying attention to inattentive survey respondents. *Political Analysis* 27(2):145–162.
4. Read B, Wolters L, Berinsky AJ (2022) Racing the clock: Using response time as a proxy for attentiveness on self-administered surveys. *Political Analysis* 30(4):550–569.
5. Hill SJ, Roberts ME (2023) Acquiescence bias inflates estimates of conspiratorial beliefs and political misperceptions. *Political Analysis* 31(4):575–590.
6. Decoster J, Claypool HM (2004) A meta-analysis of priming effects on impression formation supporting a general model of informational biases. *Personality and Social Psychology Review* 8(1):2–27.
7. Cinelli C, Hazlett C (2020) Making sense of sensitivity: Extending omitted variable bias. *Journal of the Royal Statistical Society Series B: Statistical Methodology* 82(1):39–67.
8. Cinelli C, Ferwerda J, Hazlett C (2024) sensemakr: Sensitivity analysis tools for ols in r and stata. *Observational Studies* 10(2):93–127.
9. Rosenbaum PR, Rubin DB (1983) Assessing sensitivity to an unobserved binary covariate in an observational study with binary outcome. *Journal of the Royal Statistical Society: Series B (Methodological)* 45(2):212–218.
10. Imbens GW (2003) Sensitivity to exogeneity assumptions in program evaluation. *American Economic Review* 93(2):126–132.
11. Blackwell M (2014) A selection bias approach to sensitivity analysis for causal effects. *Political Analysis* 22(2):169–182.
12. US Department of Labor (2022) O*NET - The Occupational Information Network (https://www.onetonline.org/). Accessed April 2022.
13. US Census Bureau (2021) Small area income and poverty estimates program 2020: Poverty and median household income estimates. (https://www.census.gov/data/datasets/2020/demo/saipe/2020-state-and-county.html). Accessed Oct 22, 2022.
14. US Bureau of Labor Statistics (2022) Local area unemployment statistics; labor force data by county, 2021 annual averages. (https://www.bls.gov/lau/#tables). Accessed Oct 22, 2022.
15. US Centers for Disease Control (2022) Cdc wonder database. (https://wonder.cdc.gov/ucd-icd10.html). Accessed Oct 22, 2022.
16. US Census Bureau (2020) Population, population change, and estimated components of population change: April 1, 2010 to July 1, 2019 (co-est2019-alldata). (https://www.census.gov/data/datasets/time-series/demo/popest/2010s-counties-total.html). Accessed Oct 22, 2022.
17. Manson S, Schroeder J, Riper DV, Kugler T, Ruggles S (2022) IPUMS National Historical Geographic Information System: Version 17.0 [dataset]. Accessed Oct 22, 2022.
18. National Center for Health Statistics (2013) NCHS urban-rural classification scheme for counties. (https://www.cdc.gov/nchs/data_access/urban_rural.htm#Data_Files_and_Documentation). Accessed Oct 22, 2022.
