# Supplementary material for: Predictors and prevalence of support for white nationalism in the United States
Source: Nature. 2026 Sep 16;657(8133):964–72. doi: 10.1038/s41586-026-11018-0 (PMC13600921; doi:10.1038/s41586-026-11018-0)
Supplement: Supplementary file 2 [file 41586_2026_11018_MOESM2_ESM.pdf]

nature portfolio

Corresponding author(s): Patrick J. Egan

Last updated by author(s): Jul 15, 2026

Reporting Summary

Nature Portfolio wishes to improve the reproducibility of the work that we publish. This form provides structure for consistency and transparency in reporting. For further information on Nature Portfolio policies, see our Editorial Policies and the Editorial Policy Checklist.

Statistics

For all statistical analyses, confirm that the following items are present in the figure legend, table legend, main text, or Methods section.

n/a	Confirmed	
☐	☒	The exact sample size (n) for each experimental group/condition, given as a discrete number and unit of measurement
☐	☒	A statement on whether measurements were taken from distinct samples or whether the same sample was measured repeatedly
☐	☒	The statistical test(s) used AND whether they are one- or two-sided<br>*Only common tests should be described solely by name; describe more complex techniques in the Methods section.*
☐	☒	A description of all covariates tested
☒	☐	A description of any assumptions or corrections, such as tests of normality and adjustment for multiple comparisons
☐	☒	A full description of the statistical parameters including central tendency (e.g. means) or other basic estimates (e.g. regression coefficient) AND variation (e.g. standard deviation) or associated estimates of uncertainty (e.g. confidence intervals)
☐	☒	For null hypothesis testing, the test statistic (e.g. F, t, r) with confidence intervals, effect sizes, degrees of freedom and P value noted<br>*Give P values as exact values whenever suitable.*
☒	☐	For Bayesian analysis, information on the choice of priors and Markov chain Monte Carlo settings
☒	☐	For hierarchical and complex designs, identification of the appropriate level for tests and full reporting of outcomes
☒	☐	Estimates of effect sizes (e.g. Cohen's d, Pearson's r), indicating how they were calculated

Our web collection on statistics for biologists contains articles on many of the points above.

Software and code

Policy information about availability of computer code

Data collection	Our data comes from three vendors who collected the data via web-based surveys: (Study 1) The Cooperative Election Study (CES), survey conducted by the YouGov firm with a national opt-in sample matched to US demographics; (Study 2) The National Opinion Research Corporation (NORC)'s AmeriSpeak Panel, a national probability sample recruited offline; and (Study 3) CloudResearch Connect, a national quota-based sample. Data for Studies 1 and 2 were collected directly by our vendors; we collected data for Study 3 via the Qualtrics platform (using features of the platform that were available in November 2024).
Data analysis	We used the R statistical software package (version 4.6.1) for all data analysis and data visualizations. The full list of packages used is provided in the replication archive. Code for reproducing the analysis and data visualizations is available at PJE's Harvard Dataverse site [https://doi.org/10.7910/DVN/J6G51T]

For manuscripts utilizing custom algorithms or software that are central to the research but not yet described in published literature, software must be made available to editors and reviewers. We strongly encourage code deposition in a community repository (e.g. GitHub). See the Nature Portfolio guidelines for submitting code & software for further information.

Data

Policy information about availability of data

All manuscripts must include a data availability statement. This statement should provide the following information, where applicable:

- Accession codes, unique identifiers, or web links for publicly available datasets
- A description of any restrictions on data availability
- For clinical datasets or third party data, please ensure that the statement adheres to our policy

All survey data and contextual data, and code for reproducing statistical results and figures will be made freely available upon publication at PJE's Harvard Dataverse repository page (https://doi.org/10.7910/DVN/J6G51T).

Research involving human participants, their data, or biological material

Policy information about studies with human participants or human data. See also policy information about sex, gender (identity/presentation), and sexual orientation and race, ethnicity and racism.

Reporting on sex and gender	In our three studies, we analyze rates of endorsement of white nationalist ideology on the basis of self-reported gender according to the survey items fielded by our vendors:<br><br>Study 1: women = 1,727; men = 1,460; other = 40.<br>Study 2: women = 1,122; men = 1,024<br>Study 3: women = 1,349; men = 1,224; nonbinary = 10; no answer = 2
Reporting on race, ethnicity, or other socially relevant groupings	Our data collection was limited to US adults who identified as white and did not identify as Hispanic. Our analyses are limited to bivariate analyses and thus no controls are used.
Population characteristics	All participants were non-Hispanic white residents of the US (50 states + DC) aged 18 years or older.<br>In Study 1: 51% female; 39% with 4-year college degree or more; 45% Republican, 36% Democrat, 16% Independent. Mean age: 51 years old.<br>In Study 2: 51% female, 40% with 4-year college degree or more; 46% Republican, 38% Democrat, 16% Independent. Mean age: 51 years old.<br>In Study 3: 52% female, 54% with 4-year college degree or more; 42% Republican, 46% Democrat, 12% Independent. Mean age: 45 years old.<br>(Percentages are rounded and may not add up to 100%.)
Recruitment	Study 1: A national sample recruited online via an opt-in panel<br>Study 2: A national probability sample recruited offline<br>Study 3: A national quota-based sample recruited online via opt-in procedures
Ethics oversight	The study protocol was approved by the NYU Institutional Review Board.<br>Study 1: IRB-FY2021-4955; approved under exempt review August 20, 2021.<br>Study 2 & 3: IRB-FY2024-8846 (Study 2 approved May 8, 2024; Study 3 approved with modification to protocol November 4, 2024). Reviewed and approved following an expedited review as described at 45 CFR 46 110(b)[1], 7. Research on individual or group characteristics or behavior.

Note that full information on the approval of the study protocol must also be provided in the manuscript.

Field-specific reporting

Please select the one below that is the best fit for your research. If you are not sure, read the appropriate sections before making your selection.

☐ Life sciences ☒ Behavioural & social sciences ☐ Ecological, evolutionary & environmental sciences

For a reference copy of the document with all sections, see nature.com/documents/nr-reporting-summary-flat.pdf

Behavioural & social sciences study design

All studies must disclose on these points even when the disclosure is negative.

Study description	This is a quantitative study using survey data.
Research sample	The research samples for all three studies consist of non-Hispanic white adults in the United States, reflecting our goal of studying the predictors and prevalence of support for white nationalism in the non-Hispanic white adult population.
Sampling strategy	Study 1: A national sample recruited online via an opt-in panel

Sampling strategy	Study 2: A national probability sample recruited offline<br>Study 3: A national quota-based sample recruited online via opt-in procedures
Data collection	In all three studies, survey data were collected via a web-based interface, administered (at the participant's choice) by smartphone, tablet, or personal computer. No researcher was present during data collection.
Timing	Study 1: Data collected in 2021.<br>Study 2: Data collected in 2024.<br>Study 3: Data collected in 2024 and 2025.
Data exclusions	No participant data were excluded, except in analyses requiring covariates for which respondents did not provide valid responses.
Non-participation	Our vendors do not supply rates of non-response with their datasets.
Randomization	Studies 1 and 2 include one experimental condition in which half of respondents were to treatment and control groups at random.<br>Study 3 includes 8 experimental conditions that vary question wording and question order.

Reporting for specific materials, systems and methods

We require information from authors about some types of materials, experimental systems and methods used in many studies. Here, indicate whether each material, system or method listed is relevant to your study. If you are not sure if a list item applies to your research, read the appropriate section before selecting a response.

Materials & experimental systems

n/a	Involved in the study
☒	☐ Antibodies
☒	☐ Eukaryotic cell lines
☒	☐ Palaeontology and archaeology
☒	☐ Animals and other organisms
☒	☐ Clinical data
☒	☐ Dual use research of concern
☒	☐ Plants

Methods

n/a	Involved in the study
☒	☐ ChIP-seq
☒	☐ Flow cytometry
☒	☐ MRI-based neuroimaging

Plants

Seed stocks	*Report on the source of all seed stocks or other plant material used. If applicable, state the seed stock centre and catalogue number. If plant specimens were collected from the field, describe the collection location, date and sampling procedures.*
Novel plant genotypes	*Describe the methods by which all novel plant genotypes were produced. This includes those generated by transgenic approaches, gene editing, chemical/radiation-based mutagenesis and hybridization. For transgenic lines, describe the transformation method, the number of independent lines analyzed and the generation upon which experiments were performed. For gene-edited lines, describe the editor used, the endogenous sequence targeted for editing, the targeting guide RNA sequence (if applicable) and how the editor was applied.*
Authentication	*Describe any authentication procedures for each seed stock used or novel genotype generated. Describe any experiments used to assess the effect of a mutation and, where applicable, how potential secondary effects (e.g. second site T-DNA insertions, mosiacism, off-target gene editing) were examined.*
